# Characteristics of Effective Collaborative Care for Treatment of Depression: A Systematic Review and Meta-Regression of 74 Randomised Controlled Trials

**DOI:** 10.1371/journal.pone.0108114

**Published:** 2014-09-29

**Authors:** Peter A. Coventry, Joanna L. Hudson, Evangelos Kontopantelis, Janine Archer, David A. Richards, Simon Gilbody, Karina Lovell, Chris Dickens, Linda Gask, Waquas Waheed, Peter Bower

**Affiliations:** 1 Collaboration for Leadership in Applied Health Research and Care, Centre for Primary Care and Manchester Academic Health Science Centre, University of Manchester, Manchester, United Kingdom; 2 Health Psychology Section, Psychology Department, Institute of Psychiatry, King's College London, London, United Kingdom; 3 NIHR School for Primary Care Research, Centre for Primary Care and Manchester Academic Health Science Centre, University of Manchester, Manchester, United Kingdom; 4 School of Nursing, Midwifery & Social Work, University of Manchester, Manchester, United Kingdom; 5 Institute of Health Service Research, University of Exeter Medical School, University of Exeter, Exeter, United Kingdom; 6 Mental Health Research Group, Department of Health Sciences and Hull York Medical School, University of York, York, United Kingdom; 7 Lancashire Care NHS Foundation Trust, Preston, United Kingdom; University of Western Sydney, Australia

## Abstract

**Background:**

Collaborative care is a complex intervention based on chronic disease management models and is effective in the management of depression. However, there is still uncertainty about which components of collaborative care are effective. We used meta-regression to identify factors in collaborative care associated with improvement in patient outcomes (depressive symptoms) and the process of care (use of anti-depressant medication).

**Methods and Findings:**

Systematic review with meta-regression. The Cochrane Collaboration Depression, Anxiety and Neurosis Group trials registers were searched from inception to 9th February 2012. An update was run in the CENTRAL trials database on 29th December 2013. Inclusion criteria were: randomised controlled trials of collaborative care for adults ≥18 years with a primary diagnosis of depression or mixed anxiety and depressive disorder. Random effects meta-regression was used to estimate regression coefficients with 95% confidence intervals (CIs) between study level covariates and depressive symptoms and relative risk (95% CI) and anti-depressant use. The association between anti-depressant use and improvement in depression was also explored. Seventy four trials were identified (85 comparisons, across 21,345 participants). Collaborative care that included psychological interventions predicted improvement in depression (β coefficient −0.11, 95% CI −0.20 to −0.01, p = 0.03). Systematic identification of patients (relative risk 1.43, 95% CI 1.12 to 1.81, p = 0.004) and the presence of a chronic physical condition (relative risk 1.32, 95% CI 1.05 to 1.65, p = 0.02) predicted use of anti-depressant medication.

**Conclusion:**

Trials of collaborative care that included psychological treatment, with or without anti-depressant medication, appeared to improve depression more than those without psychological treatment. Trials that used systematic methods to identify patients with depression and also trials that included patients with a chronic physical condition reported improved use of anti-depressant medication. However, these findings are limited by the observational nature of meta-regression, incomplete data reporting, and the use of study aggregates.

## Introduction

Major depressive disorder accounted for 8.2% of years living with disability in 2010, making it the second leading direct cause of global disease burden [1]. People with depression and a chronic physical disease have worse health status than people with depression alone or people with any combination of chronic physical disease without depression [2].

Significant advances have occurred in primary care in recent years to improve the management of chronic disease, principally by introducing structured disease management programmes that draw on the Chronic Care Model [3]. The chronic care model promotes a more proactive, planned and population-based approach to disease management and has been instrumental in transforming ambulatory care in primary care [4]. The concept and components of the chronic care model are fully specified here: http://www.improvingchroniccare.org/. Depression shares with other chronic diseases many features that can be addressed by the chronic care model, such as multiple recurrent episodes [5], where successful management hinges on regular monitoring, care coordination, enhancing providers' expertise, and supporting patients to self-manage. Interventions that include at least one component of the chronic care model have been shown to improve clinical outcomes and the process of care for people with chronic disease, including depression [6].

‘Collaborative care’ is the most promising chronic care model-based strategy for improving care of depression. While the make-up of collaborative care interventions for treatment of depression vary, they typically include a multi-professional approach to patient care, structured management, scheduled patient follow-ups, and enhanced inter-professional communication [7]. A recent Cochrane review that included 79 randomised controlled trials (RCTs) and 24,308 participants conclusively showed that collaborative care is more effective than usual care for both depression and anxiety after treatment, and up to two years later [8]. There is also ample evidence that these benefits are cost effective [9].

However, while some authors suggest that there is now sufficient evidence about effectiveness and that research should now shift to implementation [10], collaborative care is a complex intervention and there is significant variation in the exact nature of the intervention between trials, as well as differences in patient populations, contexts, comparators, and design. A number of these factors have already been shown to be related to estimates of effect: setting (i.e. country), recruitment of patients using systematic or population health approaches (e.g. disease registers), using case managers with a mental health background, and regular clinical supervision of case managers [11]. There has since been considerable international expansion of collaborative care outside of the United States and extension of this care model to populations with depression and chronic physical disease. We have therefore used meta-regression with a comprehensive and updated data set of randomised controlled trials of collaborative care to identify factors associated with improvement in patient outcome (i.e. depressive symptoms) and/or the process of care (i.e. anti-depressant use). The results will be used to distinguish which features of collaborative care effectively improve patient outcomes and/or the process of care and which do not.

## Methods

**Figure 1:**
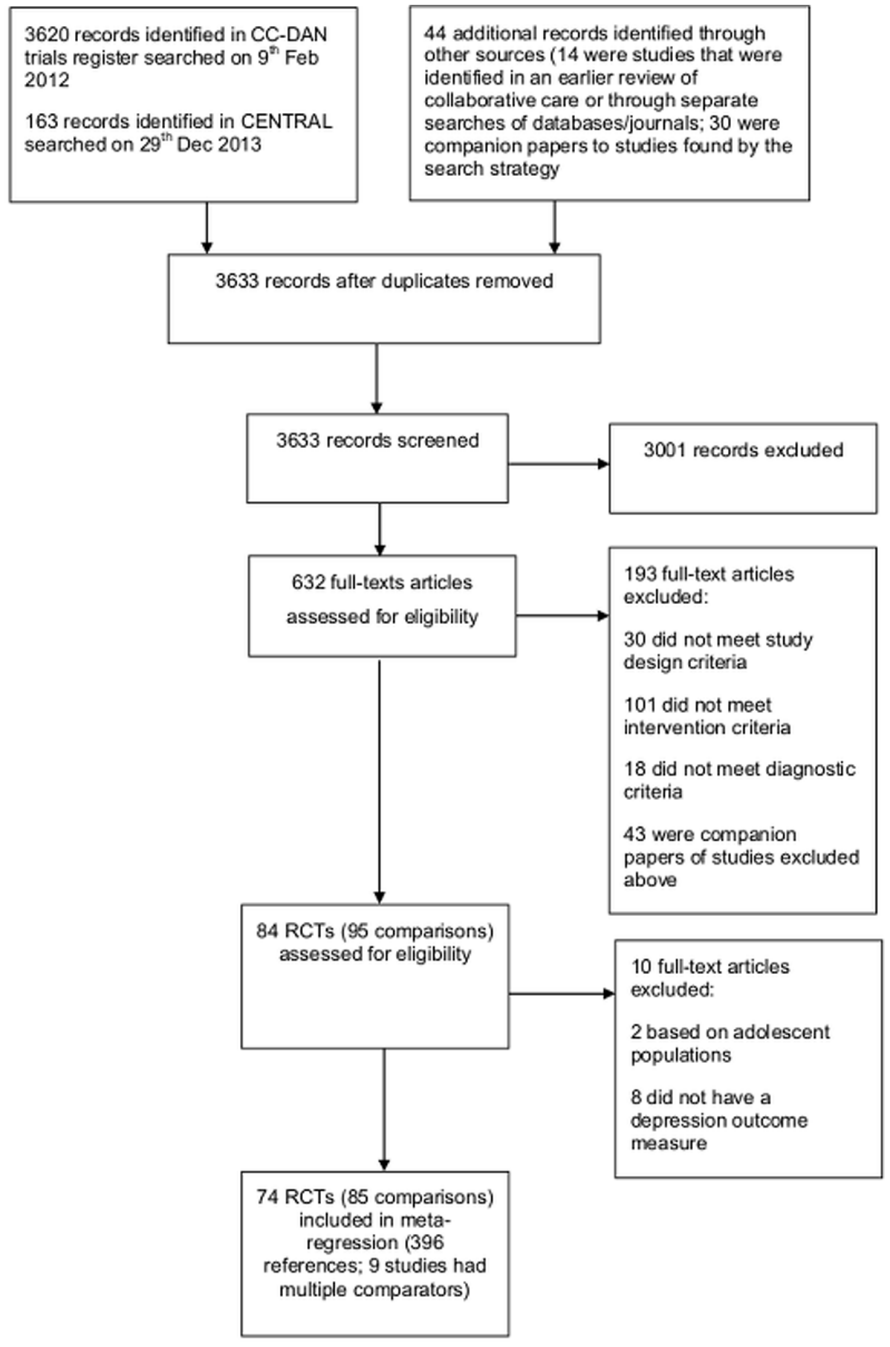
PRISMA Flow Diagram.

This systematic review and meta-regression is reported in accordance with the Preferred Reporting Items for Systematic Reviews and Meta-Analyses Statement (see Figure 1 and Checklist S1) [12].

### Information sources

The Cochrane Collaboration Depression, Anxiety and Neurosis Group (CC-DAN) trials registers (including both the references register and the studies register) were searched from inception to 9^th^ February 2012. The CC-DAN registers include Randomised Controlled Trials indexed in MEDLINE, EMBASE, PsycINFO, CENTRAL, World Health Organisation's trials portal (ICTRP), Clinicaltrials.gov, and CINAHL. Details of the search strategy used can be found in Archer et al [8]. The CENTRAL search used in the Cochrane review by Archer et al [8] was updated on 29^th^ December 2013 (see Methods S1 for search strategy). For the purposes of an update the comprehensive coverage of the CENTRAL database makes exhaustive searching of individual bibliographic databases unnecessary [13]. All reference lists of included studies and previously published reviews were checked.

### Characteristics of collaborative care and conceptual model to be tested

For the purposes of this review we used a definition of collaborative care derived from a systematic review of complex interventions for managing depression in primary care [7]. Collaborative care consists of four key criteria: multi-professional approach to patient care, structured management, scheduled patient follow-ups, and enhanced inter-professional communication (Table 1).

**Table 1:** Key characteristics of collaborative care.

**A multi-professional approach to patient care**
A general practitioner (GP) or family physician and at least one other health professional (e.g. nurse, psychologist, psychiatrist, pharmacist) were involved with patient care, usually acting as a case or care manager to coordinate and/or deliver care for the depressed person
**A structured management plan**
Evidence based guidelines or treatment protocols. Interventions could include both pharmacological (e.g. antidepressant medication) and non-pharmacological interventions (e.g. patient screening, patient and provider education, counselling, cognitive behaviour therapy)
**Scheduled patient follow-ups**
An organised approach to patient follow-up that could include one or more scheduled telephone or in-person follow-up appointments to provide specific interventions, facilitate treatment adherence, or monitor symptoms or adverse effects
**Enhanced inter-professional communication**
Mechanisms to facilitate communication between professionals caring for the depressed person: team meetings, case-conferences, individual consultation/supervision, shared medical records, patient-specific written or verbal feedback between care-givers

A key innovation arising from collaborative care is the introduction of a non-medical case or care manager who works with a medical practitioner and under the supervision of a mental health specialist to deliver and coordinate psychological treatment, and to monitor progress with psychological and/or pharmacological treatment [14]. While chronic disease management interventions that include one or more features of patient or provider education, feedback, and reminders can lead to improved disease control and adherence to guidelines [15] enhanced roles in primary care for case managers are seen as central to the provision of effective and integrated interventions for depression [16]. Based on current understanding about how the intervention might work and based on previous knowledge about mechanisms of change and active ingredients of collaborative care we tested 10 factors (study covariates) that could potentially moderate patient and process outcomes (Table 2).

**Table 2:** Study level covariates (N = 85 comparisons).

Characteristic	Description	N
Country		
US	Study setting	60
Non-US	Study setting	25
Recruitment method		
Systematic identification	Patients were referred into the study if they were: i) identified from a clinical database as having depression or ii) screened positive for depression on an outcome measure and/or diagnostic clinical interview	69
Referral by clinicians	Patients were referred into the study by their clinician	16
Patient sample		
Anti-depressant medication NOT inclusion criteria	Participants did not have to be on or willing to take anti-depressant medication as part of the study's inclusion criteria	62
Anti-depressant medication part of inclusion criteria	Participants had to be currently taking or willing to take anti-depressant medication as part of the study's inclusion criteria	23
Chronic physical health condition		
Present	Participants with a chronic physical health condition were actively recruited as part of the study's inclusion criteria	19
Absent	Participants with a chronic physical health condition were NOT actively recruited as part of the study's inclusion criteria	66
Case manager professional background		
Mental health professional	Trained in mental health disciplines outside of the context of the trial (i.e. psychologist, psychiatric nurse, social worker)	47
Non-mental health professional	No extensive training in mental health other than that provided by the trial	38
Intervention content		
Medication management	Intervention included a medication management plan to ensure optimal levels of adherence to pharmacotherapy. This does not represent degree of adherence (i.e. anti-depressant use).	38
Psychological or both	Intervention included a recognised psychological treatment model (i.e. behavioural activation, problem solving) either on its own or combined with medication.	47
Number of sessions		
Continuous variable	Based on number of planned sessions in the first six months. If the number of sessions differed based on the treatment modality offered (i.e. participants could choose between medication management or psychological therapy) then a mean score was calculated.	
Supervision frequency		
Ad hoc	No regular patterns of supervision.	29
Scheduled	Supervision occurred on a regular basis (i.e. weekly).	52
Not applicable	Applies to studies whose collaborative care intervention included only the primary care provider but the case manager was a certified mental health practitioner (e.g. psychiatrist, psychologist, psychotherapist).	4
Enhanced usual care		
Ordinal variable (based on summed score, 0 4)	See description provided in Methods S2	
Allocation concealment		
Low risk of bias	Coded according to Cochrane risk of bias tool	39
High risk of bias	Coded according to Cochrane risk of bias tool	4

### Eligibility criteria

Studies were included in this meta-analysis and meta-regression if they:

1) Were RCTs or clustered RCTs of collaborative care delivered in primary care settings or community settings. Primary care was defined as a person's first and ongoing contact point for health care [17].
2) Included adults over the age of 18 with a primary diagnosis of depression or mixed anxiety and depressive disorder according to clinical diagnosis or research assessment (observer interview or validated self-report measure). No restrictions on severity or chronicity of depression were made.
3) Compared the effectiveness of collaborative care with standard or enhanced usual care.

Standard usual care was defined as care routinely provided by primary care providers to patients with depression or mixed anxiety and depressive disorder. This could include onward referral to mental health teams or feedback on participants' depressive status if specified in the protocol.

Enhanced usual care was defined as care where any one or more of the following were present:

- The patient received additional resources (i.e. educational leaflets, lists of locally available resources, letter from research team with self-help advice) OR the patient had access to enhanced care systems (i.e. consultation-liaison, enhanced referral systems to psychology services, case reviews between health professionals, equal contact time with a health professional, medication management, patient-primary care provider electronic messaging system, personalised patient treatment plans from the principal investigator).
- Didactic training for the primary care provider.
- Primary care providers were supplied with manualised treatment algorithms or evidence based guidelines.
- Primary care providers received educational materials other than evidence based guidelines (e.g. educational DVD).

4) Measured change in self-reported or observer rated depression scores as a continuous or dichotomous measure (e.g. ≥50% decrease in symptom scores from baseline or remission)

AND/OR

Measured change in use of anti-depressant medication (e.g. proportion of patients taking medication or proportion of patients adhering appropriately to predefined criteria/guidelines), based on self-report or administrative records data.

### Study selection

We identified eligible studies included in the Cochrane review of collaborative care for depression and anxiety [8] and also from eligible studies identified by the updated search of the CENTRAL trials database. Three authors (JH, PB, PC) independently screened non-overlapping subsets of studies against the inclusion criteria for this meta-regression.

### Data extraction

#### Intervention content

Characteristics of collaborative and usual care intervention groups were independently extracted verbatim and coded using a standardised data extraction form and coding manual, specifically tailored to the content of collaborative care interventions (Methods S2).

#### Patient and process outcomes

The primary patient outcome was reduction in depressive symptoms as measured by observer or patient self-report. Outcomes were extracted for all reported follow-up time points (e.g. six months, twelve months). Most studies reported outcomes at six months follow-up and our analysis was therefore restricted to this time point to maximise both consistency and the number of studies included in the meta-regression. If eligible studies (n = 9) reported outcomes at follow-ups beyond six months we used short term follow-up data closest in time to six months. Where the studies reported two comparisons versus a control group, sample sizes were halved to avoid double counting.

To allow both continuous and dichotomous outcomes to be included in the same meta-regression, we translated dichotomous outcomes into standardised mean differences and standard errors using the *metaeff* Stata command [18].

Collaborative care improves the process of depression care, and use of anti-depressant medications may be a key driver for depressive outcomes. Anti-depressant use as a dichotomous process outcome was extracted; we used risk ratios with log-transformations applied [19].

When studies applied cluster randomisation procedures we accounted for increased Type I error rates by applying the “effective sample sizes” procedure outlined in the Cochrane Handbook [20]. An intraclass correlation coefficient (ICC) of 0.02 [21] was used and sensitivity analyses were performed to explore the impact of adjustments for clustering using an ICC of 0.00 and 0.05 [22]. We also used study sample sizes as a proxy for publication bias [23]. We explored using meta-regression whether there was an inverse relationship between study sample sizes and reported effect sizes. Allocation concealment, an important feature of trials known to reduce risk of bias, was assessed using a binary measure [24].

### Analysis

Stata's (Version 12 for Windows) *metan* [25] and *metareg* [26] commands were used to calculate an overall effect size estimate of collaborative care on depressive symptoms and anti-depressant medication use. Consistent with the recommendations of Thompson and Higgins [27], ten covariates were hypothesised to have an effect on these outcomes *a priori* (See Table 2) [11]. In contrast to Bower et al [11] we revised the conceptualisation of enhanced usual care to include an ordinal measure of enhanced usual care in place of primary care provider training (See Methods S2 for the coding and scoring of enhanced usual care).

We used a DerSimonian-Laird [28] random-effects model to calculate the overall effect of collaborative care, accounting for estimated heterogeneity. To quantify the estimated heterogeneity we used the *I^2^* index, which represents the percentage of estimated between-study variability in the total variability [29]. By convention *I^2^* values of 25% are considered low, 50% moderate, and 75% high [20]. The main analysis used random-effects meta-regression to estimate a regression coefficient with 95% CIs between study level covariates and outcomes for:

- Meta-regression model one (multivariable): study level covariates as predictors of depressive symptoms.
- Meta-regression model two (multivariable): study level covariates as predictors of anti-depressant use.
- Meta-regression model three (univariable; mechanisms of change): anti-depressant use as a predictor of depressive symptoms. This model tested whether use of anti-depressant medication predicted the treatment effect on depressive symptoms.

We explored the potential for confounding or collinearity across the 10 covariates using logistic regression analyses to perform pairwise comparisons. Shared variance between each pair was low (≤0.14), indicating that there is a very small risk of measured confounding or collinearity in our measured variables. To retain statistical power we identified covariates for testing in the multivariable meta-regression models by initially performing a series of separate univariable meta-regression analyses, using a significance criterion of p≤0.10. The p≤0.10 threshold was chosen to avoid prematurely discounting potentially important explanatory variables. Table 2 summarises all ten study level covariates included in both meta-regression models one and two.

## Results

### Characteristics of included studies

Seventy four trials met our inclusion criteria for the meta-regression (including 85 relevant comparisons, across 21,345 participants); 84 comparisons had data on depressive symptoms (across 21,284 participants), and 59 comparisons had data on anti-depressant use (across, 14, 465 participants). See Figure 1.

Of the 85 comparisons included in the meta-regression 25 (29%) were conducted outside the United States; only 4% were conducted in low to middle income countries (Table S1). Nineteen (22%) comparisons specifically recruited patients with chronic physical disease. As stated in the methods section trials had to meet the four criteria for collaborative care to be included in the review. Key factors that differentiated the type of interventions tested in the meta-regression were case manager background and content of the structured management plan. In 47 (55%) comparisons the case manager was a mental health practitioner, and in 38 (45%) comparisons case managers were drawn from a variety of non-mental health backgrounds. In 39 (46%) comparisons the intervention included both psychological therapy and antidepressant medication management; 40 (47%) included medication management alone; and 6 (7%) included psychological therapy alone. Table 3 describes characteristics of collaborative care used in the included trials. A reference list of included trials is presented in Results S1.

**Table 3:** Characteristics of collaborative care.

Author	Multi-professional approach to patient care		Structured management plan	Number of scheduled follow-ups	Enhanced inter professional communication	
	Medical professional	Case manager background			CM liaison with PCP	Supervision frequency
Adler 2004	Doctor	Non-mental health practitioner	Medication management	6	Shared note system	Scheduled
Araya 2003	Doctor	Mental health practitioner	Both	9	Shared note system	Ad hoc
Bartels 2004	Doctor	Mental health practitioner	Medication management	Not reported	Multiple methods	Not reported
Blanchard 1995	Doctor	Mental health practitioner	Both	12	Verbal	Ad hoc
Bogner 2008	Mixed	Mental health practitioner	Medication management	5	Liaison method	Scheduled
Bogner 2010	Mixed	Non-mental health practitioner	Medication management	5	Liaison method	Scheduled
Bogner 2012	Doctor	Non-mental health practitioner	Medication management	5	Verbal	Ad hoc
Bruce 2004	Doctor	Mental health practitioner	Both	15	Liaison method	Scheduled
Buszewicz 2011	Doctor	Non-mental health practitioner	Both	4	Verbal	Ad hoc
Capoccia 2004	Doctor	non-mental health practitioner	Medication management	10	Shared note system	Scheduled
Chaney 2011	Doctor	Non-mental health practitioner	Medication management	5	Multiple methods	Scheduled
Chew-Graham 2007	Doctor	Mental health practitioner	Both	11	Multiple methods	Scheduled
Ciechanowski 2004	Doctor	Mental health practitioner	Psychological therapy	9	Other	Scheduled
Ciechanowski 2010	Specialist	Mental health practitioner	Psychological therapy	9	Other	Scheduled
Cole 2006	Doctor	Non-mental health practitioner	Both	24	Liaison method	Ad hoc
Datto 2003	Doctor	Mental health practitioner	Medication management	6	Written communication	Scheduled
Davidson 2013	Doctor	Mental health practitioner	Both	11	Verbal	Scheduled
Dietrich 2004	Doctor	Mental health practitioner	Medication management	6	Written communication	Scheduled
Dwight-Johnson 2005	Doctor	Mental health practitioner	Both	7	Multiple methods	Scheduled
Dwight-Johnson 2010	Doctor	Mental health practitioner	Both	12	Liaison method	Scheduled
Dwight-Johnson 2011	Specialist	Mental health practitioner	Psychological therapy	8	Liaison method	Scheduled
Ell 2007	Doctor	Mental health practitioner	Both	12	Liaison method	Ad hoc
Ell 2008	Specialist	Mental health practitioner	Both	21	Multiple methods	Scheduled
Ell 2010	Doctor	Mental health practitioner	Both	18	Liaison method	Scheduled
Finley 2003	Doctor	Non-mental health practitioner	Medication management	8	Multiple methods	Scheduled
Fortney 2007	Doctor	Non-mental health practitioner	Medication management	26	Shared note system	Scheduled
Fritsch 2007	Doctor	Non-mental health practitioner	Medication management	6	Other	Ad hoc
Gensichen 2009	Doctor	Non-mental health practitioner	Both	13	Written communication	Ad hoc
Gjerdingen 2008	Doctor	Mental health practitioner	Medication management	12	Written communication	Ad hoc
Hedrick 2003	Doctor	Mental health practitioner	Both	Not reported	Shared note system	Scheduled
Huffman 2011	Doctor	Mental health practitioner	Medication management	4	Liaison method	Scheduled
Huijbregts 2013	Doctor	Non-mental health practitioner	Both	12	Share note system	Scheduled
Hunkeler 2000	Doctor	Non-mental health practitioner	Both	14	Liaison method	Scheduled
Katon 1995a	Doctor	Mental health practitioner	Both	2	Multiple methods	Not reported
Katon 1995b	Doctor	Mental health practitioner	Both	2	Multiple methods	Not reported
Katon 1996a	Doctor	Mental health practitioner	Both	10	Multiple methods	Scheduled
Katon 1996b	Doctor	Mental health practitioner	Both	10	Multiple methods	Scheduled
Katon 1999	Doctor	Mental health practitioner	Medication management	3	Multiple methods	Not reported
Katon 2001	Doctor	Mental health practitioner	Both	4	Multiple methods	Scheduled
Katon 2004	Doctor	Non-mental health practitioner	Both	7	Multiple methods	Scheduled
Katon 2010	Doctor	Non-mental health practitioner	Both	Not reported	Liaison method	Scheduled
Katzelnick 2000	Doctor	Non-mental health practitioner	Medication management	3	Multiple methods	Ad hoc
Kroenke 2010	Doctor	Non-mental health practitioner	Medication management	4	Other	Scheduled
Landis 2007	Doctor	Mental health practitioner	Medication management	9	Liaison method	Scheduled
Lobello 2010	Doctor	Non-mental health practitioner	Medication management	3	Written communication	Ad hoc
Ludman 2007a	Doctor	Non-mental health practitioner	Medication management	3	Multiple methods	Scheduled
Ludman 2007b	Doctor	Non-mental health practitioner	Both	15	Multiple methods	Scheduled
Ludman 2007c	Doctor	Non-mental health practitioner	Both	25	Multiple methods	Scheduled
Mann 1998	Doctor	Non-mental health practitioner	Medication management	Not reported	Multiple methods	Ad hoc
McCusker 2008	Doctor	Mental health practitioner	Psychological therapy	5	Written communication	Ad hoc
McMahon 2007	Doctor	Mental health practitioner	Medication management	6	Liaison method	Scheduled
Menchetti 2013	Doctor	Non-mental health practitioner	Both	Not reported	Verbal	Scheduled
Morgan 2013	Doctor	Non-mental health practitioner	Both	2	Written communication	Ad hoc
Oslin 2003	Doctor	Mental health practitioner	Medication management	8	Liaison method	Scheduled
Patel 2010	Doctor	Non-mental health practitioner	Both	Not reported	Liaison method	Ad hoc
Piette 2011	Doctor	Mental health practitioner	Psychological therapy	15	Multiple methods	Scheduled
Pyne 2011	Specialist	Non-mental health practitioner	Medication management	Not reported	Shared note system	Scheduled
Richards 2008a	Doctor	Mental health practitioner	Both	10	Multiple methods	Scheduled
Richards 2008b	Doctor	Mental health practitioner	Both	10	Multiple methods	Scheduled
Richards 2012	Specialist	Mental health practitioner	Both	12	Liaison method	Scheduled
Rojas 2007	Doctor	Non-mental health practitioner	Both	8	Not reported	Ad hoc
Rollman 2009	Doctor	Non-mental health practitioner	Both	10	Multiple methods	Scheduled
Ross 2008	Doctor	Mental health practitioner	Medication management	5	Liaison method	Ad hoc
Rost 2002a	Doctor	Non-mental health practitioner	Medication management	6	Written communication	Ad hoc
Rost 2002b	Doctor	Non-mental health practitioner	Medication management	6	Written communication	Ad hoc
Rubenstein 2006	Doctor	Non-mental health practitioner	Medication management		Other	Ad hoc
Simon 2000a	Doctor	Mental health practitioner	Medication management	3	Multiple methods	Scheduled
Simon 2000b	Doctor	Mental health practitioner	Medication management	3	Multiple methods	Scheduled
Simon 2004a	Doctor	Mental health practitioner	Both	3	Multiple methods	Scheduled
Simon 2004b	Doctor	Mental health practitioner	Both	11	Multiple methods	Scheduled
Simon 2011	Doctor	Mental health practitioner	Medication management	4	Shared note system	Scheduled
Smit 2005a	Doctor	Mental health practitioner	Both	4	Written communication	Ad hoc
Smit 2005b	Doctor	Mental health practitioner	Both	4	Written communication	Ad hoc
Smit 2005c	Doctor	Mental health practitioner	Both	8	Written communication	Ad hoc
Strong 2008	Doctor	Non-mental health practitioner	Both	13	Multiple methods	Scheduled
Swindle 2003	Doctor	Mental health practitioner	Medication management	4	Liaison method	Ad hoc
Uebelacker 2011	Doctor	Non-mental health practitioner	Both	8	Written communication	Scheduled
Unutzer 2002	Doctor	Mental health practitioner	Both	15	Shared note system	Scheduled
Vera 2010	Doctor	Mental health practitioner	Medication management	Not reported	Liaison method	Scheduled
Vlasveld 2011	Specialist	Non-mental health practitioner	Both	9	Liaison method	Ad hoc
Wells 2000a	Doctor	Non-mental health practitioner	Medication management	8	Written communication	Ad hoc
Wells 2000b	Doctor	Mental health practitioner	Psychological therapy	Not reported	Written communication	Ad hoc
Wilkinson 2003	Doctor	Non-mental health practitioner	Medication management	5	Not reported	Ad hoc
Williams 2007	Mixed	Non-mental health practitioner	Medication management	7	Liaison method	Scheduled
Yeung 2010	Doctor	Non-mental health practitioner	Medication management	8	Other	Scheduled

CM  =  case manager; PCP  =  primary care provider.

### Meta-analysis of the effect of collaborative care on depressive symptoms and anti-depressant use

Compared with usual care, collaborative care was associated with improvements in depressive symptoms (standardised mean difference, SMD −0.28, 95% CI −0.33 to −0.23; I^2^ = 62.2%, 95% CI 52.2% to 70.1%; Figure S1) and increased anti-depressant use (relative risk, RR 1.53, 95% CI 1.40 to1.68; I^2^ = 80.8%, 95% CI 75.8% to 84.8%; Figure S2).

### Meta-regression model one: predictors of depressive symptoms

The results of the univariable analyses of study-level and patient-aggregate covariates on depressive symptoms are shown in Table 4 and the multivariable analyses are shown in Table 5. Four covariates were identified for inclusion in the multivariable meta-regression model: recruitment by systematic identification using either interviews, outcome measures or electronic medical records (study-level; ß −0.16, 95% CI −0.30 to −0.02), people with a chronic physical condition as an inclusion criterion (study-level; ß −0.17, 95% CI −0.28 to −0.05), psychological interventions, alone or with medication (study-level; ß −0.13, 95% CI −0.23 to −0.03), and scheduled supervision (study-level; ß −0.12, 95% CI −0.23 to −0.02). In the multivariable model only psychological intervention remained as a statistically significant predictor of depressive symptoms (study-level; ß −0.11, 95% CI −0.20 to −0.01). Studies that included psychological interventions (alone or with medication) reported greater improvements in depressive outcomes, compared with those studies that included medication management alone.

**Table 4:** Univariable predictors on depressive symptoms (N = 84).

Variable		Regression Coefficient (95% CI)	SE	P	I^2^ (95% CI)
*Dichotomous or categorical*					
Country	Non-US (vs US)	−0.04 (−0.16 to 0.07)	.06	.47	62.4 (52.5 to 70.3)
Recruitment method	Systematic (vs GP referral)	−0.16 (−0.30 to −0.02)	.07	.03	60.8 (50.3 to 69.1)
Patient sample	Medication inclusion criteria (vs not inclusion criteria)	0.08 (−0.04 to 0.20)	.06	.21	62.3 (52.3 to 70.2)
Chronic physical health condition	Present (vs absent)	−0.17 (−0.28 to −0.05)	.06	.01	58.9 (47.7 to 67.7)
Case manager background	Mental health (vs non-mental health)	0.03 (−0.07 to 0.14)	.05	.53	62.5 (52.6 to 70.3)
Intervention content	Psychological intervention or both (vs medication only)	−0.13 (−0.23 to −0.03)	.05	.01	56.8 (44.9 to 66.1)
Supervision frequency	Scheduled (vs ad hoc)	−0.12 (−0.23 to −0.02)	.05	.02	53.7 (40.7 to 63.9)
	Not applicable (vs ad hoc)	0.10 (−0.13 to 0.33)	.12	.38	
Allocation concealment	High risk (vs low risk)	0.06 (−0.03 to 0.16)	.05	.20	62.0 (51.9 to 70.0)
*Continuous*					
Enhanced usual care* ¶		0.02 (−0.03 to 0.08)	.03	.37	62.0 (51.9 to 70.0)
Number of sessions* ¶		−0.01 (−0.02 to 0.00)	.01	.26	50.7 (35.5 to 62.3)

*N of 74 comparisons.

¶ model intercepts (constants) not reported.

**Table 5:** Multivariable predictors of depressive symptoms (N = 84).

Variable	Regression Coefficient (95% CI)	SE	P
Recruitment method (Systematic)	−0.12 (−0.26 to 0.02)	.07	.10
Chronic physical health condition (Present)	−0.10 (−0.22 to 0.02)	.06	.11
Intervention content (Psychological intervention or both)	−0.11 (−0.20 to −0.01)	.05	.03
Supervision frequency (Scheduled)*	−0.08 (−0.19 to 0.02)	.05	.13
Supervision frequency (Not applicable)*	0.06 (−0.15 to 0.28)	.11	.57
Intercept (constant)	−0.05 (−0.20 to 0.10)	.08	.53

I^2^ = 47.8% (95% CI 32.6 to 59.6).

*Compared with the reference category, ad hoc supervision.

The beta coefficient reported for the multivariable predictors of depressive symptoms can be back-transformed from an SMD to a mean difference under certain assumptions for the variance of the effect [30]. We only proceeded to back-transform to the patient health questionnaire-9, for which we observed similar within variability across studies reporting on the same scale, and the beta was equivalent to a decrease of 0.67 (95% CI −1.23 to 0.06). The multivariable model reduced the I^2^ statistic from 62.2% (95% CI 52.2% to 70.1%) to 47.8% (95% CI 32.6 to 59.6).

### Meta-regression model two: predictors of anti-depressant use

The results of the univariable analyses of study level covariates on antidepressant medication use are shown in Table 6 and multivariable analyses are shown in Table 7. Two study level covariates were identified for inclusion in the multivariable meta-regression model: studies that recruited participants using systematic identification (RR 1.55, 95% CI 1.22 to 1.97) and studies that included participants with a chronic physical condition (RR 1.45, 95% CI 1.16 to 1.83). In the multivariable meta-regression model studies that recruited participants systematically (RR 1.43, 95% CI 1.12 to 1.81) and included participants with a chronic physical condition (RR 1.32, 95% CI 1.05 to 1.65) remained statistically significant predictors of anti-depressant use. Studies including participants who were systematically identified and those with a chronic physical condition adhered more to their anti-depressant medications. The multivariable model reduced the I^2^ statistic from 80.8% (95% CI 75.8% to 84.8%) to 75.2% (95% CI 68.1% to 80.7%).

**Table 6:** Univariable predictors of antidepressant use (N = 59).

Variable		Relative risk (95% CI)	SE	P	I^2^ (95% CI)
*Dichotomous or categorical*					
Country	Non-US (vs US)	0.89 (0.71 to 1.13)	.11	.34	80.1 (74.8 to 84.3)
Recruitment method	Systematic (vs GP referral)	1.55 (1.22 to 1.97)	.19	<.001	77.3 (71.0 to 82.2)
Patient sample	Medication inclusion criteria (vs not inclusion criteria)	0.85 (0.68 to 1.07)	.10	.16	80.6 (75.5 to 84.6)
Chronic physical health condition	Present (vs absent|)	1.45 (1.16 to 1.83)	.17	.001	78.2 (72.2 to 82.9)
Case manager background	Mental health (vs non-mental health)	1.06 (0.85 to 1.31)	.12	.61	80.9 (75.9 to 84.9)
Intervention content	Psychological intervention or both (vs medication only)	0.92 (0.74 to 1.14)	.10	.44	81.1 (76.2 to 85.0)
Supervision frequency	Scheduled (vs ad hoc)	1.03 (0.82 to 1.29)	.12	.83	81.3 (76.4 to 85.2)
	Not applicable (vs ad hoc)	0.94 (0.44 to 2.02)	.37	.87	
Allocation concealment	High risk (vs low risk)	0.89 (0.72 to 1.09)	.09	.25	80.9 (75.9 to 84.9)
*Continuous*					
Enhanced usual care		0.93 (0.85 to 1.03)	.05	.17	81.0 (76.0 to 84.9)
Number of sessions* ¶		0.99 (0.97 to 1.01)	.01	.54	78.2 (71.9 to 83.1)

*N of 53 comparisons.

¶ model intercepts (constants) not reported.

**Table 7:** Multivariable predictors of antidepressant use (N = 59).

Variable	Relative risk (95% CI)	SE	P
Recruitment method (systematic)	1.42 (1.12 to 1.81)	.17	.004
Chronic physical health condition (Present)*	1.32 (1.05 to 1.65)	.15	.02
Intercept (constant)	1.08 (0.88 to 1.32)	.11	.46

I^2^ = 75.2% (95% CI 68.1% to 80.7%).

*Compared with the reference category, physical health condition absent.

Sensitivity analyses using intraclass correlation coefficients of 0.00 and 0.05 for cluster trials did not impact greatly on the multivariable meta-regression findings (see Results S2 and Results S3). The subgroup analysis exploring the relationship between sample size and effect size for both depressive symptoms and medication use was statistically non-significant, thus decreasing the likelihood that our findings are susceptible to publication bias (Results S4).

### Meta-regression model three: the effect of change in anti-depressant use on depressive symptoms

Increased anti-depressant use was not associated with improvement in depressive symptoms (ß −0.13, 95% CI −0.27 to 0.004, p = 0.06).

## Discussion

Overall, collaborative care successfully improves both patient outcomes and the process of care for depression. Studies that included psychological interventions, (alone or with medication management), as part of collaborative care were associated with greater improvements in depressive symptoms compared with studies that only included medication management alone. Use of antidepressants was increased in studies that included participants with a chronic physical health condition and in studies that recruited participants through a process of systematic identification.

### Strengths and limitations

Our analyses were based on a priori decisions about covariates likely to moderate the treatment effect of collaborative care [11] and included the largest and most comprehensive dataset about collaborative care. By searching extensively, we were able to include almost twice as many trials as previous reviews, and this substantially enhanced our ability to quantify and explore heterogeneity with a greater level of statistical power, thus reducing the chance of spurious findings [31]. In addition, the large number of studies gives us more confidence in the asymptotic meta-analysis methods employed [32], even if the study effects are not normally distributed [33]. The high levels of estimated heterogeneity are a positive finding since it appears heterogeneity levels are being consistently underestimated in meta-analyses [34]. Although there is a link between meta-analysis size and heterogeneity levels and we would expect to detect high levels given the size of our review [34], as we do, the large between-study variability implies that there might be other study or patient-level variables that could explain some of it (e.g. depression severity, ethnicity of patients, fidelity to intervention, quality of case manager training, and level of engagement in psychological treatment).

Additionally, meta-regression can be weakened by other statistical considerations and poor reporting. For example, we were not able to include demographic variables in the regression models due to a lack of variability, and we were unable to model a dose response relationship between treatment effects and case management sessions because most trials did not report data about the frequency, intensity, and duration of psychological treatments. We contacted authors for this information but this process did not overcome this limitation pointing to the need for more comprehensive reporting about the delivery of treatments in psychological therapy trials. More severely depressed patients are more responsive to psychological [35] and pharmacological interventions [36] but we were unable to replicate these analyses in trials of collaborative care because depression severity was inconsistently reported; contacting authors did not overcome this issue. Failure to include these covariates may have biased our results [37]. This limitation also highlights the need for more consistent and comprehensive reporting about the content of complex mental health interventions and efforts to strengthen reporting and specification of complex behaviour change are a step forward to overcoming this limitation [38].

In the absence of individual patient data we had to rely on analyses of mean study effects which are prone to bias (‘ecological fallacy’: deducing for an individual from a group mean), which are difficult to interpret since the relationships within- and between-studies might differ, making evidence from such analyses inconclusive [27]. In addition, we could not use a single structural equation model to properly account for mediating and moderating effects and the relationships between antidepressant use and depressive symptoms.

Most trials included in this review only reported short-term follow-up limiting opportunities to conduct sensitivity analyses of moderators of long term effectiveness of collaborative care. The absence of long term follow-up data among the group of trials included in this review resembles the findings of Deshauer et al., who screened more than 2000 records for classic placebo-controlled RCTs of selective serotonin reuptake inhibitors and identified only six studies with follow-up of 6 months or greater [39]. There is a clear need to build longer term follow-up into trials of treatment of depression given that depression is episodic and the ultimate goal of treatment is to bring about sustained recovery. Additionally, most trials did not report if the trial population included patients with comorbid chronic physical health conditions, further reducing the number of comparisons entered into the regression models. Furthermore, it is reasonable to assume that all studies that included older adults will have included patients with unreported comorbidity, potentially rendering comparisons of collaborative care in chronic physical health conditions redundant. However, our analysis suggests that presence of a chronic physical health condition as a study inclusion criteria remained a key moderator of medication adherence irrespective of the presence of unreported comorbidities, suggesting that this issue has substantive significance, and does not represent confounding.

We analysed ten theoretically plausible covariates that might determine the effectiveness of collaborative care on both depressive symptoms and medication use using an initial p value of 0.10. Assuming statistical independence then approximately one in ten of our tests was susceptible to type I error but we did not adjust for multiple testing. However, we felt justified in this approach. Although adopting a more conservative approach to hypothesis testing using multiplicity adjustments would have decreased our chances of making a Type I error, we would have also increased our chances of Type II errors (i.e. false negatives), and thus rejected important predictors of the effectiveness of collaborative care outcomes [40].

### Comparisons with the previous meta-regression analysis

A novel finding of our meta-regression is that including psychological therapy, either alone or with antidepressant medication, conferred additional benefit, at least for depressive symptoms. Compared with previous analyses [11] we included a greater number of trials (+50) in our analysis which means that our analysis is the first to be powered to detect this finding. In addition, we showed that studies that systematically identified patients were important moderators of anti-depressant medication use. This finding may also be due to increased statistical power generated by our meta-regression. Additionally, we also showed that the presence of a chronic physical condition moderated use of anti-depressant medications – trials that included patients with chronic physical conditions reported increased use of anti-depressant medication. This patient characteristic was not explored in the previous meta-regression.

Contrary to the findings of a previous meta-regression [11], case-manager background did not predict reduction in depressive symptoms. Despite improvements there are still shortages of psychologists and psychotherapists, especially in low and middle-income countries [41], but case management of depression in the context of collaborative care might not need be delivered by a mental health professional. Indeed there is emerging evidence, mainly from the United States, that nurse-led collaborative care is more effective than usual care for treating depression in people with chronic physical disease [42], suggesting that non-mental health trained primary care nurses are as well placed as mental health professionals to work as case managers for certain types of patients. However, where case managers are drawn from non-mental health professions there may need to be more emphasis on ensuring that there are satisfactory arrangements in place for regular specialist supervision.

Clinical supervision of psychological therapists can positively affect the process of treatment, leading to greater confidence, self-awareness, and competence among therapists [43], and, in the context of brief psychological treatment, possibly improved patient outcomes [44]. Our initial univariable analysis adds weight to previous findings [11] that compared with ad hoc supervision the availability of scheduled case manager supervision from a mental health specialist predicted improved depressive symptom outcomes. This finding has important implications for patient benefit given that large scale epidemiological studies have shown that depression is under-treated because of inadequate anti-depressant medication management by health care providers, along with poor clinical supervision and patient follow-up [45]. Inadequate anti-depressant treatment of depression is more pronounced in people with chronic physical conditions [46]. Recurrence of symptoms is also common, but patients who continue treatment with anti-depressants reduce the risk of recurrence by 70% compared with those who discontinue treatment [47], although it is not clear whether reduction in relapse represents true long term efficacy or avoidance of relapse precipitated by antidepressant withdrawal [48]. Additionally, few patients respond to an initial 20 mg dose of citalopram and only about 40% of patients achieve remission after receiving the full therapeutic dosage of an anti-depressant medication [49]. Furthermore, up to 20% of patients remain depressed after completing an initial phase of treatment [50]. Regular supervision within collaborative care could thus help to overcome therapeutic impasse by supporting case managers to identify and manage patients who do not initially respond to or discontinue treatment by facilitating changes to anti-depressant dosage, augmentation of medication with another therapy, or recommending switching to another treatment.

### Implications for policy and practice

Our findings show that structured management plans that included psychological interventions either as a standalone therapy or in combination with antidepressant medication predicted reductions in depressive symptoms more so than collaborative care that only offered patients anti-depressant medication. While the additional effects associated with psychological treatment were small this result does highlight the importance of patient choice in the delivery of health care for depression. Across a diverse range of psychiatric conditions and health care settings patients have reported a 3-fold preference for psychological treatment over pharmacological treatment, underscoring the need to link treatment strategies to patient preference [51].

We showed that certain types of patients appear to be better at taking anti-depressant medications than others. In the absence of collaborative care depression is under-treated with anti-depressant medication in older adults with chronic physical conditions [46]. However, our findings show that this patient group showed improved levels of anti-depressant use compared with those without chronic physical conditions. It may be that patients with depression and chronic physical conditions are primed to respond well to structured management programmes that include anti-depressant medication because they are well versed in using medications to self-manage their chronic illness. However the burden of treatment for patients with complex, chronic physical and mental comorbidities may reduce their capacity to collaborate in their care [52].

Novel to this review was our finding that compared with recruitment by clinicians, trials that systematically identified patients were associated with increased use of anti-depressant medication. This finding goes beyond methodological considerations about how to effectively recruit patients into mental health trials. Patients referred to mental health services by clinicians tend to be patients with most to gain as they may be more severely depressed. However, the fact that we found that systems based approaches to patient identification predicted increased use of anti-depressant medication highlights the fact that population approaches to disease management can identify different types of patients who may have additional capacity to benefit, further underlining the importance of structured approaches to depression care.

### Future work and conclusion

Given the premise that collaborative care is an organisational framework within which different combinations of psychological and pharmacological interventions can be used a key unanswered question relates to how interventions can be tailored, adjusted, or changed to meet the needs of patients. Most collaborative care trials have only measured adherence to anti-depressants but not frequency of changes to medication dosage or augmentation of medication with another therapy. Given that use of anti-depressant medication might be a proxy for why collaborative care is effective it is critical that future trials include process measures that can evaluate adjustment and augmentation of medication similar to those used in the Teamcare trial [53]. Additionally, meta-analysis using individual rather than study-level data would increase opportunities to detect differential treatment effects across individuals in randomised trials, and allows for more complex modelling of the association of treatment effects and patient characteristics [54].

In conclusion, these results update and expand on a previous analysis of factors that differentiate collaborative care trials that improve patient outcomes and/or the process of care from those that do not. Psychological therapy is an active ingredient in collaborative care with or without anti-depressant medication, emphasising the importance of building flexible collaborative care models that include different combinations of pharmacological and non-pharmacological therapies that meet patients' treatment preferences. Furthermore, patients systematically identified and those with chronic physical conditions are likely to adhere more to pharmacological treatments. Using systems based approaches to identify patients with depression highlights the importance of borrowing elements from chronic disease management models to improve the process of depression care.

## Supporting Information

Figure S1 **Forest plot of effect of collaborative care on depressive symptoms.** *Meta-analysis of individual trial and pooled effects. Random effects model used. 95% CI = 95% confidence intervals*.
(TIFF)

Figure S2 **Forest plot of effect of collaborative care on antidepressant use.** *Meta-analysis of individual trial and pooled effects. Random effects model used. 95% CI = 95% confidence intervals*.
(TIFF)

Table S1 **Characteristics of included studies.**
(DOCX)

Methods S1 **Central Search Strategy.**
(DOCX)

Methods S2 **Meta-regression coding manual.**
(DOCX)

Checklist S1 **PRISMA Checklist.**
(DOCX)

Results S1 **Reference list of included studies.**
(DOCX)

Results S2 **Sensitivity analysis: Cluster Intraclass correlation coefficient of 0.00.**
(DOCX)

Results S3 **Sensitivity analysis: Cluster Intraclass correlation coefficient of 0.05.**
(DOCX)

Results S4 **Sensitivity analysis: Relationship between sample size and effect size.**
(DOCX)
