# Supplementary figures and images for: Characteristics of Effective Collaborative Care for Treatment of Depression: A Systematic Review and Meta-Regression of 74 Randomised Controlled Trials
Source: PLoS One. 2014 Sep 29;9(9):e108114. doi: 10.1371/journal.pone.0108114 (PMC4180075; doi:10.1371/journal.pone.0108114)

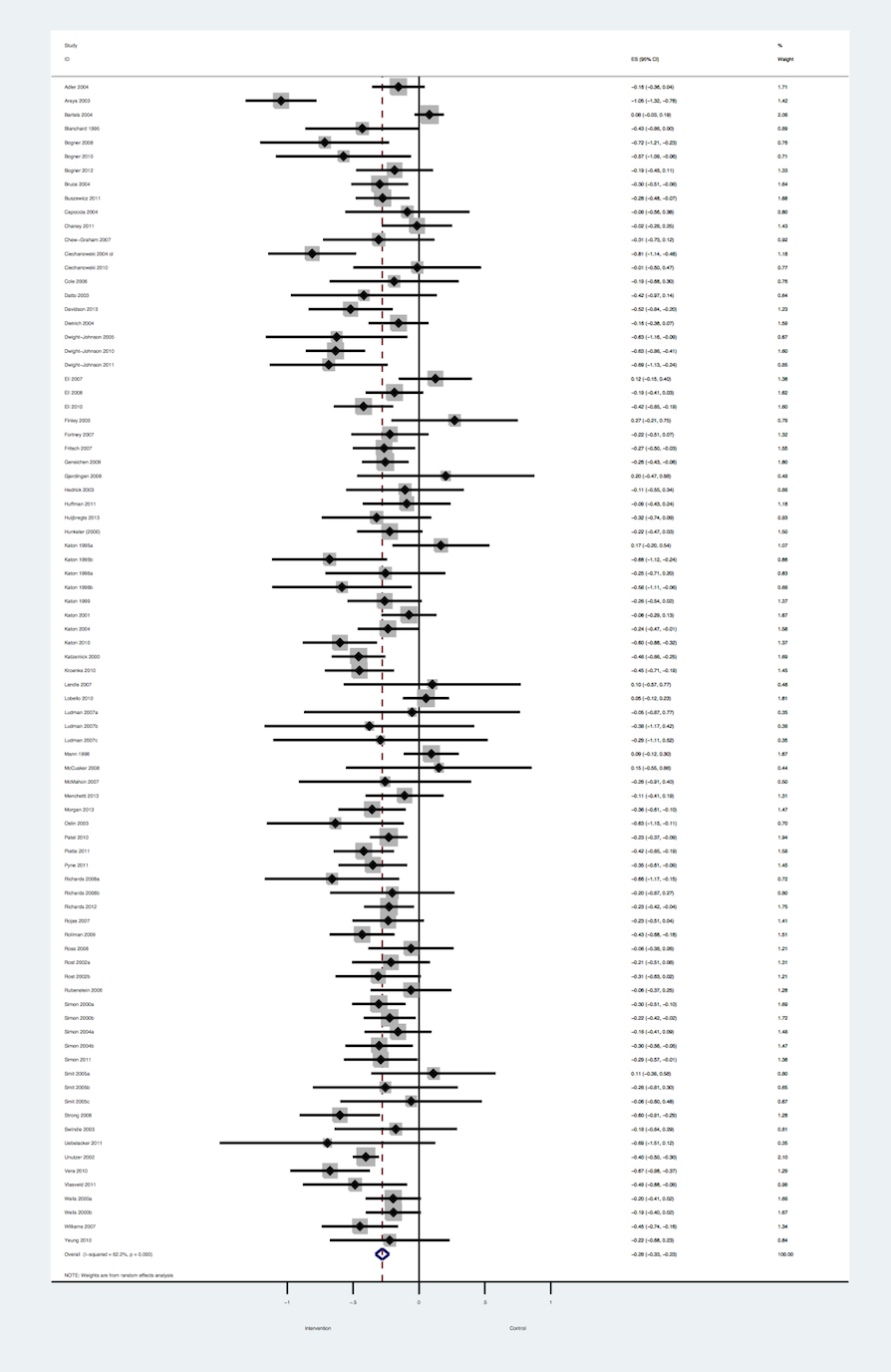

Supplement: Figure S1 — Forest plot of effect of collaborative care on depressive symptoms. Meta-analysis of individual trial and pooled effects. Random effects model used. 95% CI = 95% confidence intervals. (TIFF) [file pone.0108114.s001.tiff]

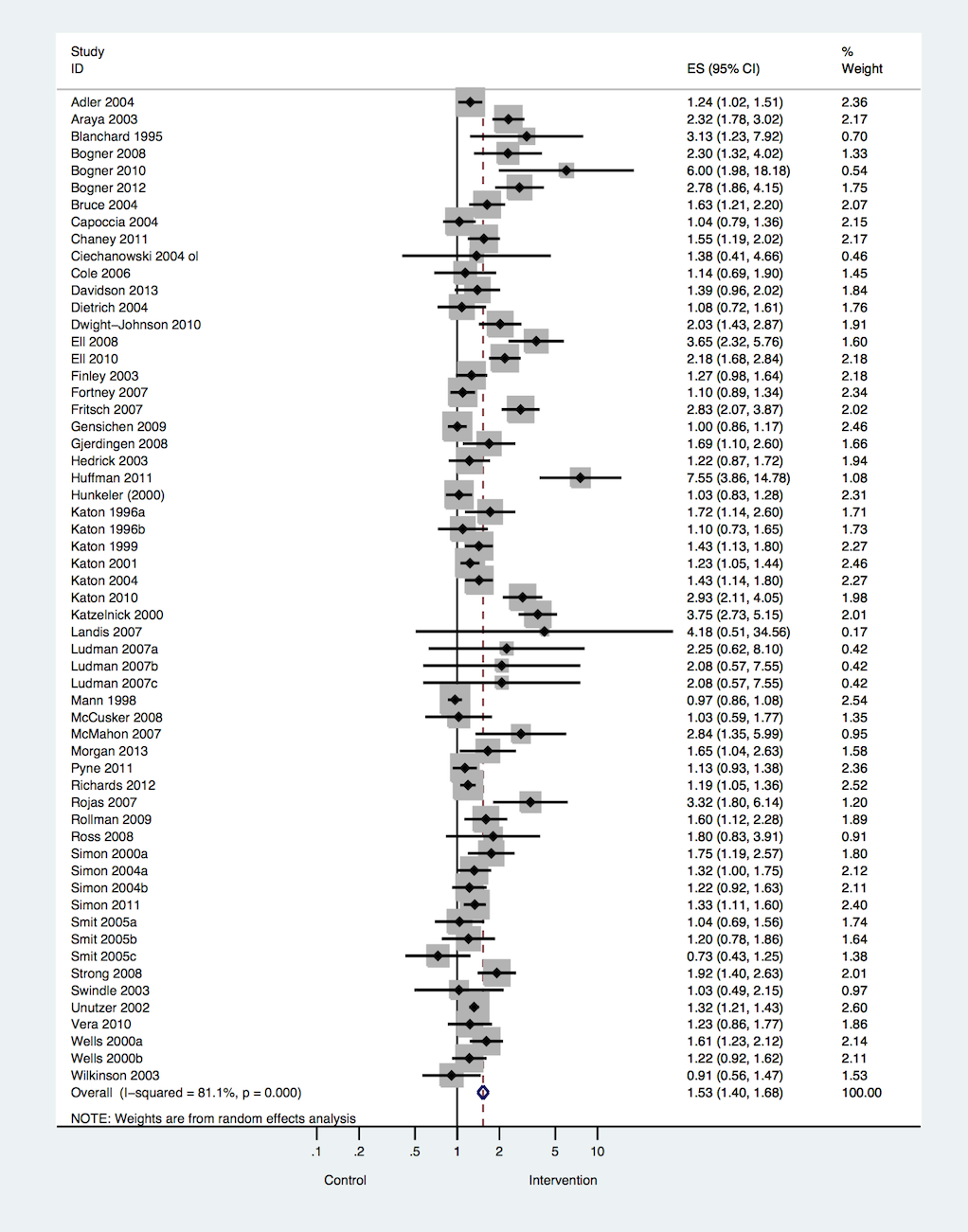

Supplement: Figure S2 — Forest plot of effect of collaborative care on antidepressant use. Meta-analysis of individual trial and pooled effects. Random effects model used. 95% CI = 95% confidence intervals. (TIFF) [file pone.0108114.s002.tiff]
