# Supplementary material for: Characteristics of Effective Collaborative Care for Treatment of Depression: A Systematic Review and Meta-Regression of 74 Randomised Controlled Trials
Source: PLoS One. 2014 Sep 29;9(9):e108114. doi: 10.1371/journal.pone.0108114 (PMC4180075; doi:10.1371/journal.pone.0108114)
Supplement: Table S1 — Characteristics of included studies. (DOCX) [file pone.0108114.s003.docx]

**Table S1. Characteristics of included studies**

| **Study** | **Baseline reported N (% female)** | **Country** | **Mean age** | **Ethnicity (%white)** | **Method of depression diagnosis** | **Severity of depression** | **Depressive outcome recorded?** | **Anti-depressant use recorded?** |
| --- | --- | --- | --- | --- | --- | --- | --- | --- |
| Adler 2004 | 507 (72) | US | 42.3 | 72.4 | Diagnostic criteria | All | Yes | Yes |
| Araya 2003 | 240 (100) | Chile | 42.6 | 0 | Diagnostic criteria | Major | Yes | Yes |
| Bartels 2004 | 1531 (31) | US | 73.9 | 45.1 | Outcome measure | All | Yes | No |
| Blanchard 1995 | 96 (85) | UK | 76.3 | NR | Diagnostic criteria | All | Yes | Yes |
| Bognor 2008 | 64 (77) | US | 58.6 | 17.2 | Practitioner diagnosis | All | Yes | Yes |
| Bognor 2010 | 58 (85) | US | 60.2 | 0 | Practitioner diagnosis | All | Yes | Yes |
| Bruce 2004 | 598 (72) | US | ≥60+ | 71.6 | Diagnostic criteria | All | Yes | Yes |
| Buszewicz 2010 | 558 (75) | UK | 48.3 | 88.2 | Diagnostic criteria | All | Yes | No |
| Capoccia 2004 | 74 (77) | US | 38.7 | 78.0 | Diagnostic criteria | Major | Yes | Yes |
| Chaney 2011 | 546(4) | US | 64.2 | 87.1 | Outcome measure | All | Yes | Yes |
| Chew-Graham 2007 | 105 (72) | UK | 75.5 | NR | Outcome measure | All | Yes | No |
| Ciechanowski 2004 | 138 (79) | US | 73.0 | 58 | Diagnostic criteria | Subthreshold | Yes | Yes |

**Table S1. Characteristics of included studies (continued)**

| **Study** | **Baseline reported N (% female)** | **Country** | **Mean age** | **Ethnicity (%white)** | **Method of depression diagnosis** | **Severity of depression** | **Depressive outcome recorded?** | **Anti-depressant use recorded?** |
| --- | --- | --- | --- | --- | --- | --- | --- | --- |
| Ciechanowski 2010 | 80 (53) | US | 43.9 | 72.5 | Outcome measure | All | Yes | Yes |
| Cole 2006 | 157 (69) | Canada | 78.0 | NR | Diagnostic criteria | Major | Yes | Yes |
| Datto 2003 | 61 (61) | US | 47.6 | 80 | Practitioner diagnosis | All | Yes | No |
| Dietrich 2004 | 405 (80) | US | 42.0 | 83.2 | Diagnostic criteria | All | Yes | Yes |
| Dwight-Johnson 2005 | 55 (100) | US | 47.3 | 0 | Outcome measure | All | Yes | No |
| Dwight-Johnson 2010 | 339 (84) | US | 49.8 | 0 | Outcome  measure | All | Yes | Yes |
| Dwight-Johnson 2011 | 101 (78) | US | 39.8 | NR | Outcome measure | All | Yes | No |
| Ell 2007 | 311 (72) | US | ≥65 | NR | Outcome measure | All | Yes | Yes |
| Ell 2008 | 472 (79) | US | ≥18 | NR | Outcome measure | All | Yes | Yes |
| Ell 2010 | 387 (82) | US | ≥18 | NR | Outcome measure | All | Yes | Yes |
| Finley 2003 | 125 (85) | US | 54.3 | NR | Practitioner diagnosis | All | Yes | Yes |
| Fortney 2007 | 395 (8) | US | 59.2 | 74.7 | Outcome measure | All | Yes | Yes |

**Table S1. Characteristics of included studies (continued)**

| **Study** | **Baseline reported N (% female)** | **Country** | **Mean age** | **Ethnicity (%white)** | **Method of depression diagnosis** | **Severity of depression** | **Depressive outcome recorded?** | **Anti-depressant use recorded?** |
| --- | --- | --- | --- | --- | --- | --- | --- | --- |
| Fritsch 2007 | 345 (100) | Chile | 37.4 | 0 | Diagnostic criteria | Major | Yes | Yes |
| Gensichen 2009 | 535 (79) | Germany | 51.1 | NR | Diagnostic criteria | Major | Yes | Yes |
| Gjerdingen 2009 | 39 (100) | US | 27.6 | 61.5 | Diagnostic criteria | Major | Yes | Yes |
| Hedrick 2003 | 354 (5) | US | 57.2 | 79.7 | Diagnostic criteria | All | Yes | Yes |
| Huffman 2011 | 175 (49) | US | 62.3 | 91.4 | Outcome measure | All | Yes | Yes |
| Hunkeler 2000 | 302 (70) | US | 55.4 | 63.0 | Practitioner diagnosis | All | Yes | Yes |
| Katon 1995a | 126 (72) | US | 51.1 | NR | Outcome measure | Subthreshold | Yes | No |
| Katon 1995b | 91 (82) | US | 42.8 | NR | Outcome measure | Major | Yes | No |
| Katon 1996a | 88 (73) | US | 48.2 | 88.6 | Outcome measure | Subthreshold | Yes | Yes |
| Katon 1996b | 65 (75) | US | 44.0 | 84.6 | Outcome measure | Major | Yes | Yes |
| Katon 1999 | 228 (75) | US | 47.0 | 80.3 | Diagnostic criteria | All | Yes | Yes |

**Table S1. Characteristics of included studies (continued)**

| **Study** | **Baseline reported N (% female)** | **Country** | **Mean age** | **Ethnicity (%white)** | **Method of depression diagnosis** | **Severity of depression** | **Depressive outcome recorded?** | **Anti-depressant use recorded?** |
| --- | --- | --- | --- | --- | --- | --- | --- | --- |
| Katon 2001 | 386 (74) | US | 46.0 | 90.2 | Practitioner diagnosis | Subthreshold | Yes | Yes |
| Katon 2004 | 329 (65) | US | 58.3 | 78.5 | Outcome measure | All | Yes | Yes |
| Katon 2010 | 214 (52) | US | 56.8 | 78.5 | Outcome measure | All | Yes | Yes |
| Katzelnick 2000 | 407 (77) | US | 45.5 | 82.8 | Diagnostic criteria | Major | Yes | Yes |
| Kroenke 2010 | 405 (68) | US | 58.8 | 79.5 | Outcome measure | All | Yes | No |
| Landis 2007 | 45 (96) | US | 39.7 | 62.2 | Outcome measure | All | Yes | Yes |
| Lobello 2010 | 520 (73) | US | 44.5 | 87.3 | Diagnostic criteria | Major | Yes | No |
| Ludman 2007a | 52 (69) | US | 50.2 | 86.0 | Diagnostic criteria | All | Yes | Yes |
| Ludman 2007b | 52 (69) | US | 50.6 | 84.6 | Diagnostic criteria | All | Yes | Yes |
| Ludman 2007c | 52 (73) | US | 50.5 | 80.8 | Diagnostic criteria | All | Yes | Yes |
| Mann 1998 | 577 (78) | UK | 44.2 | NR | Practitioner diagnosis | All | Yes | Yes |

**Table S1. Characteristics of included studies (continued)**

| **Study** | **Baseline reported N (% female)** | **Country** | **Mean age** | **Ethnicity (%white)** | **Method of depression diagnosis** | **Severity of depression** | **Depressive outcome recorded?** | **Anti-depressant use recorded?** |
| --- | --- | --- | --- | --- | --- | --- | --- | --- |
| McCusker 2008 | 68 (66) | Canada | 73.3 | NR | Diagnostic criteria | Major | Yes | Yes |
| McMahon 2007 | 62 (NR) | UK | Range 18 to 65 | NR | Diagnostic criteria | Major | Yes | Yes |
| Oslin 2003 | 97 (4) | US | 61.6 | 49.5 | Diagnostic criteria | All | Yes | No |
| Patel 2010 | 2796 (82) | India | 46.3 | NR | Outcome measure | All | Yes | No |
| Piette 2011 | 291 (52) | US | 56.0 | 84.0 | Outcome measure | All | Yes | No |
| Pyne 2011 | 249 (3) | US | 49.8 | 38.0 | Outcome measure | All | Yes | Yes |
| Richards 2008a | 76 (78) | UK | 42.2 | 85.5 | Diagnostic criteria | Major | Yes | No |
| Richards 2008b | 79 (77) | UK | 42.8 | 89.9 | Diagnostic criteria | Major | Yes | No |
| Richards 2013 | 581(72) | UK | 44.8 | 85.0 | Diagnostic criteria | Major | Yes | No |
| Rojas 2007 | 230 (100) | Chile | 26.6 | 0 | Diagnostic criteria | Major | Yes | Yes |
| Rollman 2009 | 302 (41) | US | 64 | 90.7 | Outcome measure | All | Yes | Yes |
| Ross 2008 | 223 (7) | US | 59.2 | 43.1 | Practitioner diagnosis | All | Yes | Yes |

**Table S1. Characteristics of included studies (continued)**

| **Study** | **Baseline reported N (% female)** | **Country** | **Mean age** | **Ethnicity (%white)** | **Method of depression diagnosis** | **Severity of depression** | **Depressive outcome recorded?** | **Anti-depressant use recorded?** |
| --- | --- | --- | --- | --- | --- | --- | --- | --- |
| Rost 2002a | 268 (84) | US | 42.6 | 84.3 | Diagnostic criteria | All | Yes | No |
| Rost 2002b | 211 (84) | US | 42.6 | 84.3 | Diagnostic criteria | All | Yes | No |
| Rubenstein 2002 | 567 (59) | US | 48.2 | 76 | Diagnostic criteria | Major | Yes | No |
| Simon 2000a | 392 (72) | US | 46.5 | NR | Practitioner diagnosis | All | Yes | Yes |
| Simon 2000b | 417 (71) | US | 46.4 | NR | Practitioner diagnosis | All | Yes | No |
| Simon 2004a | 402 (75) | US | 44.5 | 80.3 | Outcome measure | All | Yes | Yes |
| Simon 2004b | 393 (76) | US | 44.4 | 77.4 | Outcome measure | All | Yes | Yes |
| Simon 2011 | 208 (72) | US | 45.5 | 84.2 | Practitioner diagnosis | All | Yes | Yes |
| Smit 2006a | 184 (65) | Netherlands | 43.2 | NR | Diagnostic criteria | All | Yes | Yes |
| Smit 2006b | 111 (67) | Netherlands | 43.1 | NR | Diagnostic criteria | All | Yes | Yes |
| Smit 2006c | 116 (61) | Netherlands | 42.9 | NR | Diagnostic criteria | All | Yes | Yes |
| Strong 2008 | 200 (71) | UK | 56.6 | NR | Diagnostic criteria | Major | Yes | Yes |

**Table S1. Characteristics of included studies (continued)**

| **Study** | **Baseline reported N (% female)** | **Country** | **Mean age** | **Ethnicity (%white)** | **Method of depression diagnosis** | **Severity of depression** | **Depressive outcome recorded?** | **Anti-depressant use recorded?** |
| --- | --- | --- | --- | --- | --- | --- | --- | --- |
| Swindle 2003 | 268 (3) | US | 56.2 | 85.5 | Diagnostic criteria | All | Yes | Yes |
| Uebelacker 2011 | 38 (95) | US | 39.1 | 0 | Diagnostic criteria | All | Yes | No |
| Unutzer 2002 | 1801 (65) | US | 71.2 | 77 | Diagnostic criteria | All | Yes | Yes |
| Vera 2010 | 179 (76) | Puerto Rico | 55.0 | NR | Outcome measure | All | Yes | Yes |
| Vlasveld 2012 | 126 (54) | Netherlands | 44.8 | NR | Diagnostic criteria | Major | Yes | No |
| Wells 2000a | 867 (71) | US | 43.7 | 57 | Diagnostic criteria | All | Yes | Yes |
| Wells 2000b | 932 (71) | US | 43.7 | 57) | Diagnostic criteria | All | Yes | Yes |
| Wilkinson 1993 | 61 (74) | UK | 46.0 | NR | Practitioner diagnosis | All | No | Yes |
| Williams 2007 | 182 (54) | US | 60.0 | 60.3 | Diagnostic criteria | All | Yes | No |
| Yeung 2010 | 100 (69) | US | 49.7 | 0 | Outcome measure | All | Yes | No |

Key: N, Number; NA, No applicable; NR, Not reported
