# Supplementary material for: Characteristics of Effective Collaborative Care for Treatment of Depression: A Systematic Review and Meta-Regression of 74 Randomised Controlled Trials
Source: PLoS One. 2014 Sep 29;9(9):e108114. doi: 10.1371/journal.pone.0108114 (PMC4180075; doi:10.1371/journal.pone.0108114)
Supplement: Methods S1 — Central Search Strategy. (DOCX) [file pone.0108114.s004.docx]

**Method S1: Central Search Strategy**

| 1 | exp Depression/ |
| --- | --- |
| 2 | exp Anxiety Disorders/ |
| 3 | exp Anxiety/ |
| 4 | exp Obsessive-Compulsive Disorder/ |
| 5 | exp Panic Disorder/ |
| 6 | exp Phobic Disorders/ |
| 7 | exp Agoraphobia/ |
| 8 | claustrophobia.ti,ab. |
| 9 | social anxiety disorders.ti,ab. |
| 10 | exp Stress Disorders, Post-Traumatic/ |
| 11 | (depression or depressive or dysthymi$).ti,ab. |
| 12 | (anxious or anxiety).ti,ab. |
| 13 | (obsessi$ or compulsi$).ti,ab. |
| 14 | panic.ti,ab. |
| 15 | phobi$.ti,ab. |
| 16 | agoraphobi$.ti,ab. |
| 17 | claustrophobi$.ti,ab. |
| 18 | social anxiety.ti,ab. |
| 19 | GAD.ti,ab. |
| 20 | (PTSD or post-trauma$ or post trauma$ or posttrauma$).ti,ab. |
| 21 | 1 or 2 or 3 or 4 or 5 or 6 or 7 or 8 or 9 or 10 or 11 or 12 or 13 or 14 or 15 or 16 or 17 or 18 or 19 or 20 |
| 22 | exp Case Management/ |
| 23 | collaboration.ti,ab. |
| 24 | exp Cooperative Behavior/ |
| 25 | exp Interinstitutional Relations/ |
| 26 | exp Interprofessional Relations/ |
| 27 | multidisciplinary care team.ti,ab. |
| 28 | exp Physician-Nurse Relations/ |
| 29 | exp Patient Compliance/ |
| 30 | exp Patient-Centered Care/ |
| 31 | exp Pharmacists/ |
| 32 | teamwork.ti,ab. |
| 33 | (collaborat$ care or collaborat$ health$ or collaborat$ work$ or collaborat$ interven$ or collaborat$ service$ or collaborat$ model$ or collaborat$ effort$ or collaborat$ manag$).ti,ab. |
| 34 | (coordinat$ care or coordinat$ health$ or coordinat$ work$ or coordinat$ interven$ or coordinat$ service$ or coordinat$ model$ or coordinat$ effort$ or coordinat$ manag$).ti,ab. |
| 35 | (co-ordinat$ care or co-ordinat$ health$ or co-ordinat$ work$ or co-ordinat$ interven$ or co-ordinat$ service$ or co-ordinat$ model$ or co-ordinat$ effort$ or co-ordinat$ manag$).ti,ab. |
| 36 | (shared care or shared health$ or shared work$ or shared interven$ or shared service$ or shared model$ or shared effort$ or shared manag$).ti,ab. |
| 37 | (integrat$ care or integrat$ health$ or integrat$ work$ or integrat$ interven$ or integrat$ service$ or integrat$ model$ or integrat$ effort$ or integrat$ manag$).ti,ab. |
| 38 | (stepped care or stepped health$ or stepped work$ or stepped interven$ or stepped service$ or stepped model$ or stepped effort$ or stepped manag$).ti,ab. |
| 39 | (systematic care or systematic health$ or systematic work$ or systematic interven$ or systematic service$ or systematic model$ or systematic effort$ or systematic manag$).ti,ab. |
| 40 | (augment$ care or augment$ health$ or augment$ communicat$).ti,ab. |
| 41 | (enhance$ care$ or enhance$ health$ or enhance$ communicat$).ti,ab. |
| 42 | (case manage$ or disease manag$ or enhanced care or managed care or multi-component or multicomponent).ti,ab. |
| 43 | (care manage$ or chronic care$ or complex intervention$ or cooperative behav$ or co-operative behav$ or joint working or pathway or interprofessional or inter-professional or interdisciplinary or inter-disciplinary or multidisciplin* or mulit-disciplin$ or multiprofession$ or multi-profession$ or transdisciplin$ or trans-disciplin$ or multifacet$ or multi-facet$ or complex intervention$ or multiple intervention$ or multi-intervention$ or organisational intervention$ or organizational intervention$ or interpersonal relation$ or inter-personal relation$ or interinstitutional relation$ or inter-insitutional relation$ or consultation liaison or algorithm$ or treatment guideline$ or treatment protocol$ or treatment delivery or treatment model or adherence or compliance or concordance or patient care team or patient care management or patient care planning or case management or managed care program$ or (healthcare adj3 delivery) or (continuity adj3 care) or professional-patient relations or interprofessional relations or inter-professional relations).ti,ab. |
| 44 | (case manage$ or disease manag$ or enhanced care or managed care or multi-component or multicomponent).ti,ab. |
| 45 | 22 or 23 or 24 or 25 or 26 or 27 or 28 or 29 or 30 or 31 or 32 or 33 or 34 or 35 or 36 or 37 or 38 or 39 or 40 or 41 or 42 or 43 or 44 |
| 46 | 21 and 45 |
| 47 | limit 46 to yr="2012 -Current" |
| 1 | exp Depression/ |
| 2 | exp Anxiety Disorders/ |
| 3 | exp Anxiety/ |
| 4 | exp Obsessive-Compulsive Disorder/ |
| 5 | exp Panic Disorder/ |
| 6 | exp Phobic Disorders/ |
| 7 | exp Agoraphobia/ |
| 8 | claustrophobia.ti,ab. |
| 9 | social anxiety disorders.ti,ab. |
| 10 | exp Stress Disorders, Post-Traumatic/ |
| 11 | (depression or depressive or dysthymi$).ti,ab. |
| 12 | (anxious or anxiety).ti,ab. |
| 13 | (obsessi$ or compulsi$).ti,ab. |
| 14 | panic.ti,ab. |
| 15 | phobi$.ti,ab. |
| 16 | agoraphobi$.ti,ab. |
| 17 | claustrophobi$.ti,ab. |
| 18 | social anxiety.ti,ab. |
| 19 | GAD.ti,ab. |
| 20 | (PTSD or post-trauma$ or post trauma$ or posttrauma$).ti,ab. |
| 21 | 1 or 2 or 3 or 4 or 5 or 6 or 7 or 8 or 9 or 10 or 11 or 12 or 13 or 14 or 15 or 16 or 17 or 18 or 19 or 20 |
| 22 | exp Case Management/ |
| 23 | collaboration.ti,ab. |
| 24 | exp Cooperative Behavior/ |
| 25 | exp Interinstitutional Relations/ |
| 26 | exp Interprofessional Relations/ |
| 27 | multidisciplinary care team.ti,ab. |
| 28 | exp Physician-Nurse Relations/ |
| 29 | exp Patient Compliance/ |
| 30 | exp Patient-Centered Care/ |
| 31 | exp Pharmacists/ |
| 32 | teamwork.ti,ab. |
| 33 | (collaborat$ care or collaborat$ health$ or collaborat$ work$ or collaborat$ interven$ or collaborat$ service$ or collaborat$ model$ or collaborat$ effort$ or collaborat$ manag$).ti,ab. |
| 34 | (coordinat$ care or coordinat$ health$ or coordinat$ work$ or coordinat$ interven$ or coordinat$ service$ or coordinat$ model$ or coordinat$ effort$ or coordinat$ manag$).ti,ab. |
| 35 | (co-ordinat$ care or co-ordinat$ health$ or co-ordinat$ work$ or co-ordinat$ interven$ or co-ordinat$ service$ or co-ordinat$ model$ or co-ordinat$ effort$ or co-ordinat$ manag$).ti,ab. |
| 36 | (shared care or shared health$ or shared work$ or shared interven$ or shared service$ or shared model$ or shared effort$ or shared manag$).ti,ab. |
| 37 | (integrat$ care or integrat$ health$ or integrat$ work$ or integrat$ interven$ or integrat$ service$ or integrat$ model$ or integrat$ effort$ or integrat$ manag$).ti,ab. |
| 38 | (stepped care or stepped health$ or stepped work$ or stepped interven$ or stepped service$ or stepped model$ or stepped effort$ or stepped manag$).ti,ab. |
| 39 | (systematic care or systematic health$ or systematic work$ or systematic interven$ or systematic service$ or systematic model$ or systematic effort$ or systematic manag$).ti,ab. |
| 40 | (augment$ care or augment$ health$ or augment$ communicat$).ti,ab. |
| 41 | (enhance$ care$ or enhance$ health$ or enhance$ communicat$).ti,ab. |
| 42 | (case manage$ or disease manag$ or enhanced care or managed care or multi-component or multicomponent).ti,ab. |
| 43 | (care manage$ or chronic care$ or complex intervention$ or cooperative behav$ or co-operative behav$ or joint working or pathway or interprofessional or inter-professional or interdisciplinary or inter-disciplinary or multidisciplin* or mulit-disciplin$ or multiprofession$ or multi-profession$ or transdisciplin$ or trans-disciplin$ or multifacet$ or multi-facet$ or complex intervention$ or multiple intervention$ or multi-intervention$ or organisational intervention$ or organizational intervention$ or interpersonal relation$ or inter-personal relation$ or interinstitutional relation$ or inter-insitutional relation$ or consultation liaison or algorithm$ or treatment guideline$ or treatment protocol$ or treatment delivery or treatment model or adherence or compliance or concordance or patient care team or patient care management or patient care planning or case management or managed care program$ or (healthcare adj3 delivery) or (continuity adj3 care) or professional-patient relations or interprofessional relations or inter-professional relations).ti,ab. |
| 44 | (case manage$ or disease manag$ or enhanced care or managed care or multi-component or multicomponent).ti,ab. |
| 45 | 22 or 23 or 24 or 25 or 26 or 27 or 28 or 29 or 30 or 31 or 32 or 33 or 34 or 35 or 36 or 37 or 38 or 39 or 40 or 41 or 42 or 43 or 44 |
| 46 | 21 and 45 |
| 47 | limit 46 to yr="2012 -Current" |
