# Supplementary material for: Characteristics of Effective Collaborative Care for Treatment of Depression: A Systematic Review and Meta-Regression of 74 Randomised Controlled Trials
Source: PLoS One. 2014 Sep 29;9(9):e108114. doi: 10.1371/journal.pone.0108114 (PMC4180075; doi:10.1371/journal.pone.0108114)
Supplement: Methods S2 — Meta-regression coding manual. (DOCX) [file pone.0108114.s005.docx]

# Methods S2. Meta-regression coding manual

## Study setting: Country

| **Binary code** |
| --- |
| 0=US |
| 1=Other |

**GUIDANCE NOTES:**

**-**Code accordingly.

## Recruitment method

| **Binary code** |
| --- |
| 0=No (Primary care provider identified/self-selected) |
| 1=Yes (Screened on outcome measure/diagnostic interview or identified from electronic medical records) |

**GUIDANCE NOTES:**

-If multiple screens were used this code applied to the primary screen only (not thresholds that must be met once inclusion/exclusion criteria applied).

## Socio-demographics: Age

| **Codes** |
| --- |
| 0=≤18 |
| 1=18-64 |
| 2=≥65 |
| 3=No restriction |
| 4=Not reported |

**GUIDANCE NOTES:**

-Code based on lower limit if no upper limit is given (e.g. participants must be 18 or above code as 1)

-Code for both inclusion/exclusion criteria and empirically reported data (extract verbatim mean and standard deviation and then apply code)

## Socio-demographics: Ethnicity

| **Codes** |
| --- |
| 0=≥75% white |
| 1=25% Other |
| 2=Not reported |

**GUIDANCE NOTES:**

**-**Code accordingly for empirically reported data.

## Socio-demographics: Gender

| **Codes** |
| --- |
| 0=≥50% female |
| 1=≤49% female |
| 2=Not reported |

**GUIDANCE NOTES:**

**-**Code accordingly for empirically reported data.

## Clinical characteristics: Anti-depressant use

| **Codes** |
| --- |
| 0=Sample does not have to be on or willing to take medication. |
| 1=Sample must be on/willing to take medication |

**GUIDANCE NOTES:**

**-**Code accordingly based on inclusion/exclusion criteria.

## Clinical characteristics: Mental health diagnostic severity

| **Codes** |
| --- |
| 0=Sub-threshold/minor depressive disorder/dysthymia |
| 1=Major depressive disorder |
| 2=Both 0 & 1 |

**GUIDANCE NOTES**:

- Based exclusively on how authors define their inclusion/exclusion criteria.

-If no distinction is made between minor and major then code as 2.

## Co-morbid chronic physical health condition

| **Codes** |
| --- |
| 0=No-participants with a chronic physical condition were not actively recruited as part of inclusion criteria |
| 1=Yes-participants with a chronic physical condition were actively recruited as part of inclusion criteria |

**GUIDANCE NOTES**:

-A chronic physical health condition is “a condition that cannot, at present, be cured but is controlled by medication and/or other treatment/therapies”[1] .

-Hypertension was included as a chronic physical health condition after consulting with GPs.

## Primary care provider (PCP): Professional background

| **Comprehensive code** | **Binary code** |
| --- | --- |
| 0=Primary care doctor (e.g. GP, family physician) | 0=Primary care provider (includes nurse) |
| 1=Nurse | 1=Specialist |
| 2=Specialist |  |
| 3=Mixed |  |

**GUIDANCE NOTES**:

-If professional group is mixed code according to majority BUT if equal proportions are reported or proportions are not reported code as mixed.

-If primary care provider reported generically assume doctor.

-When applying the binary code if there are mixed professional groups code as 0=PCP.

## Primary care provider (PCP): Training and education?

| **Code** |
| --- |
| 0=No |
| 1=Yes |
| 2=Not reported/unclear |

### GUIDANCE NOTES:

-Code for both the intervention and control group separately.

-This applies to face to face training/online didactic training e.g. trainer provides trainee with feedback.

-If provided with training materials e.g. video, this is coded under training and education materials (see later, section 12).

-If training is not explicitly described for the usual care/control group code as 2.

-If trained in **recruitment** of participants code as 0 e.g. Rost 2002

-If trained to promote **involvement** code as 0 e.g. Cappoccia, 2004

-If training differed by site code according to the type of training delivered in the majority of sites e.g. Ell 2007.

-If invited to attend conferences by research team code as 1.

## Primary care provider (PCP): Received evidence based guideline?

| **Code** |
| --- |
| 0=No |
| 1=Yes |
| 2=Not reported/unclear |

**GUIDANCE NOTES**:

-Code for both intervention and control group in appropriate column.

-Code as 1 if treatment or medication protocols/algorithms/guidelines are supplied to the PCP directly as part of study.

-Code as 1 if received training in the delivery of evidence based guidelines e.g. Genischen 2009.

-Code as 0 if training is referred to generically but with no reference to medication protocols, algorithms or guidelines e.g. Dietrich, 2004.

-Code as 0 if training materials were given but with no reference to guidelines/algorithms/protocols e.g. Asarnow 2005.

-Code as 0 if treatment or medication protocols/algorithms are supplied to the PCP via the case manager and these algorithms were tailored to each individual patient (e.g. this is case management).

-Code as 1 if stepped care model was used; if the PCP is informed of appropriate steps via case manager code as 0.

## Primary care provider (PCP): Received training and education materials?

| **Code** |
| --- |
| 0=No |
| 1=Yes |
| 2=Not reported/unclear |

**GUIDANCE NOTES**:

-Code for both intervention and control group in appropriate column. Example training materials include DVD or workbook.

## Usual care: Was it enhanced?

| **Code** |
| --- |
| 0=Standard |
| 1=Enhanced |

**GUIDANCE NOTES**:

*Usual care defined as:*

Any care that is routinely given to participants with depression and can include onward referral to mental health teams if deemed necessary by the health care provider. Health care providers could be made aware of their patients’ depressive status. Patients may be made aware of the resources available to them in the treatment setting where they are currently receiving treatment.

-A code of 1 is given if patients receive anything in addition to the above statement. Examples of types of enhanced usual care are provided in the table below.

-If informed of local resources (external to those provided by recruitment setting) code as 1.

-If informed of resources available at the clinic code as 0.

-Excludes training and education as this information is coded in Section 10.

| **Types of enhanced usual care** |
| --- |
| 1=Enhanced referral |
| 2=Case review with two or more health professionals |
| 3=Educational materials AND/OR list of locally available resources |
| 4=Consultation liaison |
| 5=Letter with self-help advice |
| 6=Equal contact time but focus on physical health |
| 7=Treatment plan mailed to primary care provider from principal investigator |
| 8=Instant messaging service with primary care provider |
| 9=Medication management |

## Enhanced usual care (EUC): ordinal scale

**GUIDANCE NOTES**:

-For sections 10, 11, and 12 recode not reported (2) as zero.

-Sum the scores for the usual care group only across sections 10, 11, 12, and 13 to generate an ordinal scale that ranges from zero to four.

## Case manager professional background

| **Code** |
| --- |
| 0=Yes mental health professional |
| 1=Non-mental health professional background |

**GUIDANCE NOTES**:

-Social workers were classed as mental health professionals.

-Research nurse coded as 1.

-If health professionals are drawn from mix of professional backgrounds then code as mental health professional unless there is evidence that mental health professionals are the minority group in the study.

-Any training and or experience in mental health has to be outside the study for a code of mental health professional to be applied.

-Counsellor coded as 1.

-If no evidence is provided default to coding as 1 e.g. ‘service co-ordinator’ or ‘nurse’.

-If just GP and psychiatrist included code the psychiatrist as the case manager.

## Intervention content

| **Code** |
| --- |
| 0=Medication management |
| 1=Psychological therapy alone or with medication management |

**GUIDANCE NOTES**:

-Where motivational interviewing was used to improve medication adherence code as 0.

-Where onward referral to psychological therapy was the role of case manager code as 0.

-Where there has been mention of activities such as exercise or social activities but not within a behavioural activation framework code as 0

-Unless it is a recognised psychotherapy code as 0

-If intervention is unclear code as 0

-If not all participants received psychological intervention but it was offered to some as part of the protocol code as 1.

-Psychoeducation code as 1

-Used cognitive behavioural therapy principles e.g. cognitive restructuring, problem solving, activity scheduling, relapse prevention code as 1

-Self-help manuals provided and/or used in intervention e.g. bibliotherapy, computerised cognitive behavioural therapy code as 1

-Counselling code as 0

-If psychotherapy is delivered for other conditions e.g. alcoholism then code as 0 e.g. Bartels 2004

## Number of case management sessions planed in the first six months

**GUIDANCE NOTES**:

-Extract verbatim for both planned and actual number received.

-Code according to minimum number of sessions offered. For example, if eight sessions were planned with an option to have six additional follow-up sessions, code as eight.

-If number of sessions varied according to treatment model calculate the mean across the groups if possible.

-If a range is reported report the highest number in range (e.g. 6-8 sessions were offered to all patients’ code as eight).

-Exclude personalised mailings/automated calls from total number of contacts e.g. Katon, 2009.

## Case management session frequency

| **Codes** |
| --- |
| 0=Not reported |
| 1=Weekly |
| 2=Fortnightly |
| 3= Monthly |
| 4=Other |

**GUIDANCE NOTES**:

-Extract for both planned and actual frequency received.

-Code to highest intensity delivered if variable frequencies were delivered.

-If no consistent frequency pattern reported code as other.

## Case management session duration

| **Comprehensive code** |
| --- |
| 0=Not reported |
| 1=0-15 mins |
| 2=16-30 mins |
| 3= 31-45 mins |
| 4=46-60 mins |
| 5=60 + mins |

**GUIDANCE NOTES**:

-Extract for both planned and actual session duration.

-If different durations were delivered per type of treatment received calculate the total mean.

-If durations differed for assessment and follow-up calculate mean.

-If only most intensive treatment mode reported e.g. psychological therapy as opposed to medication management, then code according to reported information.

## Case manager liaison with primary care provider

| **Codes** |
| --- |
| 0=Not reported |
| 1=Verbal |
| 2=Shared note system |
| 3=Written communication |
| 4= Multiple methods |
| 5=Liaison method not specified but occurred |
| 6=By psychiatrist only |
| 7=No active liaison |

**GUIDANCE NOTES:**

-If use word “discuss” code as verbal.

-Case conference code as verbal.

-Consultation code as verbal.

-If no liaison provided after initial assessment code as not reported e.g. Rost 2002.

## Specialist professional background

| **Codes** |
| --- |
| 0=Mental health specialist |
| 1=Non-mental health specialist |
| 2=Unclear (e.g. who specialist is) |
| 3=Same as case manager |

**GUIDANCE NOTES**:

-A psychiatrist, psychotherapist or psychologist was considered to be a specialist.

-If mixed professional background code according to majority.

## Specialist liaison with primary care provider

| **Codes** |
| --- |
| 0=Not reported |
| 1=Verbal |
| 2=Shared note system |
| 3=Written communication (e.g. letter/fax) |
| 4=Multiple methods |
| 5=Liaison method not specified but occurred |
| 6=None reported |
| 7=NA |
| 999=Unclear |

**GUIDANCE NOTES**:

-Code accordingly

-If use word “discuss” code as verbal

-Case conference code as verbal.

-Consultation code as verbal.

## Frequency of case management supervision

| **Codes** |
| --- |
| 1=Ad hoc/none |
| 2=Scheduled |
| 3=Not reported |
| 4=NA/same as CM |

**GUIDANCE NOTES**

-NA (not applicable) applies to those studies in which there was no specialist but the case manager had specialist mental health training (e.g. psychologist, psychiatrist, psychotherapist).

-If number of hours reported but no description of frequency code as ad hoc e.g. Araya 2003

-If ‘regular’ supervision is reported but regularity is not specified code as 1.

-If mean number of contacts were reported but it is unclear how often this occurred code as 1.

-If supervision was initially scheduled then changed to ad hoc once case manager becomes more accomplished code as 1.

-If an approximation of the frequency of contact is given, e.g. Patel 2010, code as 1.

-To collapse into three levels for meta-regression code not reported as 1. If a code of 4 was given and the case manager was not a mental health specialist then code as 1.

## Risk of bias

Use Cochrane’s risk of bias tool [2]

## Sample size

Extract total sample to perform sensitivity analyses for publication bias.

# References

**1.** Department of Health. Improving quality of life for people with long term conditions. https://www.gov.uk/government/policies/improving-quality-of-life-for-people-with-long-term-conditions. Accessed 5 August, 2013.

**2.** Higgins JP, Altman DG, Gotzsche PC, et al. The Cochrane Collaboration’s tool for assessing risk of bias in randomised trials.*BMJ*. 201; 343:d5928
