# Supplementary material for: Characteristics of Effective Collaborative Care for Treatment of Depression: A Systematic Review and Meta-Regression of 74 Randomised Controlled Trials
Source: PLoS One. 2014 Sep 29;9(9):e108114. doi: 10.1371/journal.pone.0108114 (PMC4180075; doi:10.1371/journal.pone.0108114)
Supplement: Results S1 — Reference list of included studies. (DOCX) [file pone.0108114.s007.docx]

# Results S1. Reference list of included studies

*****main trial paper

**Adler 2004 (*published data only)***

*Adler DA, Bungay KM, Wilson IB, Pei Y, Supran S, Peckham E, et al. The impact of a pharmacist intervention on 6-month outcomes in depressed primary care patients. *General Hospital Psychiatry* 2004;**26**(3):199-209.

Bungay KM, Adler DA, Rogers WH, McCoy C, Kaszuba M, Supran S, et al. Description of a clinical pharmacist intervention administered to primary care patients with depression. *General Hospital Psychiatry* 2004;**26**(3):210-8.

**Araya 2003 *(published data only)***

Araya R, Flynn T, Rojas G, Fritsch R, Simon G. Cost-effectiveness of a primary care treatment program for depression in low-income women in Santiago, Chile. *American Journal of Psychiatry* 2006;**163**(8):1379-87.

*Araya R, Rojas G, Fritsch R, Gaete J, Rojas M, Simon G, et al. Treating depression in primary care in low-income women in Santiago, Chile: a randomised controlled trial. *Lancet* 2003;**361**(9362):995-1000.

Rojas G, Araya R, Simon G, Fritsch R, Gaete J, editors. Treating depression among low income women in primary care, Santagio, Chile. 156th Annual Meeting of the American Psychiatric Association; 2003 May 17-22; San Francisco, CA: NR109.

**Bartels 2004 *(published data only)***

Areán PA, Ayalon L, Jin C, McCulloch CE, Linkins K, Chen H, et al. Integrated specialty mental health care among older minorities improves access but not outcomes: Results of the PRISMe study. *International Journal of Geriatric Psychiatry* 2008;**23**(10):1086-92.

Azar AR, Chopra MP, Cho LY, Coakley E, Rudolph JL. Remission in major depression: results from a geriatric primary care population. *International Journal of Geriatric Psychiatry* 2011;**26**(1):48-55.

*Bartels SJ, Coakley EH, Zubritsky C, Ware JH, Miles KM, Areán PA, et al. Improving access to geriatric mental health services: a randomized trial comparing treatment engagement with integrated versus enhanced referral care for depression, anxiety, and at-risk alcohol use. *American Journal of Psychiatry* 2004;**61**(8):1455-62.

Chen H, Coakley EH, Cheal K, Maxwell J, Costantino G, Krahn DD, et al. Satisfaction with mental health services in older primary care patients. *American Journal of Geriatric Psychiatry* 2006;**14**(4):371-9.

Datto CJ, Thompson R, Knott K, Katz IR. Older adult report of change in depressive symptoms as a treatment decision tool. *Journal of the American Geriatrics Society* 2006;**54**(4):627-31.

Gallo JJ, Zubritsky C, Maxwell J, Nazar M, Bogner HR, Quijano LM, et al. Primary care clinicians evaluate integrated and referral models of behavioral health care for older adults: results from a multisite effectiveness trial (PRISM-e). *The Annals of Family Medicine* 2004;**2**(4):305-9.

Krahn D, Bartels S, Coakley E, Oslin D, Chen H, McIntyre J, et al. PRISM-E: comparison of integrated care and enhanced specialty referral models in depression outcomes. *Psychiatric Services* 2006; **57**(7):946-53.

Levkoff SE, Chen H, Coakley E, Herr ECM, Oslin DW, Katz I, et al. Design and sample characteristics of the PRISM-E multisite randomized trial to improve behavioral health care for the elderly. *Journal of Aging and Health* 2004;**16**(1):3-27.

Mavandadi S, Ten Have TR, Katz IR, Durai UNB, Krahn DD, Llorente MD, et al. Effect of depression treatment on depressive symptoms in older adulthood: the moderating role of pain. *Journal of the American Geriatrics Society* 2007; **55**(2):202-11.

Wiley-Exley E, Domino ME, Maxwell J, Levkoff SE. Cost-effectiveness of integrated care for elderly depressed patients in the PRISM-E study. *The Journal of Mental Health Policy and Economics* 2009;**12**(4):205-13.

Wittink MN, Oslin D, Knott KA, Coyne JC, Gallo JJ, Zubritsky C. Personal characteristics and depression‐related attitudes of older adults and participation in stages of implementation of a multi‐site effectiveness trial (PRISM‐E). *International Journal of Geriatric Psychiatry* 2005;**20**(10):927-37.

Zanjani F, Mavandadi S, TenHave T, Katz I, Durai NB, Krahn D, et al. Longitudinal course of substance treatment benefits in older male veteran at-risk drinkers. *Journal of Gerontology*; **63**(1):98-106.

**Blanchard 1995 (*published data only)***

*Blanchard M, Waterreus A, Mann A. The effect of primary care nurse intervention upon older people screened as depressed. *International Journal of Geriatric Psychiatry* 1995;**10**(4):289-98.

Mann AH, Blanchard M, Waterreus A. Depression in older people: Some criteria for effective treatment. *L'Encéphale* 1993;**19**(Spec No 3): 445-50

Waterreus A, Blanchard M, Mann A. Community psychiatric nurses for the elderly: well tolerated, few side‐effects and effective in the treatment of depression. *Journal of Clinical Nursing* 1994;**3**(5):299-306.

**Bognor 2008 *(published data only)***

*Bogner HR, de Vries HF. Integration of depression and hypertension treatment: a pilot, randomized controlled trial.  *Annals of Family Medicine* 2008;**6**(4):295-301.

**Bognor 2010 *(published data only)***

*Bogner HR, de Vries HF. Integrating type 2 diabetes mellitus and depression treatment among African Americans a randomized controlled pilot trial. *The Diabetes Educator* 2010;**36**(2):284-92.

**Bognor 2012 (published data only)**

*Bogner HR, Morales KH, de Vries HF, Cappola AR. Integrated management of type 2 diabetes mellitus and depression treatment to improve medication adherence: a randomized controlled trial. *Annals of Family Medicine* 2012;**10**(1):15-22.

**Bruce 2004 *(published data only)***

Alexopoulos GS, Katz IR, Bruce ML, Heo M, Ten Have T, Raue P, et al. Remission in depressed geriatric primary care patients: a report from the PROSPECT study. *The American Journal of Psychiatry* 2005; **62**(4):718-24.

Alexopoulos GS, Reynolds III CF, Bruce ML, Katz IR, Raue PJ, Mulsant BH, et al. Reducing suicidal ideation and depression in older primary care patients: 24-month outcomes of the PROSPECT study. *The American Journal of Psychiatry* 2009;**166**(8):882-90.

Bao Y, Alexopoulos GS, Casalino LP, Ten Have TR, Donohue JM, Post EP, et al. Collaborative depression care management and disparities in depression treatment and outcomes. *Archives of General Psychiatry* 2011;**68**(6):627-36.

Bao Y, Casalino LP, Ettner SL, Bruce ML, Solberg LI, Unützer J. Designing payment for collaborative care for depression in primary care. *Health Services Research* 2011;**46**(5):1436-51.

Bao Y, Post EP, Ten Have TR, Schackman BR, Bruce ML. Achieving Effective Antidepressant Pharmacotherapy in Primary Care: The Role of Depression Care Management in Treating Late‐Life Depression. *Journal of the American Geriatrics Society* 2009;**57**(5):895-900.

Bogner HR, Bruce ML, Reynolds CF, Mulsant BH, Cary MS, Morales K, et al. The effects of memory, attention, and executive dysfunction on outcomes of depression in a primary care intervention trial: the PROSPECT study. *International Journal of Geriatric Psychiatry* 2007;**22**(9):922-9.

Bogner HR, Cary MS, Bruce ML, Reynolds CF, Mulsant B, Have TT, et al. The Role of Medical Comorbidity in Outcome of Major Depression in Primary Care: The PROSPECT Study. *The American Journal of Geriatric Psychiatry* 2005;**13**(10):861-8.

Bogner HR, Lin JY, Morales KH. Patterns of early adherence to the antidepressant citalopram among older primary care patients: the prospect study. *The International Journal of Psychiatry in Medicine* 2006;**36**(1):103-19.

Bogner HR, Morales KH, Post EP, Bruce ML. Diabetes, Depression, and Death A randomized controlled trial of a depression treatment program for older adults based in primary care (PROSPECT). *Diabetes Care* 2007;**30**(12):3005-10.

Bruce ML, Pearson JL. Designing an intervention to prevent suicide: PROSPECT (Prevention of suicide in primary care elderly: Collaborative trial). *Dialogues in Clinical Neuroscience* 1999;**1**(2):100-12.

*Bruce ML, Ten Have TR, Reynolds III CF, Katz II, Schulberg HC, Mulsant BH, et al. Reducing suicidal ideation and depressive symptoms in depressed older primary care patients. *JAMA* 2004;**291**(9):1081-91.

Byers AL, Bruce, ML, Raue P. Suicidal ideation in non-depressed elderly primary care patients: The PROSPECT Study (abstract). *American Journal of Geriatric Psychiatry* 2009;**17**: A86.

Gallo JJ, Bogner HR, Morales KH, Post EP, Lin JY, Bruce ML. The Effect of a Primary Care Practice–Based Depression Intervention on Mortality in Older AdultsA Randomized Trial. *Annals of Internal Medicine* 2007;**146**(10):689-98.

Joo JH, Morales KH, De Vries HF, Gallo JJ. Disparity in Use of Psychotherapy Offered in Primary Care Between Older African‐American and White Adults: Results from a Practice‐Based Depression Intervention Trial. *Journal of the American Geriatrics Society* 2010;**58**(1):154-60.

Lin JY, Ten Have TR, Bogner HR, Elliott MR. Baseline patient characteristics and mortality associated with longitudinal intervention compliance. *Statistics in Medicine* 2007;**26**(28):5100-15.

Lyness JM, Heo M, Datto CJ, Ten Have TR, Katz IR, Drayer R, et al. Outcomes of minor and subsyndromal depression among elderly patients in primary care settings. *Annals of Internal Medicine* 2006;**144**(7):496-504.

Reynolds CF, Alexopoulos GS, Katz I, Bruce M, Schulberg HC, Tenhave, T. Shifting paradigm : Methodological and ethical questions in geriatric depression treatment research in the general medical sector. 39^th^ Annual Meeting of the American College of Neuropsychopharmacology; 2000 Dec 10-14; San Juan; Puerto Rico. 2000: 65.

Reynolds CF 3rd, Degenholtz H,Parker LS, Schulberg HC, Mulsant BH, Post E, et al. Treatment as usual(TAU) control practices in the PROSPECT Study: managing the interaction and tension between research design and ethics. *International Journal of Geriatric Psychiatry* 2001;**16**(6): 602–8.

Schulberg HC,Post EP, Raue PJ,TenHave T,Miller M, Bruce ML. Treating late-life depression with interpersonal psychotherapy in the primary care sector. *International Journal of Geriatric Psychiatry* 2007;**22**(2):106–14.

Wallace ML, Dombrovski AY, Morse JQ, Houck PR, Frank E, Alexopoulos GS, et al. Coping with health stresses and remission from late-life depression in primary care: A two year prospective study. *International Journal of Geriatric Psychiatry* 2012;**27**(2):178–86.

Williams JW Jr. Managed depression care reduces mortality in older adults with major depression. *Evidence-Based Mental Health* 2008;**11**(1):16.

**Buszewicz 2010 (*published data only)***

*Buszewicz M, Griffin M, McMahon E, Beecham J, King M. Evaluation of a system of structured, pro-active care for chronic depression in primary care: a randomised controlled trial. *BMC psychiatry* 2010;**10**(1):61.

Buszewicz M, Griffin M, Beecham J, Bonin E-M, Hutson M. *ProCEED: report of a study of proactive care by practice nurses for people with depression and anxiety.* Mind: London, UK 2011.

**Capoccia 2004 (*published data only)***

Boudreau DM, Capoccia KL, Sullivan SD, Blough DK, Ellsworth AJ, Clark DL, etal. Collaborative care model to improve outcomes in major depression. *Annals of Pharmacotherapy* 2002; **36**(4):585–91.

*Capoccia KL, Boudreau DM, Blough DK, Ellsworth AJ, Clark DR, Stevens NG, et al. Randomized trial of pharmacist interventions to improve depression care and outcomes in primary care. *American Journal of Health-System Pharmacy* 2004;**61**(4):364-72.

**Chaney 2011 *(published data only)***

Chan D. Depression and comorbid PTSD in veterans: Evaluation of collaborative care programs and impact on utilization and costs[thesis]. *Dissertation Abstracts International: Section B: The Sciences and Engineering* 2008;**68** (12B):7922.

Chan D, Cheadle AD, Reiber G, Untzer J, Chaney EF. Health care utilization and its costs for depressed veterans with and without comorbid PTSD symptoms. *Psychiatric Services* 2009;**60**(12):1612–7.

*Chaney EF, Rubenstein LV, Liu C-F, Yano EM, Bolkan C, Lee M, et al. Implementing collaborative care for depression treatment in primary care: a cluster randomized evaluation of a quality improvement practice redesign. *Implementation Science* 2011;**6**(1):121 [DOI:10.1186/1748-5908-6-121]

Chaney EF. Well-being among veterans enhancement study(WAVES) [NCT00105820]. ClinicalTrials.gov [www.clinicaltrials.gov] 2005.

**Chew-Graham 2007 (*published data only)***

*Chew-Graham CA, Lovell K, Roberts C, Baldwin R, Morley M, Burns A, et al. A randomised controlled trial to test the feasibility of a collaborative care model for the management of depression in older people. *The British Journal of General Practice* 2007;**57**(538):364-70.

Gilbody S. Depression in older adults: collaborative care model seems effective. Evidence Based Mental Health 2008;**11**(2):44.

**Ciechanowski 2004 (*published data only)***

*Ciechanowski P, Wagner E, Schmaling K, Schwartz S, Williams B, Diehr P, et al. Community-integrated home-based depression treatment in older adults. *JAMA* 2004;**291**(13):1569-77.

**Ciechanowki 2010 *(published data only)***

Chaytor N, Ciechanowski P, Miller J, Fraser R, Russo J, Unutzer J, et al. Long-term outcomes from the PEARLS randomized trial for the treatment of depression in patients with epilepsy. *Epilepsy and Behavior* 2011;**20**(3):545–9.

*Ciechanowski P, Chaytor N, Miller J, Fraser R, Russo J, Unutzer J, et al. PEARLS depression treatment for individuals with epilepsy: a randomized controlled trial. *Epilepsy & Behavior* 2010;**19**(3):225-31.

**Cole 2006 (*published data only)***

*Cole MG, McCusker J, Elie M, Dendukuri N, Latimer E, Belzile E. Systematic detection and multidisciplinary care of depression in older medical inpatients: a randomized trial. *Canadian Medical Association Journal* 2006;**174**(1):38-44.

**Datto 2003 *(published data only)***

*Datto CJ, Thompson R, Horowitz D, Disbot M, Oslin DW. The pilot study of a telephone disease management program for depression. *General Hospital Psychiatry* 2003;**25**(3):169-77.

**Davidson 2013 (published data only)**

*Davidson KW, Bigger JT, Burg MM, Carney RM, Chaplin WF, Czajkowski S, et al. Centralized, stepped, patient preference-based treatment for patients with post-acute coronary syndrome depression: CODIACS vanguard randomized controlled trial. *JAMA Internal Medicine* 2013;**173**(11):997-1004.

**Dietrich 2004 *(published data only)***

Corson K, Gerrity MS, Dobscha SK. Screening for depression and suicidality in a VA primary care setting: 2 items are better than 1 item. *AmericanJournal ofManaged Care*2004;**10**(11Pt2):839–45.

Dietrich AJ. Improving primary care for PTSD: Lessons from the RESPECT-Depression Project. 158th Annual Meeting of the American Psychiatric Association; 2005 May 21-26; Atlanta,GA.2005:No. 23B.

*Dietrich AJ, Oxman TE, Williams JW, Schulberg HC, Bruce ML, Lee PW, et al. Re-engineering systems for the treatment of depression in primary care: cluster randomised controlled trial. BMJ 2004;**329**(7466):602.

Dietrich AJ, Oxman TE, Williams JW Jr, Kroenke K, Schulberg HC,Bruce M,et al. Going to scale: re-engineering systems for primary care treatment of depression. *Annals of Family Medicine* 2004;**2**(4):301–4.

Dobscha SK, Corson K, Gerrity M. Comorbid PTSD and alcohol abuse in veterans with elevated depression scores. 157th Annual Meeting of the American Psychiatric Association; 2004May 1-6; New York, NY. 2004:NR555.

Dobscha SK, Corson K, Solodky J, Gerrity MS. Use of videoconferencing for depression research: enrollment, retention, and patient satisfaction. *Telemedicine Journal and E-Health* 2005;**11**(1):84–9.

Kroenke K, Shen J, Oxman TE, Williams JW Jr, Dietrich AJ. Impact of pain on the outcomes of depression treatment: results from the RESPECT trial. *Pain* 2008;**134**(1-2): 209–15.

Lee PW, Dietrich AJ, Oxman TE, Williams JW Jr, Barry SL. Sustainable impact of a primary care depression intervention. *Journal of the American Board of Family Medicine*2007;**20**(5):427–33.

Lee PW, Schulberg HC, Raue PJ, Kroenke K. Concordance between thePHQ-9andtheHSCL-20 in depressed primary care patients. Journal of Affective Disorders 2007;**99**(1-3): 139–45.

Nutting PA, Gallagher K, Riley K, White S, Dickinson WP, Korsen N, et al. Care management for depression in primary care practice: findings from the RESPECT-Depression trial. *Annals of Family Medicine* 2008;**6**(1):30–7.

Nutting PA, Gallagher KM, Riley K, White S, Dietrich AJ, Dickinson WP. Implementing a depression improvement intervention in five health care organizations: Experience from the RESPECT-Depression trial. Administration and Policy in Mental Health 2007;**34**(2):127–37.

Oxman TE, Schulberg EC, Greenberg RL, Dietrich AJ, Williams JW Jr, Nutting PA, et al. A fidelity measure for integrated management of depression in primary care. *Medical Care* 2006;**44**(11):1030–7.

Schulberg HC, Lee PW, Bruce ML, Raue PJ, Lefever JJ, Williams JW Jr, et al. Suicidal ideation and risk levels among primary care patients with uncomplicated depression. *Annals of Family Medicine* 2005;**3**(6):523–8.

**Dwight-Johnson 2005 (*published data only)***

*Dwight-Johnson M, Ell K, Lee P-J. Can collaborative care address the needs of low-income Latinas with comorbid depression and cancer? Results from a randomized pilot study. *Psychosomatics* 2005;**46**(3):224-32.

**Dwight-Johnson 2010 (*published and unpublished data)***

*Dwight-Johnson M, Lagomasino I, Hay J, Zhang L, Tang L, Green J, et al. Effectiveness of collaborative care in addressing depression treatment preferences among low-income Latinos. *Psychiatric Services* 2010; **61**(11):1112-8.

**Dwight-Johnson 2011 (*published data only)***

*Dwight-Johnson M, Aisenberg E, Golinelli D, Hong S, O'Brien M, Ludman E. Telephone-based cognitive-behavioral therapy for Latino patients living in rural areas: a randomized pilot study. *Psychiatric Services* 2011;**62**(8):936-42.

**Ell 2007 (*published data only)***

*Ell K, Unützer J, Aranda M, Gibbs NE, Lee P-J, Xie B. Managing depression in home health care: a randomized clinical trial. *Home Health Care Services Quarterly* 2007;**26**(3):81-104.

**Ell 2008 (*published data only)***

Ell K,Quon B, Quinn DI, Dwight-Johnson M, Wells A, Lee PJ, et al. Improving treatment of depression among low income patients with cancer: the design of the ADAPt-C study. *General Hospital Psychiatry* 2007; **29**(3):223–31.

Ell K ,Xie B, Kapetanovic S, Quinn DI, Lee PJ, Wells A, et al. One-year follow-up of collaborative depression care for low-income, predominantly Hispanic patients with cancer. *Psychiatric Services* 2011;**62**(2):162–70.

*Ell K, Xie B, Quon B, Quinn DI, Dwight-Johnson M, Lee P-J. Randomized controlled trial of collaborative care management of depression among low-income patients with cancer. *Journal of Clinical Oncology* 2008;**26**(27):4488-96.

**Ell 2010 *(published data only)***

Ell K, Katon W, Cabassa LJ, Xie B, Lee PJ, Kapetanovic S, et al. Depression and diabetes among low-income Hispanics: design elements of a socioculturally adapted collaborative care model randomized controlled trial. *International Journal of Psychiatry in Medicine* 2009;**39**(2):113–32.

*Ell K, Katon W, Xie B, Lee P-J, Kapetanovic S, Guterman J, et al. Collaborative Care Management of Major Depression Among Low-Income, Predominantly Hispanic Subjects With Diabetes A randomized controlled trial. *Diabetes Care* 2010;**33**(4):706-13.

Ell K, Katon W, Xie B, Lee PJ, Kapetanovic S,Guterman J, et al. One-year post collaborative depression care trial outcomes among predominantly Hispanic diabetes safety net patients. *General Hospital Psychiatry* 2011;**33**(5):436–42.

Ell K, Lee PJ, Xie B. Depression care for low-income, minority, safety net clinic populations with comorbid illness. *Research on Social Work Practice* 2010;**20**(5):467–75.

Hay JW, Katon WJ, Ell K, Lee PJ, Guterman JJ. Cost effectiveness analysis of collaborative care management of major depression among low-income, predominantly Hispanics with diabetes. Journal of Mental Health Policy and Economics. Proceedings of the 10th Workshop on Costs and Assessment in Psychiatry; 2011 Mar 25-27; Venice, Italy. 2011; Vol. 14:S11.

Palinkas LA, Ell K, Hansen M, Cabassa L, Wells A. Sustainability of collaborative care interventions in primary care settings. *Journal of Social Work* 2011;**11**(1):99–117.

**Finley 2003 *(published data only)***

*Finley PR, Rens HR, Pont JT, Gess SL, Louie C, Bull SA, et al. Impact of a collaborative care model on depression in a primary care setting: a randomized controlled trial. *Pharmacotherapy* 2003;**23**(9):1175-85.

**Fortney 2007 *(published data only)***

Deen TL, Fortney JC, Pyne JM. Relationship between satisfaction, patient-centered care, adherence and outcomes among patients in a collaborative care trial for depression Administration and Policy in Mental Health and Mental Health Services Research 2011;**38**(5):345–55.

Edlund MJ, Fortney JC, Reaves CM, Pyne JM, Mittal D. Beliefs about depression and depression treatment among depressed veterans. *MedicalCare*2008;**46**(6):581–9.

Fortney JC, MacIejewski ML, Tripathi SP, Deen TL, Pyne JM. A budget impact analysis of telemedicine-based collaborative care for depression. *MedicalCare*2011;**49**(9): 872–80.

Fortney JC, Pyne JM, Edlund MJ ,Mittal D. Relationship between antidepressant medication possession and treatment response. *General Hospital Psychiatry* 2010;**32**(4): 377–9.

Fortney JC, Pyne JM, Edlund MJ, Robinson DE, Mittal D, Henderson KL. Design and implementation of the telemedicine-enhanced antidepressant management study. *General Hospital Psychiatry* 2006;**28**(1):18–26.

Fortney JC, Pyne JM, Edlund MJ, Stecker T, Mittal D, Robinson DE, et al. Reasons for antidepressant nonadherence among veterans treated in primary care clinics. *Journal of Clinical Psychiatry* 2011;**72**(6):827–34.

*Fortney JC, Pyne JM, Edlund MJ, Williams DK, Robinson DE, Mittal D, et al. A randomized trial of telemedicine-based collaborative care for depression. *Journal of General Internal Medicine* 2007;**22**(8):1086-93.

Fortney JC,Pyne JM, Steven CA, Williams JS, Hedrick RG, Lunsford AK, et al. A web-based clinical decision support system for depression care management. *American Journal of Managed Care* 2010;**16**(11):849–54.

Mittal D, Fortney JC, Pyne JM, Edlund MJ, Wetherell JL. Impact of comorbid anxiety disorders on health related quality of life among patients with major depressive disorder. *Psychiatric Services* 2006;**57**(12):1731–7.

Mittal D, Fortney JC, Pyne JM, Wetherell JL. Predictors of persistence of comorbid generalized anxiety disorder among veterans with major depressive disorder. *Journal of Clinical Psychiatry* 2011;**72**(11):1445–51.

Pyne JM, Fortney JC, Tripathi SP, Maciejewski ML, Edlund MJ, Williams DK. Cost-effectiveness analysis of a rural telemedicine collaborative care intervention for depression. *Archives of General Psychiatry* 2010;67(8):812–21.

**Fritsch 2007 *(published data only)***

*Fritsch R, Araya R, Solis J, Montt E, Pilowsky D, Rojas G. [A randomized trial of pharmacotherapy with telephone monitoring to improve treatment of depression in primary care in Santiago, Chile] [Spanish]. *Revista Médica de Chile* 2007;**135**(5):587-95.

**Gensichen 2009 *(published data only)***

Gensichen J, GuethlinC, Sarmand N, Sivakumaran D, Jager C, Mergenthal K, et al. Patients’ perspectives on depression case management in general practice –a qualitative study. *Patient Education and Counseling* 2012;**86**(1):114–9.

Gensichen J, Guthlin C, Kleppel V, Jager C, Mergenthal K, Gerlach FM, et al. Practice-based depression case management in primary care: A qualitative study on family doctors’ perspectives. *Family Practice* 2011;**28**(5):565–71.

Gensichen J, Jaeger C, Peitz M, Torge M, Guthlin C, Mergenthal K,et al. Health care assistants in primary care depression management: Role perception, burdening factors, and disease conception. *Annals of Family Medicine* 2009; **7**(6):513–9.

Gensichen J, Petersen JJ, Karroum T, Rauck S, Ludman E, Konig J, et al. Positive impact of a family practice-based depression case management on patient’s self-management. *General Hospital Psychiatry* 2011;**33**(1):23–8.

Gensichen J, Torge M, Peitz M, Wendt-Hermainski H, Beyer M, Rosemann T, et al. Case management for the treatment of patients with major depression in general practices -rationale, design and conduct of a cluster randomized controlled trial -PRoMPT (Primary care Monitoring for depressive Patient’s Trial) [ISRCTN66386086]-study protocol. *BMC Public Health* 2005;**5**:101.[DOI:10.1186/1471-2458-5-101]

*Gensichen J, vonKorff M, Peitz M, Muth C, Beyer M, Guthlin C, et al. Case management for depression by health care assistants in small primary care practices: a cluster randomized trial. *Annals of Internal Medicine* 2009;**151**(6): 369–78.

Kendrick T. Healthcare assistant case management may reduce depression symptoms in primary care patients with major depression more than usual care. *Evidence-Based Medicine* 2010;**15**(1):10–1.

**Gjerdingen 2009 *(published data only)***

*Gjerdingen D, Crow S, McGovern P, Miner M, Center B. Stepped care treatment of postpartum depression: impact on treatment, health, and work outcomes. *The Journal of the American Board of Family Medicine* 2009;**22**(5):473-82.

**Hedrick 2003 (*published data only)***

Goering P. Collaborative care speeds recovery from depression. *Evidence-Based Mental Health* 2003;**6**(4):116.

*Hedrick SC, Chaney EF, Felker B, Liu CF, Hasenberg N, Heagerty P, et al. Effectiveness of collaborative care depression treatment in Veterans' Affairs primary care. *Journal of General Internal Medicine* 2003;**18**(1):9-16.

Lin P, Campbell DG, Chaney EF, Liu CF, Heagerty P, Felker BL, et al. The influence of patient preference on depression treatment in primary care. *Annals of Behavioral Medicine* 2005;**30**(2):164–73.

Liu CF, Hedrick SC, Chaney EF,Heagerty P, Felker B, Hasenberg N, et al.Cost-effectiveness of collaborative care for depression in a primary care veteran population. *Psychiatric Services* 2003;**54**(5):698–704.

**Huffman 2011 *(published data only)***

Celano CM, Mastromauro CA, Lenihan EC, Januzzi JL, Rollman BL, Huffman JC. Association of baseline anxiety with depression persistence at 6 months inpatients with acute cardiac illness. *Psychosomatic Medicine* 2012;**74**(1): 93–9.

Huffman JC, Celano C, Mastromauro CA, Januzzi JL. Effects of baseline anxiety on depression persistence in a study of hospitalized cardiac patients. Psychosomatic Medicine. Proceedings from the 69^th^ Annual Meeting of the American Psychosomatic Society; 2011 Mar 9-12; San Antonio, TX United States. 2011;Vol. 73,issue3.

Huffman JC, Mastromauro CA, Sowden G, Fricchione GL, Healy BC, Januzzi JL. Impact of a depression care management program for hospitalized cardiac patients. *Circulation. Cardiovascular Quality and Outcomes* 2011;**4** (2):198–205.

*Huffman JC, Mastromauro CA, Sowden GL, Wittmann C, Rodman R, Januzzi JL. A collaborative care depression management program for cardiac inpatients: Depression characteristics and in-hospital outcomes. *Psychosomatics: Journal of Consultation Liaison Psychiatry* 2011;**52**(1):26–33.

**Huijbregts 2013 (published data only)**

*[Huijbregts KM](http://www.ncbi.nlm.nih.gov/pubmed?term=Huijbregts%20KM%5BAuthor%5D&cauthor=true&cauthor_uid=23068021), [de Jong FJ](http://www.ncbi.nlm.nih.gov/pubmed?term=de%20Jong%20FJ%5BAuthor%5D&cauthor=true&cauthor_uid=23068021), [van Marwijk HW](http://www.ncbi.nlm.nih.gov/pubmed?term=van%20Marwijk%20HW%5BAuthor%5D&cauthor=true&cauthor_uid=23068021), [Beekman AT](http://www.ncbi.nlm.nih.gov/pubmed?term=Beekman%20AT%5BAuthor%5D&cauthor=true&cauthor_uid=23068021), [Adèr HJ](http://www.ncbi.nlm.nih.gov/pubmed?term=Ad%C3%A8r%20HJ%5BAuthor%5D&cauthor=true&cauthor_uid=23068021),
[Hakkaart-van Roijen L](http://www.ncbi.nlm.nih.gov/pubmed?term=Hakkaart-van%20Roijen%20L%5BAuthor%5D&cauthor=true&cauthor_uid=23068021), et al. A target-driven collaborative care model for Major Depressive Disorder is effective in primary care in the Netherlands. A randomized clinical trial from the depression initiative. *Journal of Affective Disorders* 2013;**146**(3):328-37.

**Hunkeler 2000 *(published data only)***

Hunkeler EM, Hargreaves WA, Meresman J. Efficacy of nurse tele-health care as an augmentation to ssri treatment of depression in primary care. 153^rd^ Annual Meeting of the American Psychiatric Association; 2000 May 13-18; Chicago (IL). 2000:NR448.

Hunkeler EM, Hargreaves WA, Meresman J. Efficacy of nurse tele-health care as an augmentation to ssri treatment ofdepression in primary care. 155^th^ Annual Meeting of the American Psychiatric Association; 2002 May18-23rd; Philadelphia(PA).2000:NR448.

*Hunkeler EM, Meresman JF, Hargreaves WA, Fireman B, Berman WH, Kirsch AJ, etal. Efficacy of nurse telehealth care and peer support in augmenting treatment of depression in primary care. *Archives of Family Medicine* 2000;**9**(8): 700–8.

Meresman J, Hunkeler E, Hargreaves W. The nurse telecare project for treating depression: A progress report. 35th International Meeting of the Society for Psychotherapy Research; 2004 June16-19;Rome. 2004:170

.

**Katon 1995a *(published data only)***

*Katon W, Von Korff M, Lin E, Walker E, Simon GE, Bush T, et al. Collaborative management to achieve treatment guidelinesimpact on depression in primary care. *JAMA: the journal of the American Medical Association* 1995;**273**(13):1026-31.

**Katon 1995b *(published data only)***

*Katon W, Von Korff M, Lin E, Walker E, Simon GE, Bush T, et al. Collaborative management to achieve treatment guidelinesimpact on depression in primary care. *JAMA: the journal of the American Medical Association* 1995;**273**(13):1026-31.

**Katon 1996a *(published data only)***

*Katon W, Robinson P, Von Korff M, Lin E, Bush T, Ludman E, et al. A multifaceted intervention to improve treatment of depression in primary care. *Archives of General Psychiatry* 1996;**53**(10):924.

Robinson P, Katon W, VonKorff M, Bush T, Simon G, Lin E, et al. The education of depressed primary care patients: what do patients think of interactive booklets and a video? *Journal of Family Practice* 1997;**44**(6):562–71.

**Katon 1996b *(published data only)***

*Katon W, Robinson P, Von Korff M, Lin E, Bush T, Ludman E, et al. A multifaceted intervention to improve treatment of depression in primary care. *Archives of General Psychiatry* 1996;**53**(10):924.

Robinson P, Katon W, VonKorff M, Bush T, Simon G, Lin E, et al. The education of depressed primary care patients: what do patients think of interactive booklets and a video? *Journal of Family Practice* 1997;**44**(6):562–71.

**Katon 1999 *(published data only)***

*Katon W, Von Korff M, Lin E, Simon G, Walker E, Unutzer J, et al. Stepped collaborative care for primary care patients with persistent symptoms of depression: a randomized trial. *Archives of General Psychiatry* 1999;**56**(12):1109-15.

Katon WJ, Russo JE, Von Korff M, Lin EH, Ludman E, Ciechanowski PS. Long-term effects on medical costs of improving depression outcomes in patients with depression and diabetes. *Diabetes Care* 2008;**31**(6):1155–9.

Shaughnessy A. In patients who do not respond to antidepressants or who are at risk for recurrence, is collaborative care effective? *Evidence-Based Practice* 2003;**6** (2):9.

Simon GE, Katon WJ, Von Korff M, Unuetzer J, Lin EH, WalkerEA,et al. Cost-effectiveness of a collaborative care program for primary care patients with persistent depression. *American Journal of Psychiatry* 2001;**158**(10): 1638–44.

Unutzer J, Katon WJ, Russo J, Simon G, Von Korff M, Lin E, et al. Willingness to pay for depression treatment in Primary care. *Psychiatric Services* 2003;**54**(3):340–5.

Walker EA, Katon WJ, Russo J, Von Korff M, Lin E, Simon G, et al. Predictors of outcome in a primary care depression trial. *Journal of General Internal Medicine* 2000;**15**(12): 859–67.

**Katon 2001 *(published data only)***

Bullock R. A depression relapse prevention programme improved adherence to medication and depressive symptoms but did not decrease relapses. *Evidence-Based Mental Health* 2001;**4**(4):113.

Gopinath S, Katon WJ, Russo JE, Ludman EJ. Clinical factors associated with relapse in primary care patients with chronic or recurrent depression. *Journal of Affective Disorders* 2007;**101**(1-3):57–63.

*Katon W, Rutter C, Ludman E J, Von Korff M, Lin E, Simon G, et al. A randomized trial of relapse prevention of depression in primary care. *Archives of General Psychiatry* 2001;**58**(3):241–7.

Lin EH, Von Korff M, Ludman EJ, Rutter C, Bush TM, Simon GE, et al. Enhancing adherence to prevent depression relapse in primary care. General Hospital Psychiatry2003;**25** (5):303–10.

Ludman E, Katon W, Bush T, Rutter C, Lin E, Simon G, et al. Behavioural factors associated with symptom outcomes in a primary care-based depression prevention intervention trial. *PsychologicalMedicine*2003; **33**(6):1061–70.

Ludman E, Von Korff M, Katon W, Lin E, Simon G, WalkerE,et al. The design, implementation, and acceptance of a primary care-based intervention to prevent depression relapse. International *Journal of Psychiatry in Medicine* 2000; **30**(3):229–45.

Simon GE, Von Korff M, Ludman EJ, Katon WJ, Rutter C, Unutzer J,et al. Cost-effectiveness of a program to prevent depression relapse in primary care. *Medical Care* 2002;**40**(10):941–50.

Von Korff M, Katon W, Rutter C, Ludman E, Simon G, Lin E, et al. Effect on disability outcomes of a depression relapse prevention program. *PsychosomaticMedicine* 2003; **65**(6):938–43.

**Katon 2004 *(published data only)***

Ciechanowski PS, Russo JE, Katon WJ. The difficult patient: A developmental perspective using an attachment theoretical framework. 63rd Annual Meeting of the American Psychosomatic Society; 2005 March 2-5; Vancouver,Canada. 2005:A122.

Ciechanowski PS, Russo JE, Katon WJ, Von Korff M, Simon GE, Lin EHB, et al. The association of patient relationship style and outcomes in collaborative care treatment for depression in patients with diabetes. *Medical Care* 2006;**44**(3):283–91.

Davidson MB, Echeverry D. Collaborative care for depression and chronic illnesses [comment]. *New England Journal of Medicine* 2011; 364(13):1278; author reply1278-9.

Gask L, Ludman E, Schaefer J. Qualitative study of an intervention for depression among patients with diabetes: how can we optimize patient-professional interaction? *Chronic Illness* 2006; **2**(3):231–42.

Glasgow RE, Price DW. Individualised treatment improves depression in people with depression and diabetes. *Evidence-Based Mental Health* 2005;**8**(2):40.

Katon W, Russo J, Von Korff M, Lin E, Simon G, Bush T, et al. Long-term effects of a collaborative care intervention in persistently depressed primary care patients. *Journal of General Internal Medicine* 2002;**17**(10):741–8.

Katon W, Von Korff M, Lin E, Simon G, Ludman E, Bush T, et al. Improving primary care treatment of depression among patients with diabetes mellitus: the design of the pathways study. *General Hospital Psychiatry* 2003; **25**(3): 158–68.

Katon WJ, Von Korff M, Lin E, Simon G, Ciechanowski P, Ludman E, et al. The PATHWAYS study: A randomized trial of collaborative care in patients with diabetes and depression. 157th Annual Meeting of the American Psychiatric Association; 2004 May 1-6; New York(NY). 2004:No. 9A.

*Katon WJ, Von Korff M, Lin EH, Simon G, Ludman E, Russo J, et al. The Pathways Study: a randomized trial of collaborative care in patients with diabetes and depression. *Archives of General Psychiatry* 2004;**61**(10):1042–9.

Kinder LS, Katon WJ, Ludman E, Russo J, Simon G, Lin EH, et al. Improving depression care in patients with diabetes and multiple complications. *Journal of General Internal Medicine* 2006;21(10):1036–41.

Lin EH, Katon W, Rutter C, Simon GE, Ludman EJ, Von Korff M, et al. Effects of enhanced depression treatment on diabetes self-care. *Annals of Family Medicine* 2006;**4**(1): 46–53.

Lin EH, Katon W, Rutter C, Von Korff M, Ludman EJ, Simon GE, et al. Effects of enhanced depression care on diabetes self management: A randomized-controlled trial. 63rd Annual Meeting of the American Psychosomatic Society; 2005 March2-5; Vancouver, Canada. 2005:A23.

O’Malley PG. Collaborative care for depression in patients with diabetes increased depression-free days and had economic benefit. *ACP Journal Club* 2007;**146**(3):78.

Simon GE, Katon WJ, Lin EH, Rutter C, Manning WG, Von Korff M, et al. Cost-effectiveness of systematic depression treatment among people with diabetes mellitus. *Archives of General Psychiatry* 2007;**64**(1):65–72.

Upchurch SL. A collaborative care intervention improved depression outcomes, but not glycaemic control, in diabetes and comorbid depression. *Evidence-Based Nursing* 2005;**8**(3):81.

**Katon 2010 *(published data only)***

Hung W. Collaborative care for patients with depression improves chronic disease management: Commentary. *Journal of Clinical Outcomes Management* 2011;**18**(4): 156–7.

Katon W, Lin EH, Von Korff M, Ciechanowski P, Ludman E, Young B, et al. Integrating depression and chronic disease care among patients with diabetes and/or coronary heart disease: the design of the TEAMcare study. *Contemporary Clinical Trials* 2010;**31**(4):312–22.

Katon W, Russo J, Lin EHB , Schmittdiel J, Ciechanowski P, Ludman E, et al. Cost-effectiveness of a Multicondition Collaborative Care Intervention: *A Randomized Controlled Trial. Archives of General Psychiatry* 2012;**69**(5):506–514.

*Katon WJ, Lin EH, Von Korff M, Ciechanowski P, LudmanEJ,YoungB,et al. Collaborative care for patients with depression and chronic illnesses. *New England Journal of Medicine* 2010;**363**(27):2611–20.

Katon WJ, Lin EHB, Von Korff M. The authors reply: ”Collaborative care for depression and chronic illnesses“ [Reply to comment]. *New England Journal of Medicine* 2011;**364**(13):1278–9.

McGregor M, Lin EH, Katon WJ. TEAMcare: an integrated multicondition collaborative care program for chronic illnesses and depression. *Journal of Ambulatory Care Management* 2011;**34**(2):152–62.

Von Korff M, Katon WJ, Lin EHB, Ciechanowski P, Peterson D, Ludman EJ, et al. Functional outcomes of multi-condition collaborative care and successful ageing: Results of randomised trial. *BMJ* 2011; **343**(7833):1083.

**Katzelnick 2000 *(published data only)***

Katzelnick DJ, Simon GE, Pearson SD, Manning WG, Helstad CP, Henk HJ. Randomized trial of a depression management program in high utilizers of medical care. 151st Annual Meeting of the American Psychiatric Association; 1998 May 30-Jun 4; Toronto, Ontario, Canada. 1998.

*Katzelnick DJ, Simon GE, Pearson SD, Manning WG, Helstad CP, Henk HJ, etal. Randomized trial of a depression management program in high utilizers of medical care. *Archives of Family Medicine* 2000;**9**(4):345–51.

Katzelnick DJ, Simon GE, Pearson SD, Manning WG, Helstad CP, Henks HJ. Clinical outcomes care study. 152nd Annual Meeting of the American Psychiatric Association; 1999 May15-20; Washington (DC). 1999.

Peveler R. A depression management programme reduced depression in frequent users of healthcare but did not reduce healthcare visits. *Evidence-Based Mental Health* 2001;**4**(3): 78–9.

Simon GE, Manning WG, Katzelnick DJ, Pearson SD, Henk HJ, Helstad CP. Cost-effectiveness of systematic depression treatment for high utilizers of general medical care. *Archives of General Psychiatry* 2001;**58**(2):181–7.

**Kroenke 2010 *(published data only)***

Brown LF, Kroenke K, Theobald DE, Wu J, Tu W. The association of depression and anxiety with health-related quality of life in cancer patients with depression and/or pain. *Psycho-Oncology* 2010; **19**(7):734–41.

Johns SA, Kroenke K, Theobald DE, Wu J,Tu W.Telecare management of pain and depression in patients with cancer: patient satisfaction andpredictors of use. *Journal of Ambulatory Care Management* 2011;**34**(2):126–39.

Kroenke K, Theobald D,Norton K,Sanders R,Schlundt S, McCalley S,et al. The Indiana Cancer Pain and Depression (INCPAD) trial Design of a telecare management intervention for cancer-related symptoms and baseline characteristics of study participants. *General Hospital Psychiatry* 2009;**31**(3):240–53.

Kroenke K, Theobald D, Wu J, Loza JK, Carpenter JS, TuW. The association of depression and pain with health related quality of life, disability, and health care use in cancer patients. *Journal of Pain and Symptom Management* 2010;**40**(3):327–41.

*Kroenke K, Theobald D, Wu J, Norton K, Morrison G, Carpenter J ,et al. Effect of telecare management on pain and depression inpatients with cancer. *JAMA* 2010;**304**(2): 163–71.

**Landis 2007 *(published data only)***

*Landis SE, Gaynes BN, Morrissey JP, Vinson N, Ellis AR, Domino ME. Generalist care managers for the treatment of depressed medicaid patients in North Carolina: A pilot study. *BMC family practice* 2007;**8**(1):7.

**Lobello 2010 *(published data only)***

*Lobello K, Reddy S, Musgnung J, Pedersen R, Ninan PT. Patient outcomes with education, drug therapy, and support: a study of venlafaxine ER-treated outpatients with major depressive disorder. *Psychopharmacology Bulletin* 2010;**43**(2):28.

**Ludman 2007a *(published data only)***

*Ludman E, Simon G, Grothaus L, Luce C, Markley D, Schaefer J. A pilot study of telephone care management and structured disease self-management groups for chronic depression. *Psychiatric Services* 2007;**58**(8):1065-72.

**Ludman 2007b *(published data only)***

*Ludman E, Simon G, Grothaus L, Luce C, Markley D, Schaefer J. A pilot study of telephone care management and structured disease self-management groups for chronic depression. *Psychiatric Services* 2007;**58**(8):1065-72.

**Ludman 2007c *(published data only)***

*Ludman E, Simon G, Grothaus L, Luce C, Markley D, Schaefer J. A pilot study of telephone care management and structured disease self-management groups for chronic depression. *Psychiatric Services* 2007;**58**(8):1065-72.

**Mann 1998 *(published data only)***

*Mann A, Blizard R, Murray J, Smith J, Botega N, MacDonald E, et al. An evaluation of practice nurses working with general practitioners to treat people with depression. *The British Journal of General Practice* 1998;**48**(426):875.

**McCusker 2008 (*published data only)***

*McCusker J, Cole M, Yaffe M, Cappeliez P, Dawes M, Sewitch M, et al. Project DIRECT: Pilot study of a collaborative intervention for depressed seniors. *Canadian Journal of Community Mental Health* 2008;**27**(2):201-18.

**McMahon 2007 *(published data only)***

*McMahon L, Foran KM, Forrest SD, Taylor ML, Ingram G, Rajwal M, et al. Graduate mental health worker case management of depression in UK primary care: a pilot study. *The British Journal of General Practice* 2007;**57**(544):880-5.

**Menchetti 2013 (published data only)**

*Menchetti M1, Sighinolfi C, Di Michele V, Peloso P, Nespeca C, Bandieri PV, et al. Effectiveness of collaborative care for depression in Italy. A randomized controlled trial*. General Hospital Psychiatry* 2013;**35(**6):579-86.

**Morgan 2012 (published data only)**

*[Morgan MA](http://www.ncbi.nlm.nih.gov/pubmed?term=Morgan%20MA%5BAuthor%5D&cauthor=true&cauthor_uid=23355671), [Coates MJ](http://www.ncbi.nlm.nih.gov/pubmed?term=Coates%20MJ%5BAuthor%5D&cauthor=true&cauthor_uid=23355671), [Dunbar JA](http://www.ncbi.nlm.nih.gov/pubmed?term=Dunbar%20JA%5BAuthor%5D&cauthor=true&cauthor_uid=23355671), [Reddy P](http://www.ncbi.nlm.nih.gov/pubmed?term=Reddy%20P%5BAuthor%5D&cauthor=true&cauthor_uid=23355671), [Schlicht K](http://www.ncbi.nlm.nih.gov/pubmed?term=Schlicht%20K%5BAuthor%5D&cauthor=true&cauthor_uid=23355671), [Fuller J](http://www.ncbi.nlm.nih.gov/pubmed?term=Fuller%20J%5BAuthor%5D&cauthor=true&cauthor_uid=23355671). The TrueBlue model of collaborative care using practice nurses as case managers for depression alongside diabetes or heart disease: a randomised trial. *BMJ Open* 2013;**3**(1).

**Oslin 2003 *(published data only)***

Oslin D. Telephone disease management for depression and at-risk drinking. International Psychogeriatrics. Proceedings of the International Psychogeriatric Association Eleventh International Congress;2003 Aug17-22,Chicago. 2003;Vol. 15.

*Oslin DW, Sayers S, Ross J, Kane V, Ten Have T, Conigliaro J, et al. Disease management for depression and at-risk drinking via telephone in an older population of veterans. *Psychosomatic Medicine* 2003;**65**(6):931-7.

**Patel 2010 *(published data only)***

Patel V, Weiss H, Mann A. Predictors of outcome in patients with common mental disorders receiving a brief psychological treatment: An exploratory analysis of a randomized controlled trial from Goa, India. *African Journal of Psychiatry (South Africa)* 2010;**13**(4):291–6.

*Patel V, Weiss HA, Chowdhary N, Naik S,Pednekar S, Chatterjee S, et al. Effectiveness of an intervention led by lay health counsellors for depressive and anxiety disorders in primary care in Goa, India(MANAS):A cluster randomised controlled trial. *Lancet* 2010;**376**(9758):2086–95.

Patel V, Weiss HA, Chowdhary N, Naik S, Pednekar S, Chatterjee S, et al. Lay health worker led intervention for depressive and anxiety disorders in India: impact on clinical and disability outcomes over 12 months. *British Journal of Psychiatry* 2011;**199**(6):459–66.

Patel VH, Kirkwood BR, Pednekar S,Araya R, King M, Chisholm D,et al. Improving the outcomes of primary care attenders with common mental disorders in developing countries: A cluster randomized controlled trial of a collaborative stepped care intervention in Goa, India. *Trials* 2008;**9**:4.

**Piette 2011 *(published data only)***

Piette J, Duffy S, Torres T, Vogel M, Himle J, Richardson C, et al. 12-month outcomes from a randomized trial of telephone cognitive behavioral therapy for depressed patients with type 2 diabetes. *Journal of General Internal Medicine. Proceedings of the 33rd Annual Meeting of the Society of General Internal Medicine; 2010 Apr28-May 1, Minneapolis(MN)* 2010;**25**(Suppl3):S205.

*Piette JD, Richardson C, Himle J, Duffy S, Torres T, Vogel M, et al. A randomized trial of telephonic counseling plus walking for depressed diabetes patients. *Medical Care* 2011;**49**(7):641–8.

**Pynne 2011 *(published data only)***

Curran GM, Pyne J, Fortney JC, Gifford A, Asch SM, Rimland D, et al. Development and implementation of collaborative care for depression in HIV clinics. *AIDS Care –Psychological and Socio-Medical Aspects of AIDS/HIV* 2011; **23**(12):1626–36.

*Pyne JM, Fortney JC, Curran GM, Tripathi S, Atkinson JH, Kilbourne AM, et al. Effectiveness of collaborative care for depression in human immunodeficiency virus clinics. *Archives of Internal Medicine* 2011; (1):23–31.

**Richards 2008a *(published data only)***

McMillan D, Gilbody S, Richards D. Defining successful treatment outcome in depression using the PHQ-9: A comparison of methods. *Journal of Affective Disorders* 2010; **127**(1-3):122–9.

Richards D. Models of Stepped Care: evidence from the first UK trials of collaborative care for depression and stepped care for common mental health problems. 34thAnnual Conference of the British Association for Behavioural and Cognitive Psychotherapies; 2006 July 19-21, Warwick. 2006:60.

*Richards DA, Lovell K, Gilbody S, Gask L, Torgerson D, Barkham M, et al. Collaborative care for depression in UK primary care: A randomized controlled trial: Corrigendum. *Psychological Medicine* 2009;**39**(4):701.

Richards DA, Lovell K, Gilbody S, Gask L, Torgerson, D, Barkham M, et al. Collaborative care for depression in UK primary care: a randomized controlled trial [Erratum published in Psychological Medicine 2009;39(4):701]. *PsychologicalMedicine*2008;**38**(2):279–87.

Simpson A, Richards D, Gask L, Hennessy S, Escott D. Patients’ experiences of receiving collaborative care for the treatment of depression in the UK: a qualitative investigation. *Mental Health in Family Medicine* 2008;**5**(2): 95–104.

**Richards 2008b *(published data only)***

McMillan D, Gilbody S, Richards D. Defining successful treatment outcome in depression using the PHQ-9: A comparison of methods. *Journal of Affective Disorders* 2010; **127**(1-3):122–9.

Richards D. Models of Stepped Care: evidence from the first UK trials of collaborative care for depression and stepped care for common mental health problems. 34thAnnual Conference of the British Association for Behavioural and Cognitive Psychotherapies; 2006 July 19-21, Warwick. 2006:60.

*Richards DA, Lovell K, Gilbody S, Gask L, Torgerson D, Barkham M, et al. Collaborative care for depression in UK primary care: A randomized controlled trial: Corrigendum. *Psychological Medicine* 2009;**39**(4):701.

Richards DA, Lovell K, Gilbody S, Gask L, Torgerson, D, Barkham M, et al. Collaborative care for depression in UK primary care: a randomized controlled trial [Erratum published in Psychological Medicine 2009;39(4):701]. *PsychologicalMedicine*2008;**38**(2):279–87.

Simpson A, Richards D, Gask L, Hennessy S, Escott D. Patients’ experiences of receiving collaborative care for the treatment of depression in the UK: a qualitative investigation. *Mental Health in Family Medicine* 2008;**5**(2): 95–104.

**Richards 2013 *(published data)***

Richards DA. Multi-centre randomised controlled trial of collaborative care for depression [CADET (CollAborative Depression Trial) [Is collaborative care more clinically and cost effective than usual care in the management of patients with moderate to severe depression in UK primary care?] [ISRCTN32829227]. Current Controlled Trials [www.controlled-trials.com] 2009.

Richards DA Hughes-Morley A, Hayes RA, Araya R, Barkham M ,Bland JM, et al. Collaborative depression trial (CADET): Multi-centre randomised controlled trial of collaborative care for depression –Study protocol. *BMC Health Services Research* 2009;**16**(9):188.

*Richards DA, Hill JJ, Gask L, Lovell K, Chew-Graham C, Bower P, et al. Clinical effectiveness of collaborative care for depression in UK primary care (CADET): cluster randomised controlled trial. *BMJ* 2013;**347**: f4913

**Rojas 2007 *(published data only)***

*Rojas G, Fritsch R, Solis J, Jadresic E, Castillo C, Gonzalez M, et al. Treatment of postnatal depression in low income mothers in primary-care clinics in Santiago, Chile: a randomised controlled trial. *Lancet* 2007;**370**(9599): 1629–37.

Zayas LH. Six-month multicomponent intervention improves postnatal depression in low-income settings. *Evidence-Based Mental Health* 2008; **11**(3):80.

**Rollman 2009 *(published data only)***

Gallagher R. Telephone-delivered collaborative care for post-CABG depression is more effective than usual care for improving quality of life related to mental health. *Evidence-Based Nursing* 2010;**13**(2):37.

Morone NE, Weiner DK, Belnap BH, Karp JF, Mazumdar S, Houck PR, et al. The impact of pain and depression on recovery after coronary artery bypass grafting. *Psychosomatic Medicine* 2010;**72**(7):620–5.

Rollman BL, Belnap BH, LeMenager MS, Mazumdar S, Houck PR, Counihan PJ, et al. Telephone-delivered collaborative care for treating post-CABG depression: a randomized controlled trial. *JAMA* 2009;**302**(19): 2095–103.

Rollman BL, Belnap BH, LeMenager MS, Mazumdar S, Schulberg HC,Reynolds CF 3rd. The Bypassing the Blues treatment protocol: stepped collaborative care for treating post-CABG depression. *Psychosomatic Medicine* 2009;**71**(2):217–30.

*Rollman BL, Herbeck Belnap B, LeMenager MS, Mazumdar S,Houck PR,Counihan PJ, et al. Telephone delivered collaborative care for treating post-CABG depression: a randomized controlled trial. *JAMA* 2009;**302**(19):2095–103.

Schulberg HC, Belnap BH, Houck PR, Mazumdar S, Reynolds CF III, Rollman BL. Treating post-CABG depression with telephone-delivered collaborative care: Does patient age affect treatment and outcome? *The American Journal of Geriatric Psychiatry* 2011;**19**(10): 871–80.

Sriwattanakomen R, Mazumdar S, Belnap B, Houck P, Reynolds C, Rollman B. The effect of comorbid anxiety on post-CABG depressed patients’ mental health related quality of life. *Journal of General Internal Medicine.* Proceedings of the 33rd Annual Meeting of the Society of General Internal Medicine; 2010 Apr 28-May 1; Minneapolis (MN).2010:S401.

Tindle HA, Belnap BH, Hum B, Houck PR, Mazumdar S, Scheier M, et al. Optimism, depression, and depression remission after CABG surgery. Psychosomatic Medicine. Proceedings of the 69^th^ Annual Meeting of the American Psychosomatic Society; 2011 Mar 9-12; SanAntonio(TX). 2011; Vol. 73:A49.

Tully PJ. Randomised controlled trial: telephone-delivered collaborative care for post-CABG depression is more effective than usual care for improving mental-health-related quality of life. *Evidence-Based Medicine* 2010;**15**(2):57–8.

**Ross 2008 *(published data only)***

*Ross JT, Eakin AC, Suzanne Difilippo RN C, Oslin DW. A randomized controlled trial of a close monitoring program for minor depression and distress. *Journal of General Internal Medicine* 2008;**23**(9):1379-85.

**Rost 2001a *(published data only)***

Adams SJ ,Xu S, Dong F, Fortney J, Rost K. Differential effectiveness of depression disease management for rural and urban primary care patients. *Journal of Rural Health* 2006;**22**(4):343–50.

Clever SL, Ford DE, Rubenstein LV, Rost KM, Meredith LS, Sherbourne CD, et al. Primary care patients’ involvement in decision-making is associated with improvement in depression. *Medical Care* 2006;**44**(5):398–405.

Dickinson LM, Rost K, Nutting PA, Elliott CE, Keeley RD, Pincus H. RCT of a care manager intervention for major depression in primary care:2-Year costs for patients with physical vs psychological complaints. *Annals of Family Medicine* 2005;**3**(1):15–22.

Keeley RD, Smith JL, Nutting PA, Miriam Dickinson L, Perry Dickinson W, Rost KM. Does a depression intervention result in improved outcomes for patients presenting with physical symptoms? *Journal of General Internal* *Medicine* 2004; **19**(6):615–23.

Lo Sasso AT, Rost K, Beck A. Modeling the impact of enhanced depression treatment on workplace functioning and costs: a cost-benefit approach. *Medical Care* 2006;**44**(4):352–8.

Nutting PA, Dickinson LM, Rubenstein LV, Keeley RD, Smith JL, Elliott CE. Improving detection of suicidal ideation among depressed patients in primary care. *Annals of Family Medicine* 2005;**3**(6):529–36.

Nutting PA, Rost K, Dickinson M, Werner JJ, Dickinson P, Smith JL, et al. Barriers to initiating depression treatment in primary care practice. *Journal of General Internal Medicine* 2002;**17**(2):103–11.

Pyne JM, Rost KM, Farahati F, Tripathi SP, Smith J,Williams DK, et al. One size fits some: the impact of patient treatment attitudes on the cost-effectiveness of a depression primary-care intervention. *Psychological Medicine* 2005;**35**(6):839–54.

Pyne JM, Rost KM, Zhang M, Williams DK, Smith J, Fortney J. Cost-effectiveness of a primary care depression intervention. *Journal of General Internal Medicine* 2003;**18**(6):432–41.

Pyne JM, Smith J, Fortney J, Zhang M, Williams DK, Rost K. Cost-effectiveness of a primary care intervention for depressed females. *Journal of Affective Disorders* 2003;**74**(1): 23–32.

Rost K, Adams S, Xu S, Dong F. Rural-urban differences in hospitalization rates of primary care patients with depression. *Psychiatric Services* 2007;**58**(4):503–8.

Rost K, Nutting P, Smith J, Werner J, Duan N. Improving depression outcomes in community primary care practice: A randomized trial of the QuEST intervention. *Journal of General Internal Medicine* 2001;**16**(3):143–9.

* Rost K, Nutting P, Smith JL, Elliott CE, Dickinson M. Managing depression as a chronic disease: a randomised trial of ongoing treatment in primary care. *BMJ* 2002;**325**(7370):934.

Rost K, Nutting PA, Smith J, Werner JJ. Designing and implementing a primary care intervention trial to improve the quality and outcome of care for major depression. *General Hospital Psychiatry* 2000;**22**(2):66–77.

Rost K, Pyne JM, Dickinson LM, LoSasso AT. Cost-effectiveness of enhancing primary care depression management on an ongoing basis. *Annals of Family Medicine* 2005;**3**(1):7–14.

Rost K, Smith JL, Dickinson M. The effect of improving primary care depression management on employee absenteeism and productivity. A randomized trial. *Medical Care* 2004;**42**(12):1202–10.

Rost KM, Duan N, Rubenstein LV, Ford DE, Sherbourne CD, Meredith LS, et al. The Quality Improvement for Depression collaboration: general analytic strategies for a coordinated study of quality improvement in depression care. *General Hospital Psychiatry* 2001;**23**(5):239–53.

Rost KM, Nutting P, Smith J, Werner J. Primary care training improves depression outcomes. 153rd Annual Meeting of the American Psychiatric Association; 2000 May 13-18, Chicago(IL).2000:No.36E.

Smith JL, Rost KM, Nutting PA, Elliott CE. Resolving disparities in antidepressant treatment and quality-of-life outcomes between uninsured and insured primary care patients with depression. *Medical Care* 2001;**39**(9):910–22.

Smith JL, Rost KM, Nutting PA, Elliott CE, Dickinson LM. Impact of ongoing primary care intervention on long term outcomes in uninsured and insured patients with depression. *Medical Care* 2002;**40**(12):1210–22.

Smith JL, Rost KM, Nutting PA, Elliott CE, Duan N. A primary care intervention for depression. *Journal of Rural Health* 2000;**16**(4):313–23.

Xu S ,Rost K, Dong F, Dickinson LM. Stakeholder benefit from depression disease management: Differences by rurality? *The Journal of Behavioral Health Services and Research* 2011;**38**(1):114–21.

**Rost 2001b *(published data only)***

Adams SJ ,Xu S, Dong F, Fortney J, Rost K. Differential effectiveness of depression disease management for rural and urban primary care patients. *Journal of Rural Health* 2006;**22**(4):343–50.

Clever SL, Ford DE, Rubenstein LV, Rost KM, Meredith LS, Sherbourne CD, et al. Primary care patients’ involvement in decision-making is associated with improvement in depression. *Medical Care* 2006;**44**(5):398–405.

Dickinson LM, Rost K, Nutting PA, Elliott CE, Keeley RD, Pincus H. RCT of a care manager intervention for major depression in primary care:2-Year costs for patients with physical vs psychological complaints. *Annals of Family Medicine* 2005;**3**(1):15–22.

Keeley RD, Smith JL, Nutting PA, Miriam Dickinson L, Perry Dickinson W, Rost KM. Does a depression intervention result in improved outcomes for patients presenting with physical symptoms? *Journal of General Internal* *Medicine* 2004; **19**(6):615–23.

Lo Sasso AT, Rost K, Beck A. Modeling the impact of enhanced depression treatment on workplace functioning and costs: a cost-benefit approach. *Medical Care* 2006;**44**(4):352–8.

Nutting PA, Dickinson LM, Rubenstein LV, Keeley RD, Smith JL, Elliott CE. Improving detection of suicidal ideation among depressed patients in primary care. *Annals of Family Medicine* 2005;**3**(6):529–36.

Nutting PA, Rost K, Dickinson M, Werner JJ, Dickinson P, Smith JL, et al. Barriers to initiating depression treatment in primary care practice. *Journal of General Internal Medicine* 2002;**17**(2):103–11.

Pyne JM, Rost KM, Farahati F, Tripathi SP, Smith J,Williams DK, et al. One size fits some: the impact of patient treatment attitudes on the cost-effectiveness of a depression primary-care intervention. *Psychological Medicine* 2005;**35**(6):839–54.

Pyne JM, Rost KM, Zhang M, Williams DK, Smith J, Fortney J. Cost-effectiveness of a primary care depression intervention. *Journal of General Internal Medicine* 2003;**18**(6):432–41.

Pyne JM, Smith J, Fortney J, Zhang M, Williams DK, Rost K. Cost-effectiveness of a primary care intervention for depressed females. *Journal of Affective Disorders* 2003;**74**(1): 23–32.

Rost K, Adams S, Xu S, Dong F. Rural-urban differences in hospitalization rates of primary care patients with depression. *Psychiatric Services* 2007;**58**(4):503–8.

Rost K, Nutting P, Smith J, Werner J, Duan N. Improving depression outcomes in community primary care practice: A randomized trial of the QuEST intervention. *Journal of General Internal Medicine* 2001;**16**(3):143–9.

*RostK, Nutting P, Smith JL, Elliott CE, Dickinson M. Managing depression as a chronic disease: a randomised trial of ongoing treatment in primary care. *BMJ* 2002;**325**(7370):934.

Rost K, Nutting PA, Smith J, Werner JJ. Designing and implementing a primary care intervention trial to improve the quality and outcome of care for major depression. *General Hospital Psychiatry* 2000;**22**(2):66–77.

Rost K, Pyne JM, Dickinson LM, LoSasso AT. Cost-effectiveness of enhancing primary care depression management on an ongoing basis. *Annals of Family Medicine* 2005;**3**(1):7–14.

Rost K, Smith JL, Dickinson M. The effect of improving primary care depression management on employee absenteeism and productivity. A randomized trial. *Medical Care* 2004;**42**(12):1202–10.

Rost KM, Duan N, Rubenstein LV, Ford DE, Sherbourne CD, Meredith LS, et al. The Quality Improvement for Depression collaboration: general analytic strategies for a coordinated study of quality improvement in depression care. *General Hospital Psychiatry* 2001;**23**(5):239–53.

Rost KM, Nutting P, Smith J, Werner J. Primary care training improves depression outcomes. 153rd Annual Meeting of the American Psychiatric Association; 2000 May 13-18, Chicago(IL).2000:No.36E.

Smith JL, Rost KM, Nutting PA, Elliott CE. Resolving disparities in antidepressant treatment and quality-of-life outcomes between uninsured and insured primary care patients with depression. *Medical Care* 2001;**39**(9):910–22.

Smith JL, Rost KM, Nutting PA, Elliott CE, Dickinson LM. Impact of ongoing primary care intervention on long term outcomes in uninsured and insured patients with depression. *Medical Care* 2002;**40**(12):1210–22.

Smith JL, Rost KM, Nutting PA, Elliott CE, Duan N. A primary care intervention for depression. *Journal of Rural Health* 2000;**16**(4):313–23.

Xu S ,Rost K, Dong F, Dickinson LM. Stakeholder benefit from depression disease management: Differences by rurality? *The Journal of Behavioral Health Services and Research* 2011;**38**(1):114–21.

**Rubenstein 2002 *(published data only)***

Clever SL, Ford DE, Rubenstein LV, Rost KM, Meredith LS, Sherbourne CD, et al. Primary care patients’ involvement in decision-making is associated with improvement in depression. *Medical Care* 2006;**44**(5):398–405.

Nutting PA, Dickinson LM, Rubenstein LV, Keeley RD, Smith JL, Elliott CE. Improving detection of suicidal ideation among depressed patients in primary care. *Annals of Family Medicine* 2005;**3**(6):529–36.

Rost KM, Duan N, Rubenstein LV, Ford DE, Sherbourne CD, Meredith LS, et al. The Quality Improvement for Depression collaboration: general analytic strategies for a coordinated study of quality improvement in depression care. *General Hospital Psychiatry* 2001;**23**(5):239–53.

*Rubenstein LV, Parker LE, Meredith LS, Altschuler A, dePillis E, Hernandez J, et al. Understanding team-based quality improvement for depression in primary care. *Health Services Research* 2002;**37**(4):1009–29.

**Simon 2000a *(published data only)***

*Simon GE, VonKorff M, Rutter C, Wagner E. Randomised trial of monitoring, feedback, and management of care by telephone to improve treatment of depression in primary care. *BMJ* 2000;**320**(7234):550–4.

Williams JW Jr. Feedback to physicians plus telephone care management improved outcomes in primary care patients with depression [comment]. *ACP Journal Club* 2000;**133**(2):73.

**Simon 2000b *(published data only)***

* Simon GE, VonKorff M, Rutter C, Wagner E. Randomised trial of monitoring, feedback, and management of care by telephone to improve treatment of depression in primary care. *BMJ* 2000;**320**(7234):550–4.

Williams JW Jr. Feedback to physicians plus telephone care management improved outcomes in primary care patients with depression[comment]. *ACP Journal Club* 2000;**133**(2):73.

**Simon 2004a *(published data only)***

Ludman EJ, Simon GE, Tutty S, Von Korff M. A randomized trial of telephone psychotherapy and pharmacotherapy for depression: continuation and durability of effects. *Journal of Consulting and Clinical Psychology* 2007;**75**(2):257–66.

Simon GE, Ludman EJ, Rutter C. Incremental benefit and cost of telephone care management and telephone psychotherapy for depression in primary care. *Archives of General Psychiatry* 2009; **66**(10):1081–9.

*Simon GE, Ludman EJ, Tutty S, Operskalski B, Von Korff M. Telephone psychotherapy and telephone care management for primary care patients starting antidepressant treatment: a randomized controlled trial. *JAMA* 2004;**292**(8):935–42.

Tutty S, Ludman EJ, Simon G. Feasibility and acceptability of a telephone psychotherapy program for depressed adults treated in primary care. *General Hospital Psychiatry* 2005;**27**(6):400–10.

**Simon 2004b *(published data only)***

Ludman EJ, Simon GE, Tutty S, Von Korff M. A randomized trial of telephone psychotherapy and pharmacotherapy for depression: continuation and durability of effects. *Journal of Consulting and Clinical Psychology* 2007;**75**(2):257–66.

Simon GE, Ludman EJ, Rutter C. Incremental benefit and cost of telephone care management and telephone psychotherapy for depression in primary care. *Archives of General Psychiatry* 2009; **66**(10):1081–9.

* Simon GE, Ludman EJ, Tutty S, Operskalski B, Von Korff M. Telephone psychotherapy and telephone care management for primary care patients starting antidepressant treatment: a randomized controlled trial. *JAMA* 2004;**292**(8):935–42.

Tutty S, Ludman EJ, Simon G. Feasibility and acceptability of a telephone psychotherapy program for depressed adults treated in primary care. *General Hospital Psychiatry* 2005;**27**(6):400–10.

**Simon 2011 *(published data only)***

Simon GE. Pilot trial of depression care management by electronic secure messaging [feasibility of depression care management by E-Mail][NCT00755235]. [www.clinicaltrials.gov/ct2/show/NCT00755235](http://www.clinicaltrials.gov/ct2/show/NCT00755235) (Accessed 3May2012).

* Simon GE, Ralston JD, Savarino J, Pabiniak C, Wentzel C, Operskalski BH. Randomized trial of depression follow-up care by online messaging. *Journal of General Internal Medicine* 2011;**26**(7):698–704.

**Smit 2006a *(published data only)***

Conradi HJ, deJonge P, Kluiter H, Smit A,vanderMeer K, Jenner JA, et al. Enhanced treatment for depression in primary care: long-term outcomes of a psycho-educational prevention program alone and enriched with psychiatric consultation or cognitive behavioral therapy. *Psychological Medicine* 2007;**37**(6):849–62.

deJonge P, Conradi HJ,Kaptein KI,BocktingCL,Korf J, Ormel J. Duration of subsequent episodes and periods of recovery in recurrent major depression [INSTEL trial]. *Journal of Affective Disorders* 2010;**125**(1-3):141–5.

* Smit A, Kluiter H,Conradi HJ, van der Meer K, Tiemens BG, Jenner JA, et al. Short-term effects of enhanced treatment for depression in primary care: Results from a randomized controlled trial. *Psychological Medicine* 2006;**36**(1):15–26.

Smit A, Tiemens BG, Ormel J, Kluiter H, Jenner JA, van der Meer K, et al. Enhanced treatment for depression in primary care: First year results on compliance, self-efficacy, the use of antidepressants and contacts with the primary care physician. *Primary Care and Community Psychiatry* 2005;**10** (2):39–49.

Stant AD, Ten Vergert EM, Kluiter H, Conradi HJ, Smit A, Ormel J. Cost-effectiveness of a psychoeducational relapse prevention program for depression in primary care. *Journal of Mental Health Policy and Economics* 2009;**12**(4):195-217,220.

**Smit 2006b *(published data only)***

Conradi HJ, deJonge P, Kluiter H, Smit A,vanderMeer K, Jenner JA, et al. Enhanced treatment for depression in primary care: long-term outcomes of a psycho-educational prevention program alone and enriched with psychiatric consultation or cognitive behavioral therapy. *Psychological Medicine* 2007;**37**(6):849–62.

deJonge P, Conradi HJ,Kaptein KI,BocktingCL,Korf J, Ormel J. Duration of subsequent episodes and periods of recovery in recurrent major depression [INSTEL trial]. *Journal of Affective Disorders* 2010;**125**(1-3):141–5.

* Smit A, Kluiter H,Conradi HJ, van der Meer K, Tiemens BG, Jenner JA, et al. Short-term effects of enhanced treatment for depression in primary care: Results from a randomized controlled trial. *Psychological Medicine* 2006;**36**(1):15–26.

Smit A, Tiemens BG, Ormel J, Kluiter H, Jenner JA, van der Meer K, et al. Enhanced treatment for depression in primary care: First year results on compliance, self-efficacy, the use of antidepressants and contacts with the primary care physician. *Primary Care and Community Psychiatry* 2005;**10** (2):39–49.

Stant AD, Ten Vergert EM, Kluiter H, Conradi HJ, Smit A, Ormel J. Cost-effectiveness of a psychoeducational relapse prevention program for depression in primary care. *Journal of Mental Health Policy and Economics* 2009;**12**(4):195-217,220.

**Smit 2006c *(published data only)***

Conradi HJ, deJonge P, Kluiter H, Smit A,vanderMeer K, Jenner JA, et al. Enhanced treatment for depression in primary care: long-term outcomes of a psycho-educational prevention program alone and enriched with psychiatric consultation or cognitive behavioral therapy. *Psychological Medicine* 2007;**37**(6):849–62.

deJonge P, Conradi HJ,Kaptein KI,BocktingCL,Korf J, Ormel J. Duration of subsequent episodes and periods of recovery in recurrent major depression [INSTEL trial]. *Journal of Affective Disorders* 2010;**125**(1-3):141–5.

* Smit A, Kluiter H,Conradi HJ, van der Meer K, Tiemens BG, Jenner JA, et al. Short-term effects of enhanced treatment for depression in primary care: Results from a randomized controlled trial. *Psychological Medicine* 2006;**36**(1):15–26.

Smit A, Tiemens BG, Ormel J, Kluiter H, Jenner JA, van der Meer K, et al. Enhanced treatment for depression in primary care: First year results on compliance, self-efficacy, the use of antidepressants and contacts with the primary care physician. *Primary Care and Community Psychiatry* 2005;**10** (2):39–49.

Stant AD, Ten Vergert EM, Kluiter H, Conradi HJ, Smit A, Ormel J. Cost-effectiveness of a psychoeducational relapse prevention program for depression in primary care. *Journal of Mental Health Policy and Economics* 2009;**12**(4):195-217,220.

**Strong 2008 *(published data only)***

Forchuk C. A nurse-delivered intervention was effective for depression in patients with cancer. *Evidence-Based Nursing* 2009;**12**(1):17.

* Strong V, Waters R, Hibberd C, Murray G, Wall L, Walker J, et al. Management of depression for people with cancer (SMaRToncology 1): a randomised trial [see comment]. *Lancet* 2008;**372**(9632):40–8.

Van der Feltz-Cornelis CM. A nurse delivered management programme for depression in people with cancer reduces depressive symptoms compared with usual care. *Evidence-Based Mental Health* 2009;**12**(1):9.

Walker J, Sharpe M. Depression Care for People with Cancer: a collaborative care intervention. *General Hospital Psychiatry* 2009;**31**(5):436–41.

Walker M, Walker L, Walker A, Bateman J, Braid F, Hebblewhite C, et al. Preventing psychiatric morbidity in people with cancer. Psycho-Oncology. Proceedings of the 11th World Congress of Psycho-Oncology of the International Psycho-Oncology Society, IPOS; 2009 Jun 21-24;Vienna,Austria. 2009; Vol. 18:S238.

**Swindle 2003 *(published data only)***

*Swindle RW, Rao JK, Helmy A, Plue L, Zhou XH, Eckert GJ, et al. Integrating clinical nurse specialists into the treatment of primary care patients with depression. *International Journal of Psychiatry in Medicine* 2003;**33**(1): 17–37.

Weinberger M. An integrated model of primary care in mental health. http:// [www.clinicaltrials.gov](http://www.clinicaltrials.gov) /ct2/show/ NCT00013260 (Accessed3May2012).

**Uebelacker 2011 *(published data only)***

*Uebelacker LA, Marootian BA, Tigue P, Haggarty R, Primack JM, Miller IW. Telephone Depression Care Management for Latino Medicaid Health Plan Members: A Pilot Randomized Controlled Trial. *The Journal of Nervous and Mental Disease* 2011;**199**(9):678-83.

**Unutzer 2002 *(published data only)***

Apesoa Varano EC, Hinton L, Barker JC, Unutzer J. Clinician approaches and strategies for engaging older men in depression care. *American Journal of Geriatric Psychiatry* 2010;**18**(7):586–95.

Arean PA, Ayalon L, Hunkeler E, Lin EH, Tang L, Harpole L, et al. Improving depression care for older, minority patients in primary care. *Medical Care* 2005;**43**(4):381–90.

Arean PA, Gum AM, Tang L, Unutzer J. Service use and outcomes among elderly persons with low incomes being treated for depression. *Psychiatric Services* 2007;**58**(8): 1057–64.

Bao Y, Casalino LP, Ettner SL, Bruce ML, Solberg LI, Unutzer J. Designing payment for collaborative care for depression in primary care. *Health Services Research* 2011; **46**(5):1436–51.

Blasinsky M, Goldman HH, Unutzer J. Project IMPACT: a report on barriers and facilitators to sustainability. Ad*ministration and Policy in Mental Health* 2006;**33**(6): 718–29.

Buist-Bouwman M A. Collaborative care management improves physical functioning in older people with depression. *Evidence-Based Mental Health* 2005;**8**(4):106.

Chan D, Fan MY, Unutzer J. Long-term effectiveness of collaborative depression care in older primary care patients with and without PTSD symptoms. *International Journal of Geriatric Psychiatry* 2011;**26**(7):758–64.

Conn DK. Collaborative care depression management for older adults: level of comorbidity does not affect outcome. *Evidence-Based Mental Health* 2005;**8**(4):105.

Fann JR, Fan MY, Unutzer J. Improving primary care for older adults with cancer and depression. *Journal of General Internal Medicine* 2009;**24**(Suppl2):S417–24.

Gensichen J. IMPACT collaborative care improves depression in elderly patients in primary care in the longer term. *Evidence-Based Mental Health* 2006;**9**(3):76.

Gilbody S M. IMPACT collaborative care programme reduces suicide ideation in depressed older adults. *Evidence-Based Mental Health* 2007;**10**(2):51.

Goldstein KM, Harpole LH, Stechuchak KM, Coffman CJ, Bosworth HB, Steffens DC, et al. Hormone therapy does not affect depression severity in older women. *American Journal of Geriatric Psychiatry* 2005;**13**(7):616–23.

Grypma L, Haverkamp R, Little S ,Unutzer J. Taking an evidence-based model of depression care from research to practice: making lemonade out of depression. *General Hospital Psychiatry* 2006;**28**(2):101–7.

Gum AM, Arean PA, Hunkeler E, Tang L, Katon W, Hitchcock P, et al. Depression treatment preferences in older primary care patients. *Gerontologist* 2006;**46**(1):14–22.

Hegel MT, Imming Jr,Cyr-Provost M, Noel PH, Arean PA, Unutzer J. Role of behavioural health professionals in a collaborative stepped care treatment model for depression in primary care: Project IMPACT. *Families, Systems and Health* 2002;**20**(3):265–77.

Hinton L, Zweifach M, Oishi S, Tang L, Unutzer J. Gender disparities in the treatment of late-life depression: Qualitative and quantitative findings from the IMPACT trial. *American Journal of Geriatric Psychiatry* 2006;**14**(10): 884–92.

Hunkeler EM ,Katon W, Tang L, Williams JWJ, Kroenke K, Lin EH, et al. Long term outcomes from the IMPACT randomised trial for depressed elderly patients in primary care[comments in: Evid Based Ment Health. 2006 Aug; 9(3):76; PMID: 16868195 and BMJ. 2006 Feb 4;332 (7536):249-50; PMID:16455698]. *BMJ* 2006;**332**(7536):259–63.

Katon W, Unutzer J, Fan MY, Williams JWJ, Schoenbaum M, Lin EH, et al. Cost-effectiveness and net benefit of enhanced treatment of depression for older adults with diabetes and depression. *Diabetes Care* 2006;**29**(2):265–70.

Katon WJ, Fan MY, Lin EH, Unutzer J. Depressive symptom deterioration in a large primary care-based elderly cohort. American Journal of Geriatric Psychiatry 2006;**14**(3):246–54.

Lin EH, Katon W, VonKorff M, Tang L, Williams JW, Jr, et al. Effect of improving depression care on pain and functional outcomes among older adults with arthritis: a randomized controlled trial. *JAMA*2003;**290**(18):2428–9.

Lin EH, Tang L, Katon W, Hegel MT, Sullivan MD, Unutzer J. Arthritis pain and disability: response to collaborative depression care. *General Hospital Psychiatry* 2006;**28**(6):482–6.

Lin EHB. Depression and Osteoarthritis. *American Journal of Medicine* 2008;**121**(11Suppl2):S16–9.

Lowe B, Unutzer J, Callahan CM, Perkins AJ, Kroenke K. Monitoring depression treatment outcomes with the patient health questionnaire-9. *Medical Care* 2004;**42**(12): 1194–201.

Noel PH, Williams Jr JW, Unutzer J, Worcbel J, Lee S, Cornell J,et al. Depression and comorbid illness in elderly primary care patients: Impact on multiple domains of health status and well-being. *Annals of Family Medicine* 2004;**2**(6):555–62.

Oxman TE. Collaborative care may improve depression management in older adults. Evidence-Based Mental Health 2003;**6**(3):86.

Pigeon WR, Hegel M, Unutzer J, Fan MY, Sateia MJ, Lyness JM, et al. Is insomnia a perpetuating factor for late life depression in the IMPACT cohort?. *Sleep* 2008;**31**(4): 481–8.

Price J. Collaborative care improves health outcomes in older people with depression and arthritis. *Evidence-Based Mental Health* 2004;**7**(2):45.

Simon G. Collaborative care for depression: is effective in older people, as the IMPACT trial shows. *BMJ* 2006;**332**(7536):249–50.

Slimmer L. A collaborative care management programme in a primary care setting was effective for older adults with late life depression. *Evidence-Based Nursing* 2003;**6**(3):91.

Steffens DC, Snowden M, Fan MY, Hendrie H, Katon WJ, Unutzer J. Cognitive impairment and depression outcomes in the IMPACT study. *American Journal of Geriatric Psychiatry* 2006;**14**(5):401–9.

Tang L, Song J, Belin TR, Unutzer J. A comparison of imputation methods in a longitudinal randomized clinical trial. *Statistics in Medicine* 2005;**24**(14):2111–28.

Thielke SM, Fan MY, Sullivan M, Unutzer J. Pain limits the effectiveness of collaborative care for depression. *American Journal of Geriatric Psychiatry* 2007;**15**(8):699–707.

Thompson A, Fan MY, Unutzer J, Katon W. One extra month of depression: The effects of caregiving on depression outcomes in the IMPACT trial. *International Journal of Geriatric Psychiatry* 2008;**23**(5):511–6.

Unutzer J. Improving depression care for older adults. 155th Annual Meeting of the American Psychiatric Association; 2002 May18-23; Philadelphia(PA).2002:No. 45D.

Unutzer J, Choi Y,Cook IA, Oishi S.A Web-based data management system to improve care for depression in a multicenter clinical trial. *Psychiatric Services* 2002;**53**(6): 671.

Unutzer J,Ferrell B, Lin EH, Marmon T. Pharmacotherapy of pain in depressed older adults. *Journal of the American Geriatrics Society* 2004;**52**(11):1916–22.

* Unutzer J, Katon W, Callahan CM, Williams JW Jr, Hunkeler E, Harpole L, et al. Collaborative care management of late-life depression in the primary care setting: a randomized controlled trial. *JAMA* 2002;**288**(22):2836–45.

Unutzer J, Katon W, Callahan CM, Williams JW Jr, Hunkeler E, Harpole L, et al. Depression treatment in a sample of 1,801 depressed older adults in primary care. *JAMA* 2002;**288**(22):2836–45.

Unutzer J, Katon W, Williams JW Jr, Callahan CM, Harpole L, Hunkeler EM, et al. Improving primary care for depression in late life: the design of a multicenter randomized trial. *Medical Care* 2001;**39**(8):785–99.

Unutzer J, Katon WJ, Fan MY, Schoenbaum MC, Lin EH, Della Penna RD, et al. Long-term cost effects of collaborative care for late-life depression. *American Journal of Managed Care* 2008;**14**(2):95–100.

Unutzer J, Powers D, Katon W, Langston C. From establishing an evidence-based practice to implementation in real-world settings: IMPACT as a case study. *Psychiatric Clinics of North America* 2005;**28**(4):1079–92.

Unutzer J, Tang L, Oishi S, Katon W, Williams JWJ, Hunkeler E, et al. Reducing suicidal ideation in depressed older primary care patients. *Journal of the American Geriatrics Society* 2006;**54**(10):1550–6.

van Leeuwen Williams E, Unutzer J, Lee S, Noel PH. Collaborative depression care for the old-old: findings from the IMPACT trial. *American Journal of Geriatric Psychiatry* 2009;**17**(12):1040–9.

Vannoy SD, Arean P, Untzer J. Advantages of using estimated depression-free days for evaluating treatment efficacy. *Psychiatric Services* 2010;**61**(2):160–3.

Vannoy SD, Duberstein P, Cukrowicz K, Lin E, Fan MY, Unutzer J. The relationship between suicide ideation and late-life depression. *American Journal of Geriatric Psychiatry* 2007;**15**(12):1024–33.

Voils C, Olsen M, Williams Jr J. Identifying depressed older adults in primary care: A secondary analysis of a multisite randomized controlled trial. *Primary Care Companion to the Journal of Clinical Psychiatry* 2008;**10**(1):9–14.

Williams JW Jr, Katon W, Lin EHB, Noel PH, Worchel J, Cornell J, et al. Improving patient care: The effectiveness of depression care management on diabetes-related outcomes in older patients. *Annals of Internal Medicine* 2004;**140**(12): 1015.

**Vera 2010 *(published data only)***

Vera M, Juarbe D. Depression Treatment in General Medical Settings. http:// [www.clinicaltrials.gov](http://www.clinicaltrials.gov) /ct2/show/ NCT00797901(Accessed3May2012).

*Vera M, Perez-Pedrogo C, Huertas SE, Reyes-Rabanillo ML, Juarbe D, Huertas MA, et al. Collaborative care for depressed patients with chronic medical conditions: a randomized trial in Puerto Rico. *Psychiatric Services* 2010;**61**(2):144.

**Vlasveld 2011 *(published data only)***

Vlasveld MC, Anema JR, Beekman AT, vanMechelen W, Hoedeman R, van Marwijk HW, et al. Multidisciplinary collaborative care for depressive disorder in the occupational health setting: design of a randomised controlled trial and cost-effectiveness study. *BMC Health Services Research* 2008;**8**:99.

*Vlasveld M, Van der Feltz-Cornelis C, Adèr H, Anema J, Hoedeman R, Van Mechelen W, et al. Collaborative care for major depressive disorder in an occupational healthcare setting. *The British Journal of Psychiatry* 2012;**200**(6):510-1.

**Wells 2000a (*published data only)***

Callahan CM, Kroenke K, Counsell SR, Hendrie HC, Perkins AJ, Katon W, et al. Treatment of depression improves physical functioning in older adults. *Journal of the American Geriatrics Society* 2005;**53**(3):367–73.

Clever SL, Ford DE, Rubenstein LV, Rost KM, Meredith LS, Sherbourne CD, et al. Primary care patients’ involvement in decision-making is associated with improvement in depression. *Medical Care* 2006;**44**(5):398–405.

Dwight-Johnson M, Unutzer J, Sherbourne C, Tang L, Wells KB. Can quality improvement programs for depression in primary care address patient preferences for treatment? Medical Care 2001;**39**(9):934–44.

Fraser SA, Kroenke K, Callahan CM, Hui SL, Williams JWJ, Unutzer J. Low yield of thyroid-stimulating hormone testing in elderly patients with depression. *General Hospital Psychiatry* 2004;**26**(4):302–9.

Halpern J, Johnson MD, Miranda J, Wells KB. The partners in care approach to ethics outcomes in quality improvement programs for depression. *Psychiatric Services* 2004;**55**(5): 532–9.

Harpole LH, Stechuchak KM, Saur CD, Steffens DC, Unutzer J, Oddone E. Implementing a disease management intervention for depression in primary care: a random work sampling study. *General Hospital Psychiatry* 2003;**25**(4):238–45.

Harpole LH, Williams Jr JW, Olsen MK, Stechuchak KM, Oddone E, Callahan CM, et al. Improving depression outcomes in older adults with comorbid medical illness. *General Hospital Psychiatry* 2005;**27**(1):4–12.

Hegel MT, Unutzer J, Tang L, Arean PA, Katon W, Noel PH, et al. Impact of comorbid panic and posttraumatic stress disorder on outcomes of collaborative care for late-life depression in primary care. *American Journal of Geriatric Psychiatry* 2005;**13**(1):48–58.

Jaycox LH, Miranda J, Meredith LS, Duan N, Benjamin B, Wells K. Impact of a primary care quality improvement intervention on use of psychotherapy for depression. *Mental Health Services Research* 2003;**5**(2):109–20.

Johnson MD, Meredith LS, Hickey SC, Wells KB. Influence of patient preference and primary care clinician proclivity for watchful waiting on receipt of depression treatment. G*eneral Hospital Psychiatry* 2006;**28**(5):379–86.

Katon WJ, Schoenbaum M, Fan MY, Callahan CM, Williams JJ, Hunkeler E, et al. Cost-effectiveness of improving primary care treatment of late-life depression. *Archives of General Psychiatry* 2005;**62**(12):1313–20.

Klap R, Tang L, Schell T, Wells K, Sherbourne C, Miranda J, et al. How quality improvement interventions for depression affect stigma concerns over time: A nine-year follow-up study. *Psychiatric Services* 2009;**60**(2):258–61.

Koike AK, Unutzer J, Wells KB. Improving the care for depression in patients with comorbid medical illness. *American Journal of Psychiatry* 2002;**159**(10):1738–45.

Levine S, Unutzer J, Yip JY, Hoffing M, Leung M, Fan M-Y, et al. Physicians’ satisfaction with a collaborative disease management program for late-life depression in primary care. *General Hospital Psychiatry* 2005;**27**(6):383–91.

Masaquel A, Wells K, Ettner SL. How does the persistence of depression influence the continuity and type of health insurance and coverage limits on mental health therapy? *Journal of Mental Health Policy and Economics* 2007;**10**(3): 133–44.

Meredith LS, Cheng WJY, Hickey SC, Dwight-Johnson M. Factors associated with primary care clinicians’ choice of a watchful waiting approach to managing depression. *Psychiatric Services* 2007;**58**(1):72–8.

Meredith LS, Jackson-Triche M, Duan N, Rubenstein LV, Camp P, Wells KB. Quality improvement for depression enhances long-term treatment knowledge for primary care clinicians. *Journal of General Internal Medicine.* 2000;**15**(12):868–77.

Meredith LS, Orlando M, Humphrey N, Camp P, Sherbourne CD. Are better ratings of the patient-provider relationship associated with higher quality care for depression? *Medical Care* 2001;**39**(4):349–60.

Miranda J, Duan N, Sherbourne C, Schoenbaum M, Lagomasino I, Jackson-Triche M, et al. Improving care for minorities: can quality improvement interventions improve care and outcomes for depressed minorities? Results of a randomized controlled trial. *Health Services Research* 2003; **38**(2):613–30.

Miranda J, Schoenbaum M, Sherbourne C, Duan N, Wells K. Effects of primary care depression treatment on minority patients’ clinical status and employment. *Archives of General Psychiatry* 2004;**61**(8):827–34.

Oishi SM, Shoai R, Katon W, Callahan C, Unutzer J, Arean P, et al. Impacting late life depression: Integrating a depression intervention into primary care. *Psychiatric Quarterly* 2003;**74**(1):75–89.

Roeloffs C, Sherbourne C, Unutzer J, Fink A, Tang L, Wells KB. Stigma and depression among primary care patients. *General Hospital Psychiatry* 2003;**25**(5):311–5.

Rost KM, Duan N, Rubenstein LV, Ford DE, Sherbourne CD, Meredith LS, et al. The Quality Improvement for Depression collaboration: general analytic strategies for a coordinated study of quality improvement in depression care. *General Hospital Psychiatry* 2001;**23**(5):239–53.

Rubenstein LV, Jackson-Triche M, Unutzer J, Miranda J, Minnium K, Pearson ML, et al. Evidence-based care for depression in managed primary care practices. *Health Affairs* 1999;**18**(5):89–105.

Schoenbaum M, Belin T, Wells K. Improving primary care depression treatment: the effect on patients’ employment. *Association for Health Services Research* 1999;**16**:87.

Schoenbaum M, Miranda J, Sherbourne C, Duan N,Wells K. Cost-effectiveness of interventions for depressed Latinos. *Journal of Mental Health Policy and Economics* 2004;**7**(2): 69–76.

Schoenbaum M, Sherbourne C, Wells K. Gender patterns in cost effectiveness of quality improvement for depression: Results of a randomized, controlled trial. *Journal of Affective Disorders* 2005;**87**(2-3):319–25.

Schoenbaum M, Unutzer J, McCaffrey D, Duan N, Sherbourne C, Wells KB. The effects of primary care depression treatment on patients’ clinical status and employment. *Health Services Research* 2002;**37**(5):1145–58.

Schoenbaum M, Unutzer J, Sherbourne C, Duan N, Rubenstein LV, Miranda J, et al. Cost-effectiveness of practice-initiated quality improvement for depression: results of a randomized controlled trial. *JAMA* 2001;**286** (11):1325–30.

Sherbourne CD, Edelen MO, Zhou A, Bird C, Duan N, Wells KB. How a therapy-based quality improvement intervention for depression affected life events and psychological well-beingovertime: a 9-year longitudinal analysis. *Medical Care* 2008;**46**(1):78–84.

Sherbourne CD, Schoenbaum M, Wells KB, Croghan TW. Characteristics, treatment patterns, and outcomes of persistent depression despite treatment in primary care. *General Hospital Psychiatry* 2004;**26**(2):106–14.

Sherbourne CD, Weiss R, Duan N, Bird C, Wells K. Do the effects of quality improvement for depression differ for men and women? *Quality of Life Research* 2003;**12**(7):767.

Sherbourne CD, Weiss R, Duan N, Bird CE, Wells KB. Do the effects of quality improvement for depression care differ for men and women? Results of a group-level randomized controlled trial. *Medical Care* 2004;**42**(12):1186–93.

Sherbourne CD, Wells KB, Duan N, Miranda J, Unutzer J, Jaycox L, et al. Long-term effectiveness of disseminating quality improvement for depression in primary care. *Archives of General Psychiatry* 2001;**58**(7):696–703.

Sherbourne DC, Unutzer J, Schoenbaum M, Duan N, Lenert LA, Sturm R, et al. Can utility-weighted health related quality-of-life estimates capture health effects of quality improvement for depression? *Medical Care* 2001;**39**(11):1246–59.

Unutzer J. Collaborative care for late-life depression. International Psychogeriatrics. Proceedings of the International Psychogeriatric Association Eleventh International Congress; 2003 Aug17-22 2003; Chicago (IL).2003; Vol. 15.

Unutzer J. Collaborative care for late life depression in primary care: a randomized controlled trial. Proceedings of the14th Annual Meeting of the American Association for GeriatricPsychiatry;2001 23-26 Feb; San Francisco (CA). 2001.

Unutzer J, Rubenstein L, Katon WJ, Tang L, Duan N, Lagomasino IT, et al. Two-year effects of quality improvement programs on medication management for depression. *Archives of General Psychiatry* 2001;**58**(10):935–42.

Velarde F, Lagomasino IT, Miranda J, Sherbourne C, Wells KB. Impact of a depression intervention on US-born and immigrant Latinos. Proceedings of the 158th Annual Meeting of the American Psychiatric Association; 2005 May 21-26; Atlanta(GA). 2005:NR141.

Watkins KE, Paddock SM, Zhang L, Wells KB. Improving care for depression in patients with comorbid substance misuse. *American Journal of Psychiatry* 2006;**163**(1): 125–32.

Wells K, Sherbourne C, Duan N, Unutzer J, Miranda J, Schoenbaum M, et al. Quality improvement for depression in primary care: Do patients with subthreshold depression benefit in the long run? *American Journal of Psychiatry* 2005;**162**(6):1149–57.

Wells KB. The design of partners in care: Evaluating the cost-effectiveness of improving care for depression in primary care. *Social Psychiatry and Psychiatric Epidemiology* 1999;**34**(1):20–9.

Wells KB, Rubenstein LV, Unutzer J, Miranda J, Jackson M. Improving treatment resources for primary care depressed patients in managed care. Proceedings of the 151^st^ Annual Meeting of the American Psychiatric Association; 1998 May 30-June4; Toronto Ontario. 1998.

Wells KB, Schoenbaum M, Duan N, Miranda J, Tang L, Sherbourne C. Cost-effectiveness of quality improvement programs for patients with subthreshold depression or depressive disorder. *Psychiatric Services* 2007;**58**(10): 1269–78.

Wells KB, Sherbourne CD, Miranda J, Tang L ,Benjamin B, Duan N. The cumulative effects of quality improvement for depression on outcome disparities over 9 years: results from a randomized controlled group-level trial. *Medical Care* 2007;**45**(11):1052–9.

Wells KB, Sherbourne CD, Schoenbaum M, Rubenstein LV, Duan N, Meredith L, et al. Improving quality of care, outcomes and employment of depressed primary care patients. *Association for Health Services Research* 1999;**16**:88.

*Wells KB, Sherbourne CD, Schoenbaum N, Duan N, Meredith LS, Unutzer J, Miranda J, Carney M, Rubenstein LV. Impact of disseminating quality improvement programs for depression in managed primary care: a randomized controlled trial [erratum appears in JAMA 2000 Jun28;283 (24):3204]. *JAMA* 2000;**283**(2):212–20.

Wells K, Sherbourne C, Schoenbaum M, Ettner S, Duan N, Miranda J, et al. Five-year impact of quality improvement for depression: results of a group-level randomized controlled trial. *Archives of General Psychiatry* 2004;**61**(4):378–86.

Williams JW Jr, Katon W, Lin EH, Noel PH, Worchel J, Cornell J, et al. The effectiveness of depression care management on diabetes-related outcomes in older patients. *Annals of Internal Medicine* 2004;**140**(12):1015–24.

**Wells 2000b (*published data only)***

Callahan CM, Kroenke K, Counsell SR, Hendrie HC, Perkins AJ, Katon W, et al. Treatment of depression improves physical functioning in older adults. *Journal of the American Geriatrics Society* 2005;**53**(3):367–73.

Clever SL, Ford DE, Rubenstein LV, Rost KM, Meredith LS, Sherbourne CD, et al. Primary care patients’ involvement in decision-making is associated with improvement in depression. *Medical Care* 2006;**44**(5):398–405.

Dwight-Johnson M, Unutzer J, Sherbourne C, Tang L, Wells KB. Can quality improvement programs for depression in primary care address patient preferences for treatment? Medical Care 2001;**39**(9):934–44.

Fraser SA, Kroenke K, Callahan CM, Hui SL, Williams JWJ, Unutzer J. Low yield of thyroid-stimulating hormone testing in elderly patients with depression. *General Hospital Psychiatry* 2004;**26**(4):302–9.

Halpern J, Johnson MD, Miranda J, Wells KB. The partners in care approach to ethics outcomes in quality improvement programs for depression. *Psychiatric Services* 2004;**55**(5): 532–9.

Harpole LH, Stechuchak KM, Saur CD, Steffens DC, Unutzer J, Oddone E. Implementing a disease management intervention for depression in primary care: a random work sampling study. *General Hospital Psychiatry* 2003;**25**(4):238–45.

Harpole LH, Williams Jr JW, Olsen MK, Stechuchak KM, Oddone E, Callahan CM, et al. Improving depression outcomes in older adults with comorbid medical illness. *General Hospital Psychiatry* 2005;**27**(1):4–12.

Hegel MT, Unutzer J, Tang L, Arean PA, Katon W, Noel PH, et al. Impact of comorbid panic and posttraumatic stress disorder on outcomes of collaborative care for late-life depression in primary care. *American Journal of Geriatric Psychiatry* 2005;**13**(1):48–58.

Jaycox LH, Miranda J, Meredith LS, Duan N, Benjamin B, Wells K. Impact of a primary care quality improvement intervention on use of psychotherapy for depression. *Mental Health Services Research* 2003;**5**(2):109–20.

Johnson MD, Meredith LS, Hickey SC, Wells KB. Influence of patient preference and primary care clinician proclivity for watchful waiting on receipt of depression treatment. G*eneral Hospital Psychiatry* 2006;**28**(5):379–86.

Katon WJ, Schoenbaum M, Fan MY, Callahan CM, Williams JJ, Hunkeler E, et al. Cost-effectiveness of improving primary care treatment of late-life depression. *Archives of General Psychiatry* 2005;**62**(12):1313–20.

Klap R, Tang L, Schell T, Wells K, Sherbourne C, Miranda J, et al. How quality improvement interventions for depression affect stigma concerns over time: A nine-year follow-up study. *Psychiatric Services* 2009;**60**(2):258–61.

Koike AK, Unutzer J, Wells KB. Improving the care for depression in patients with comorbid medical illness. *American Journal of Psychiatry* 2002;**159**(10):1738–45.

Levine S, Unutzer J, Yip JY, Hoffing M, Leung M, Fan M-Y, et al. Physicians’ satisfaction with a collaborative disease management program for late-life depression in primary care. *General Hospital Psychiatry* 2005;**27**(6):383–91.

Masaquel A, Wells K, Ettner SL. How does the persistence of depression influence the continuity and type of health insurance and coverage limits on mental health therapy? *Journal of Mental Health Policy and Economics* 2007;**10**(3): 133–44.

Meredith LS, Cheng WJY, Hickey SC, Dwight-Johnson M. Factors associated with primary care clinicians’ choice of a watchful waiting approach to managing depression. *Psychiatric Services* 2007;**58**(1):72–8.

Meredith LS, Jackson-Triche M, Duan N, Rubenstein LV, Camp P, Wells KB. Quality improvement for depression enhances long-term treatment knowledge for primary care clinicians. *Journal of General Internal Medicine.* 2000;**15**(12):868–77.

Meredith LS, Orlando M, Humphrey N, Camp P, Sherbourne CD. Are better ratings of the patient-provider relationship associated with higher quality care for depression? *Medical Care* 2001;**39**(4):349–60.

Miranda J, Duan N, Sherbourne C, Schoenbaum M, Lagomasino I, Jackson-Triche M, et al. Improving care for minorities: can quality improvement interventions improve care and outcomes for depressed minorities? Results of a randomized controlled trial. *Health Services Research* 2003; **38**(2):613–30.

Miranda J, Schoenbaum M, Sherbourne C, Duan N, Wells K. Effects of primary care depression treatment on minority patients’ clinical status and employment. *Archives of General Psychiatry* 2004;**61**(8):827–34.

Oishi SM, Shoai R, Katon W, Callahan C, Unutzer J, Arean P, et al. Impacting late life depression: Integrating a depression intervention into primary care. *Psychiatric Quarterly* 2003;**74**(1):75–89.

Roeloffs C, Sherbourne C, Unutzer J, Fink A, Tang L, Wells KB. Stigma and depression among primary care patients. *General Hospital Psychiatry* 2003;**25**(5):311–5.

Rost KM, Duan N, Rubenstein LV, Ford DE, Sherbourne CD, Meredith LS, et al. The Quality Improvement for Depression collaboration: general analytic strategies for a coordinated study of quality improvement in depression care. *General Hospital Psychiatry* 2001;**23**(5):239–53.

Rubenstein LV, Jackson-Triche M, Unutzer J, Miranda J, Minnium K, Pearson ML, et al. Evidence-based care for depression in managed primary care practices. *Health Affairs* 1999;**18**(5):89–105.

Schoenbaum M, Belin T, Wells K. Improving primary care depression treatment: the effect on patients’ employment. *Association for Health Services Research* 1999;**16**:87.

Schoenbaum M, Miranda J, Sherbourne C, Duan N,Wells K. Cost-effectiveness of interventions for depressed Latinos. *Journal of Mental Health Policy and Economics* 2004;**7**(2): 69–76.

Schoenbaum M, Sherbourne C, Wells K. Gender patterns in cost effectiveness of quality improvement for depression: Results of a randomized, controlled trial. *Journal of Affective Disorders* 2005;**87**(2-3):319–25.

Schoenbaum M, Unutzer J, McCaffrey D, Duan N, Sherbourne C, Wells KB. The effects of primary care depression treatment on patients’ clinical status and employment. *Health Services Research* 2002;**37**(5):1145–58.

Schoenbaum M, Unutzer J, Sherbourne C, Duan N, Rubenstein LV, Miranda J, et al. Cost-effectiveness of practice-initiated quality improvement for depression: results of a randomized controlled trial. *JAMA* 2001;**286** (11):1325–30.

Sherbourne CD, Edelen MO, Zhou A, Bird C, Duan N, Wells KB. How a therapy-based quality improvement intervention for depression affected life events and psychological well-beingovertime: a 9-year longitudinal analysis. *Medical Care* 2008;**46**(1):78–84.

Sherbourne CD, Schoenbaum M, Wells KB, Croghan TW. Characteristics, treatment patterns, and outcomes of persistent depression despite treatment in primary care. *General Hospital Psychiatry* 2004;**26**(2):106–14.

Sherbourne CD, Weiss R, Duan N, Bird C, Wells K. Do the effects of quality improvement for depression differ for men and women? *Quality of Life Research* 2003;**12**(7):767.

Sherbourne CD, Weiss R, Duan N, Bird CE, Wells KB. Do the effects of quality improvement for depression care differ for men and women? Results of a group-level randomized controlled trial. *Medical Care* 2004;**42**(12):1186–93.

Sherbourne CD, Wells KB, Duan N, Miranda J, Unutzer J, Jaycox L, et al. Long-term effectiveness of disseminating quality improvement for depression in primary care. *Archives of General Psychiatry* 2001;**58**(7):696–703.

Sherbourne DC, Unutzer J, Schoenbaum M, Duan N, Lenert LA, Sturm R, et al. Can utility-weighted health related quality-of-life estimates capture health effects of quality improvement for depression? *Medical Care* 2001;**39**(11):1246–59.

Unutzer J. Collaborative care for late-life depression. International Psychogeriatrics. Proceedings of the International Psychogeriatric Association Eleventh International Congress; 2003 Aug17-22 2003; Chicago (IL).2003; Vol. 15.

Unutzer J. Collaborative care for late life depression in primary care: a randomized controlled trial. Proceedings of the14th Annual Meeting of the American Association for GeriatricPsychiatry;2001 23-26 Feb; San Francisco (CA). 2001.

Unutzer J, Rubenstein L, Katon WJ, Tang L, Duan N, Lagomasino IT, et al. Two-year effects of quality improvement programs on medication management for depression. *Archives of General Psychiatry* 2001;**58**(10):935–42.

Velarde F, Lagomasino IT, Miranda J, Sherbourne C, Wells KB. Impact of a depression intervention on US-born and immigrant Latinos. Proceedings of the 158th Annual Meeting of the American Psychiatric Association; 2005 May 21-26; Atlanta(GA). 2005:NR141.

Watkins KE, Paddock SM, Zhang L, Wells KB. Improving care for depression in patients with comorbid substance misuse. *American Journal of Psychiatry* 2006;**163**(1): 125–32.

Wells K, Sherbourne C, Duan N, Unutzer J, Miranda J, Schoenbaum M, et al. Quality improvement for depression in primary care: Do patients with subthreshold depression benefit in the long run? *American Journal of Psychiatry* 2005;**162**(6):1149–57.

Wells KB. The design of partners in care: Evaluating the cost-effectiveness of improving care for depression in primary care. *Social Psychiatry and Psychiatric Epidemiology* 1999;**34**(1):20–9.

Wells KB, Rubenstein LV, Unutzer J, Miranda J, Jackson M. Improving treatment resources for primary care depressed patients in managed care. Proceedings of the 151^st^ Annual Meeting of the American Psychiatric Association; 1998 May 30-June4; Toronto Ontario. 1998.

Wells KB, Schoenbaum M, Duan N, Miranda J, Tang L, Sherbourne C. Cost-effectiveness of quality improvement programs for patients with subthreshold depression or depressive disorder. *Psychiatric Services* 2007;**58**(10): 1269–78.

Wells KB, Sherbourne CD, Miranda J, Tang L ,Benjamin B, Duan N. The cumulative effects of quality improvement for depression on outcome disparities over 9 years: results from a randomized controlled group-level trial. *Medical Care* 2007;**45**(11):1052–9.

Wells KB, Sherbourne CD, Schoenbaum M, Rubenstein LV, Duan N, Meredith L, et al. Improving quality of care, outcomes and employment of depressed primary care patients. *Association for Health Services Research* 1999;**16**:88.

*Wells KB, Sherbourne CD, Schoenbaum N, Duan N, Meredith LS, Unutzer J, Miranda J, Carney M, Rubenstein LV. Impact of disseminating quality improvement programs for depression in managed primary care: a randomized controlled trial [erratum appears in JAMA 2000 Jun28;283 (24):3204]. *JAMA* 2000;**283**(2):212–20.

Wells K, Sherbourne C, Schoenbaum M, Ettner S, Duan N, Miranda J, et al. Five-year impact of quality improvement for depression: results of a group-level randomized controlled trial. *Archives of General Psychiatry* 2004;**61**(4):378–86.

Williams JW Jr, Katon W, Lin EH, Noel PH, Worchel J, Cornell J, et al. The effectiveness of depression care management on diabetes-related outcomes in older patients. *Annals of Internal Medicine* 2004;**140**(12):1015–24.

**Williams 2007 *(published data only)***

*Williams LS, Kroenke K, Bakas T, Plue LD, Brizendine E, Tu W, et al. Care management of poststroke Depression a randomized, controlled trial. *Stroke* 2007;**38**(3):998-1003.

**Yeung 2010 *(published data only)***

*Yeung A, Shyu I, Fisher L, Wu S, Yang H, Fava M. Culturally sensitive collaborative treatment for depressed Chinese Americans in primary care. *American Journal of Public Health* 2010;**100**(12):2397-40
