# Supplementary material for: Characteristics of Effective Collaborative Care for Treatment of Depression: A Systematic Review and Meta-Regression of 74 Randomised Controlled Trials
Source: PLoS One. 2014 Sep 29;9(9):e108114. doi: 10.1371/journal.pone.0108114 (PMC4180075; doi:10.1371/journal.pone.0108114)
Supplement: Results S2 — Sensitivity analysis: Cluster Intraclass correlation coefficient of 0.00. (DOCX) [file pone.0108114.s008.docx]

# Results S2. Sensitivity analysis: Cluster Intraclass correlation coefficient of 0.00

**Multivariable predictors of depressive symptoms (N=84)**

| **Variable** | **Regression Coefficient (95% CI)** | **SE** | **P** |
| --- | --- | --- | --- |
| Recruitment method (Systematic) | -0.12 (-0.26 to 0.02) | .07 | .09 |
| Chronic physical health condition (Present) | -0.11 (-0.22 to 0.01) | .06 | .08 |
| Intervention content (Psychological intervention or both) | -0.11 (-0.20 to -0.02) | .05 | .02 |
| Supervision frequency (Scheduled)^*^ | -0.08 (-0.18 to 0.02) | .05 | .14 |
| Supervision frequency (Not applicable)* | 0.06 (-0.16 to 0.27) | .11 | .60 |
| Intercept (constant) | -0.04 (-0.19 to 0.11) | .08 | .60 |

I²=50.3% (95% CI 36.0% to 61.4%)

^* Compared with the reference category, ad hoc supervision^

**Multivariable predictors of antidepressant use (N=59)**

| **Variable** | **Relative risk (95% CI)** | **SE** | **P** |
| --- | --- | --- | --- |
| Recruitment method (systematic) | 1.41 (1.11 to 1.79) | .17 | .01 |
| Chronic physical health condition (Present)* | 1.33 (1.06 to 1.67) | .15 | .01 |
| Intercept (constant) | 1.08 (0.88 to 1.33) | .11 | .46 |

I²=78.1% (72.1% t0 82.8%)

^*Compared twith the reference category, physical health condition absent^

**The effect of change in anti-depressant use on depressive symptoms**

ß=-0.13, 95% CI -0.27 to -0.0004, p=0.05
