# Supplementary material for: Characteristics of Effective Collaborative Care for Treatment of Depression: A Systematic Review and Meta-Regression of 74 Randomised Controlled Trials
Source: PLoS One. 2014 Sep 29;9(9):e108114. doi: 10.1371/journal.pone.0108114 (PMC4180075; doi:10.1371/journal.pone.0108114)
Supplement: Results S3 — Sensitivity analysis: Cluster Intraclass correlation coefficient of 0.05. (DOCX) [file pone.0108114.s009.docx]

# Results S3. Sensitivity analysis: Cluster Intraclass correlation coefficient of 0.05

**Multivariable predictors of depressive symptoms (N=84)**

| **Variable** | **Regression Coefficient (95% CI)** | **SE** | **P** |
| --- | --- | --- | --- |
| Recruitment method (Systematic) | -0.13 (-0.27 to 0.01) | .07 | .07 |
| Chronic physical health condition (Present) | -0.09 (-0.21 to 0.03) | .06 | .14 |
| Intervention content (Psychological intervention or both) | -0.11 (-0.21 to -0.02) | .05 | .02 |
| Supervision frequency (Scheduled)* | -0.09 (-0.20 to 0.02) | .06 | .11 |
| Supervision frequency (Not applicable) * | 0.06 (-0.16 to 0.28) | .11 | .62 |
| Intercept (constant) | -0.03 (-0.19 to 0.13) | .08 | .70 |

I²=46.3% (95% CI 30.5% to 58.5%)

^* Compared with the reference category, ad hoc supervision^

**Multivariable predictors of antidepressant use (N=59)**

| **Variable** | **Relative risk (95% CI)** | **SE** | **P** |
| --- | --- | --- | --- |
| Recruitment method (systematic) | 1.42 (1.12 to 1.81) | .17 | .004 |
| Chronic physical health condition (Present)* | 1.32 (1.05 to 1.65) | .15 | .02 |
| Intercept (constant) | 1.08 (0.88 to 1.32) | .11 | .46 |

I²=74.1% (95% CI 27.0% to 60.5%)

^*Compared with the reference category, physical health condition absent^

**The effect of change in anti-depressant use on depressive symptoms**

ß=-0.13, 95% CI -0.27 to 0.007, p=0.06
