# Supplementary material for: Characteristics of Effective Collaborative Care for Treatment of Depression: A Systematic Review and Meta-Regression of 74 Randomised Controlled Trials
Source: PLoS One. 2014 Sep 29;9(9):e108114. doi: 10.1371/journal.pone.0108114 (PMC4180075; doi:10.1371/journal.pone.0108114)
Supplement: Results S4 — Sensitivity analysis: Relationship between sample size and effect size. (DOCX) [file pone.0108114.s010.docx]

# Results S4. Sensitivity analysis: Relationship between sample size and effect size

**Outcome variable: Depressive symptoms**

| **Variable** |  | **Regression Coefficient (95% CI)** | **SE** | **P** | **I² (95% CI)** |
| --- | --- | --- | --- | --- | --- |
| *Continuous* |  |  |  |  |  |
| Sample size* |  | 0.0001 (-0.00007 to 0.0003) | .00 | .23 | 62.1 (52.0 to 70.0) |

***model intercepts (constants) not reported

**Outcome variable: Antidepressant use**

| **Variable** |  | **Relative risk (95% CI)** | **SE** | **P** | **I²** |
| --- | --- | --- | --- | --- | --- |
| *Continuous* |  |  |  |  |  |
| Sample size* |  | -0.0001 ( -0.0006 to 0.0003) | .0002 | .66 | 81.0 (76.0 to 84.9 |

***model intercepts (constants) not reported
